# Phosphorylation of FEZ1 by Microtubule Affinity Regulating Kinases regulates its function in presynaptic protein trafficking

**DOI:** 10.1038/srep26965

**Published:** 2016-06-01

**Authors:** Eugenia Butkevich, Wolfgang Härtig, Miroslav Nikolov, Christian Erck, Jens Grosche, Henning Urlaub, Christoph F. Schmidt, Dieter R. Klopfenstein, John Jia En Chua

**Affiliations:** 1 Third Institute of Physics–Biophysics, Georg August University Göttingen, 37077, Göttingen, Germany; 2 Paul Flechsig Institute for Brain Research, University of Leipzig, 04103, Leipzig, Germany; 3 Bioanalytical Mass Spectrometry, Max Planck Institute for Biophysical Chemistry, 37077, Göttingen, Germany; 4 Synaptic Systems, 37079, Göttingen, Germany; 5 Effigos AG, 04103, Leipzig, Germany; 6 Bioanalytics, Department of Clinical Chemistry, University Medical Center Göttingen, 37075 Göttingen, Germany; 7 Research Group in Protein Trafficking in Synaptic Development and Function, Department of Neurobiology, Max Planck Institute for Biophysical Chemistry, 37077 Göttingen, Germany; 8 Department of Physiology, Yong Loo Lin School of Medicine, National University of Singapore; 9 Neurobiology/Ageing Programme, Centre for Life Sciences, National University of Singapore.

## Abstract

Adapters bind motor proteins to cargoes and therefore play essential roles in Kinesin-1 mediated intracellular transport. The regulatory mechanisms governing adapter functions and the spectrum of cargoes recognized by individual adapters remain poorly defined. Here, we show that cargoes transported by the Kinesin-1 adapter FEZ1 are enriched for presynaptic components and identify that specific phosphorylation of FEZ1 at its serine 58 regulatory site is mediated by microtubule affinity-regulating kinases (MARK/PAR-1). Loss of MARK/PAR-1 impairs axonal transport, with adapter and cargo abnormally co-aggregating in neuronal cell bodies and axons. Presynaptic specializations are markedly reduced and distorted in FEZ1 and MARK/PAR-1 mutants. Strikingly, abnormal co-aggregates of unphosphorylated FEZ1, Kinesin-1 and its putative cargoes are present in brains of transgenic mice modelling aspects of Alzheimer’s disease, a neurodegenerative disorder exhibiting impaired axonal transport and altered MARK activity. Our findings suggest that perturbed FEZ1-mediated synaptic delivery of proteins arising from abnormal signalling potentially contributes to the process of neurodegeneration.

Continual synaptic delivery of biological materials is quintessential for the establishment and maintenance of neurological synapses. Axonal transport of discrete vesicular packages containing presynaptic proteins has been observed in neurons at various stages of development. Much of this task is undertaken by members of the Kinesin superfamily of motor proteins (reviewed in McAllister^1^). Impairment of intracellular transport by interference of Kinesin function can inhibit neuritogenesis, synaptogenesis and ultimately result in neuronal death^2^,^3^.

Kinesin-1 is a versatile microtubule plus-end motor participating in many aspects of intracellular trafficking (reviewed in Hirokawa *et al.*^4^). As one of the principal motors in axonal transport, it is involved in moving a variety of cargoes such as mitochondria, receptors, channels, synaptic scaffolding proteins and messenger ribonucleoprotein particles. Most cargoes do not interact directly with motors, but rather utilize adaptors to form a regulated bridge between motor and cargo. For Kinesin-1, an increasing number of such adaptors have been identified. Thus, it is conceivable that each adaptor either possesses a cargo spectrum specific to itself or that partially overlaps with other adaptors. Indeed, studies on a small number of adaptors indicate that some adapters and their cargoes are transported in distinct complexes while other cargoes appear to be promiscuously shared by different adapters^5^,^6^. Whether such a phenomenon exists between other adapters during transport remains unclear.

In addition to broadening its cargo repertoire, adapters can also affect the biophysical properties of the motor and serve as scaffolds to recruit additional components for regulating cargo transport. For instance, binding of JNK interacting proteins (JIPs) to Kinesin-1 relieves motor auto-inhibition and enhances motor performance^7^,^8^. By recruiting JNK pathway kinases, these adapters also enable JNK signalling to influence axonal transport^9^,^10^. Significantly, disruption of these signalling pathways impairs intracellular transport, potentially leading to neurodegeneration^11^,^12^.

The protein FEZ1 has been shown to participate in neuronal development and axon fasciculation, with phosphorylation being essential for its function^13^,^14^,^15^,^16^,^17^. Its precise function in these processes, however, remains unclear. Interestingly, concurrent binding of FEZ1 and JIP1 is sufficient to activate Kinesin-1 motors^8^. Moreover, synaptic delivery of several presynaptic proteins has been shown to involve FEZ1 acting as a Kinesin-1 adapter^18^,^19^. Together, these results suggest that FEZ1 potentially plays a critical role in neuronal development by acting in conjunction with Kinesin-1 to deliver proteins required for neurite outgrowth and synaptic formation or function.

Here, we report that FEZ1 cargoes are highly enriched for synaptic transmission and neurodevelopmental functions. Furthermore, phosphorylation of FEZ1 by microtubule affinity-regulating kinases (MARKs; also known as PAR-1) regulates its function in presynaptic protein trafficking. Noteworthy, *par-1* mutants exhibit axonal transport defects similar to those previously observed in FEZ1 mutants and presynaptic specializations are strongly reduced in both mutants. Abnormal aggregation of unphosphorylated FEZ1, Kinesin-1 and its putative cargoes is also present in the brains of aged wild type and 3XTg-AD mice, a transgenic AD mouse model^20^. Taken together, these results provide mechanistic insights into FEZ1’s roles in neuronal development and synaptic function and suggest that impaired delivery of proteins to synapses via a FEZ1-mediated transport route potentially contributes to the pathogenesis observed during Alzheimer’s disease.

## Results

### FEZ1 cargoes are enriched for synaptic and neurodevelopmental functions

**Figure 1:**
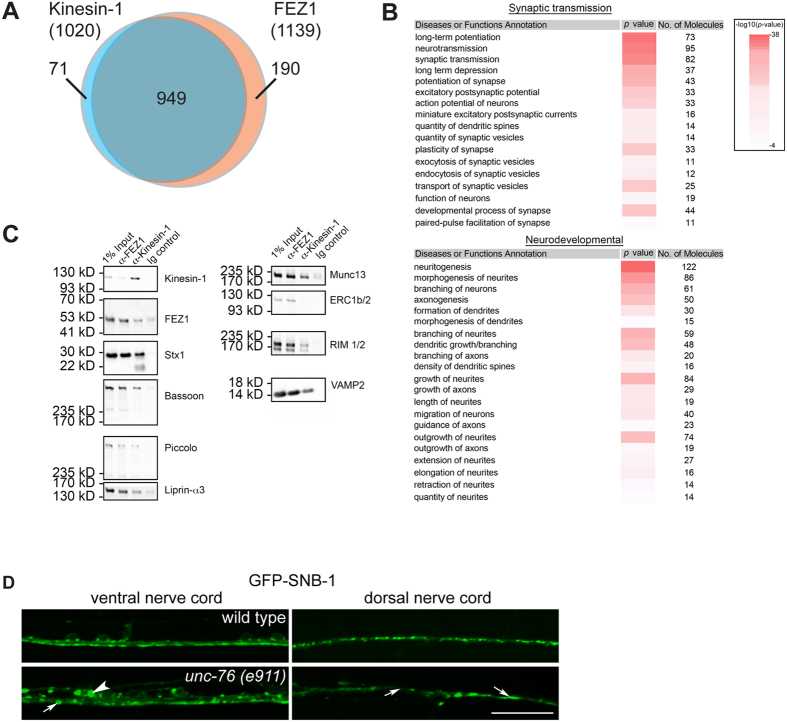
Cargoes transported by FEZ1 are enriched for synaptic and neuronal developmental functions. (**A**) The Venn diagram illustrates the total number of proteins specifically identified in FEZ1 and Kinesin-1 containing vesicles and the number of proteins common to both samples. Data was obtained from 4 independent biological replicates. (**B**) Functional analysis of proteins identified on FEZ1 and Kinesin-1 immunoisolated transport vesicles reveals a significant enrichment of proteins associated with synaptic neurotransmission and neurodevelopmental functions (Refer to Supplementary Table S2 for full list). Data was obtained from 4 independent biological replicates. (**C**) FEZ1 or Kinesin-1 immunoisolates were immunoblotted for presynaptic proteins identified in the IP-MS data. In addition to Syntaxin1 (Stx1), active zone proteins (Bassoon, Piccolo, RIM1/2, Munc13 and Liprin-α) as well as the synaptic vesicle protein VAMP2/Synaptobrevin are also present in these vesicles. (**D**) Presynaptic specializations are perturbed in *unc-76* mutants. GFP-SNB-1 was used here as a marker for presynaptic specializations. In wild type worms, small regularly spaced GFP-SNB-1 punctae can be observed in the ventral as well as the dorsal nerve cords (VNC and DNC, respectively). These punctae disappear in *unc-76* mutants and are replaced by either large abnormal aggregates (arrows in the VNC panel) or diffuse distribution (arrows in the DNC panel). Anomalous cell body aggregates of GFP-SNB-1 in the cell body can also be observed in the VNC (arrowhead in the VNC panel). Scale bar, 20 μm.

To examine the nature of cargoes transport by FEZ1, we immunoisolated FEZ1- and Kinesin-1-containing vesicles from the rat brain cytosol fraction and analysed their contents using label-free quantitative mass spectrometry (see Materials and Methods for details of quantification and data analysis)^21^,^22^. A total of 1020 and 1139 proteins were found to be specifically enriched in the Kinesin-1 and FEZ1 immunoisolated samples, respectively (Fig. 1A, see Supplementary Table S2 for complete list of proteins identified). Of these proteins, an overwhelming majority (93%) were commonly identified in both datasets. This close correlation demonstrates that the functions of FEZ1 and Kinesin-1 are tightly coupled and concurs well with the previous finding that FEZ1 is essential for Kinesin-1 activation and its ability to transport cargo^8^. In additional, known Kinesin-1 accessory components (such as Kinesin light chain 2, JIP3/MAPK8IP3, CRMP2/DPYSL2 and NUDC) and cargoes (amyloid beta precursor protein/APP, GluR2/GRIA2, Munc18/Stxbp1, prion/PRNP, VAMP2, SNAP25 and Stx1) were also present in our data^7^,^19^,^23^,^24^,^25^,^26^,^27^,^28^,^29^,^30^,^31^.

To classify the proteins identified, we subjected the proteins found in both immunoisolations to functional analyses using the Ingenuity Pathway Analysis (IPA) software. Two functional clusters were found to be significantly enriched in our dataset. The first cluster consisted of proteins associated with synaptic transmission while the second cluster corresponded to proteins with functions linked to the development of neurons (Fig. 1B). These results are in agreement with previous reports showing that FEZ1 is required for neurite development while revealing the additional involvement of the adapter in transporting presynaptic proteins. We confirmed the presence of presynaptic proteins detected in our IP-MS analyses by immunoblotting the immunoisolates for Bassoon, Piccolo, Rim, Munc13, Liprin-α and VAMP2/Synaptobrevin (Fig. 1C). These results indicate that presynaptic proteins form an important group of cargoes recognized by the FEZ1-Kinesin-1 transport complex.

If FEZ1 delivers presynaptic proteins, does disruption of FEZ1-mediated axonal transport affect synapses? To answer this question, we generated *Caenorhabditis elegans* transgenic worms expressing GFP-SNB-1 (synaptobrevin 1) which is commonly used as a marker to define presynaptic sites^32^. The distribution of this marker was then imaged in live worms. In wild type animals, GFP-SNB-1 assumes a regular punctate distribution pattern in axons of both dorsal and ventral nerve cords as described previously (Fig. 1D, upper panels). In *unc-76* (orthologue of FEZ1) mutants, GFP-SNB-1 punctae become irregularly spaced and their numbers are greatly reduced (Fig. 1D, arrows in lower panels), a pattern particularly evident in the dorsal nerve cord. Abnormal aggregates of the protein can also be seen to accumulate in neuronal cell bodies and along axons, consistent with SNB-1 being a FEZ1 cargo (Fig. 1D, arrowhead in lower left panel). Thus, impairment of FEZ1 function negatively affects the number and morphology of presynaptic sites most likely via limiting delivery of synaptic components.

### Phosphorylation of FEZ1 at S58 is mediated by MARK/PAR-1

FEZ1 can be rendered non-functional by abrogating its phosphorylation at S58^19^. This affects the adapter’s binding to Kinesin-1 and severely impairs both intracellular and axonal transport^19^,^33^. Given that disruption of FEZ1 function leads to the disorganization and loss of synapses, we decided to search for kinases which are able to phosphorylate S58 to determine the regulatory mechanism involved in regulating FEZ1 function. Phosphorylation at the conserved site (S143) in *Drosophila* FEZ1 is mediated by UNC-51^18^. However, *in vitro* kinase assays using the mammalian UNC-51 orthologues ULK1 or ULK2 against recombinant FEZ1 did not result in S58 phosphorylation. *In silico* motif scan analysis of potential kinases recognizing S58 also did not reveal significant hits. Since the IP-MS data contained a number of Serine and/or Threonine (Ser/Thr) kinases (See Supplementary Table S2), we wondered if some of them might recognize this residue. To determine this, we tested the ability of a commercially available panel of 190 Ser/Thr kinases to phosphorylate a S58-containing FEZ1 peptide (SEIISFKSMEDLVNEF) *in vitro*. The panel contains 14 of the 20 Ser/Thr kinases found in the IP-MS data.

Twelve out of 190 kinases were found to phosphorylate the peptide above threshold (See Supplementary Fig. S1 and Supplementary Table S3). Of these, only proteins from the microtubule-affinity regulating kinases (MARKs) family exhibited significant activity against S58 and were also present in the immunoisolation-MS data. Noteworthy, MARKs are known to regulate intracellular transport in neurons^34^. Also, MARKs and FEZ1 have been implicated in neurite outgrowth^15^,^35^. Hence, MARKs represent prominent kinase candidates responsible for regulating FEZ1-dependent transport.

**Figure 2:**
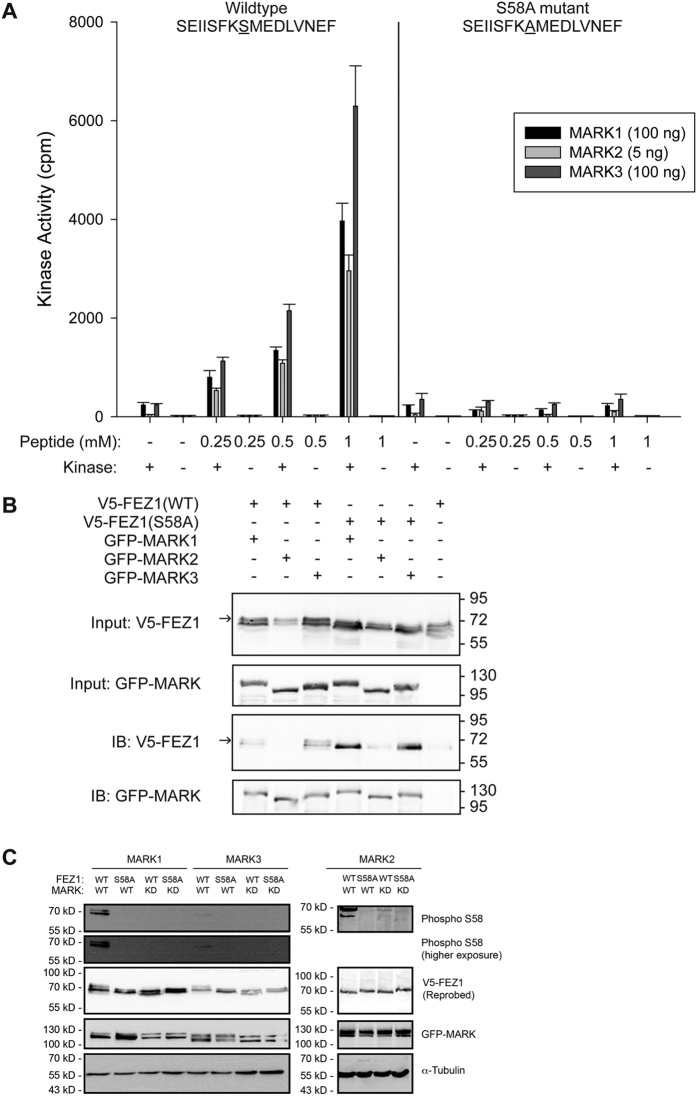
MARKs recognize S58, a site critical for regulating the axonal transport function of FEZ1. (**A**) MARKs 1, 2 and 3 show a dose-dependent increase in kinase activity when exposed to increasing amounts of FEZ1-S58 peptide. Substitution of S58 with an alanine residue in the peptide abrogated kinase activity. n = 3, error bars represent S.E.M. (**B**) Co-expression of wild type V5-tagged FEZ1 with GFP-MARKs in HEK 293 cells resulted in the appearance of a slower migrating band (Input V5-FEZ1, arrow) not observed when the kinases were omitted (last lane) or when the FEZ1 (S58A) mutant was used. The slower migrating band likely represents phosphorylated FEZ1. Immunoprecipitation of MARKs (using anti-GFP antibody) from these lysates reveals that FEZ1 (WT) physically interacts with its cognate kinases. Additionally, FEZ1 (S58A) binds more strongly to these kinases than FEZ1 (WT). In agreement with the abovementioned observations, the slower migrating band can also be observed (arrow) in e.g. FEZ1 (WT) co-immunoprecipitated with GFP-MARK3 but not in FEZ1 (S58A)-GFP-MARK3 co-immunoprecipitations. (**C**) Lysates from HeLa cells co-expressing various combinations of wild type or S58A FEZ1 with wild type (WT) or kinase dead (KD) versions of MARK1, 2 or 3 were probed with a phospho-antibody specifically recognizing phosphorylation of FEZ1 at S58. Wild type FEZ1 is phosphorylated at S58 only when co-expressed with wild type MARKs. Combinations of FEZ1 (S58A) with wild type MARK or wild type FEZ1 with MARK (KD) did not result in phosphorylation of FEZ1 at this site. α-tubulin was probed as a loading control. The second panel from the top shows a longer exposure for the blot detecting phosphorylation of FEZ1 by MARK1 and MARK3.

To determine whether MARK phosphorylation at S58 of FEZ1 was specific, dose-dependent phosphorylation of the FEZ1 peptide was assessed by titrating the amount of peptide against a constant amount of kinase. As a control, we also tested a mutant FEZ1 peptide where S58 was changed to an alanine (SEIISFKAMEDLVNEF). The activities of MARKs exhibited dose-dependency with increasing amount of wild type FEZ1 peptide used (Fig. 2A). Importantly, only background MARK activity was observed when the mutant FEZ1 peptide was used as the substrate, even at the highest peptide concentration. These results confirm that MARKs are able to specifically phosphorylate FEZ1 at S58.

To verify that MARKs are able to recognize native full-length FEZ1, their ability to recognize FEZ1 *in vivo* was tested in two ways. First, co-expression of wild type (WT) FEZ1 with MARKs 1–3 resulted in the appearance of a significantly slower migrating band on immunoblots that likely represents phosphorylated FEZ1 (Fig. 2B, arrow). This slower migrating band was not observed when mutant FEZ1 (S58A) was used or when wild type FEZ1 was expressed in the absence of the kinases. Second, all MARK kinases tested co-precipitated with full-length FEZ1 in co-immunoprecipitation assays (Fig. 2B, IB panels). Remarkably, mutant FEZ1 (S58A) exhibits significantly stronger binding to MARKs as compared to wild type FEZ1. A similar enhancement was also reported for the interaction between MARK2 and its upstream kinase MARKK/TAOK1^36^. We suspect that the inability of MARKs to phosphorylate FEZ1 (S58A) causes the latter to be retained within the substrate binding pocket in the catalytic site of MARKs.

Finally, we directly assessed the phosphorylation status of FEZ1 at S58 upon exposure to wild type versus inactive versions of MARKs. To do this, we generated a phospho-specific antibody against this residue and confirmed its ability to distinguish between wild type FEZ1 phosphorylated at S58 as compared to the FEZ1 (S58A) mutant (see Supplementary Fig. S2). We then co-expressed FEZ1 with either wild type (WT) or the inactive version (KD) of each MARK in HeLa cells. No phosphorylation of FEZ1 at S58 was detected in cell lysates when the inactive version (KD) of each MARK was co-expressed with wild type FEZ1 or when wild type MARKs were co-expressed with the FEZ1 (S58A) mutant (Fig. 2C). In contrast to this, phosphorylation of wild type FEZ1 at S58 was detectable upon co-expression with each wild type MARK tested. Taken together, these results confirm that MARKs are indeed FEZ1 kinases acting on S58.

### Axonal transport and synapses are disrupted in PAR-1 mutants

To determine if MARK phosphorylation of FEZ1 indeed regulates its function in axonal transport, we investigated the effects of loss of MARK function in live *Caenorhabditis elegans.* Unlike mammals, *C. elegans* possesses only one MARK gene (*par-1*) which encodes several MARK/PAR1 isoforms^37^. We analysed transgenic worms co-expressing GFP-UNC-64 and Cerulean-UNC-76 (*C. elegans* orthologues of syntaxin-1 and FEZ1, respectively) in two *par-1* mutant alleles: *ok2001* and *zu310*. The *ok2001* mutant harbours a deletion within the coding exon of the d and e isoforms^38^. In *zu310* mutants, a substitution of an isoleucine by an asparagine affecting all isoforms in the kinase domain results in the production of a kinase exhibiting only residual activity^39^.

**Figure 3:**
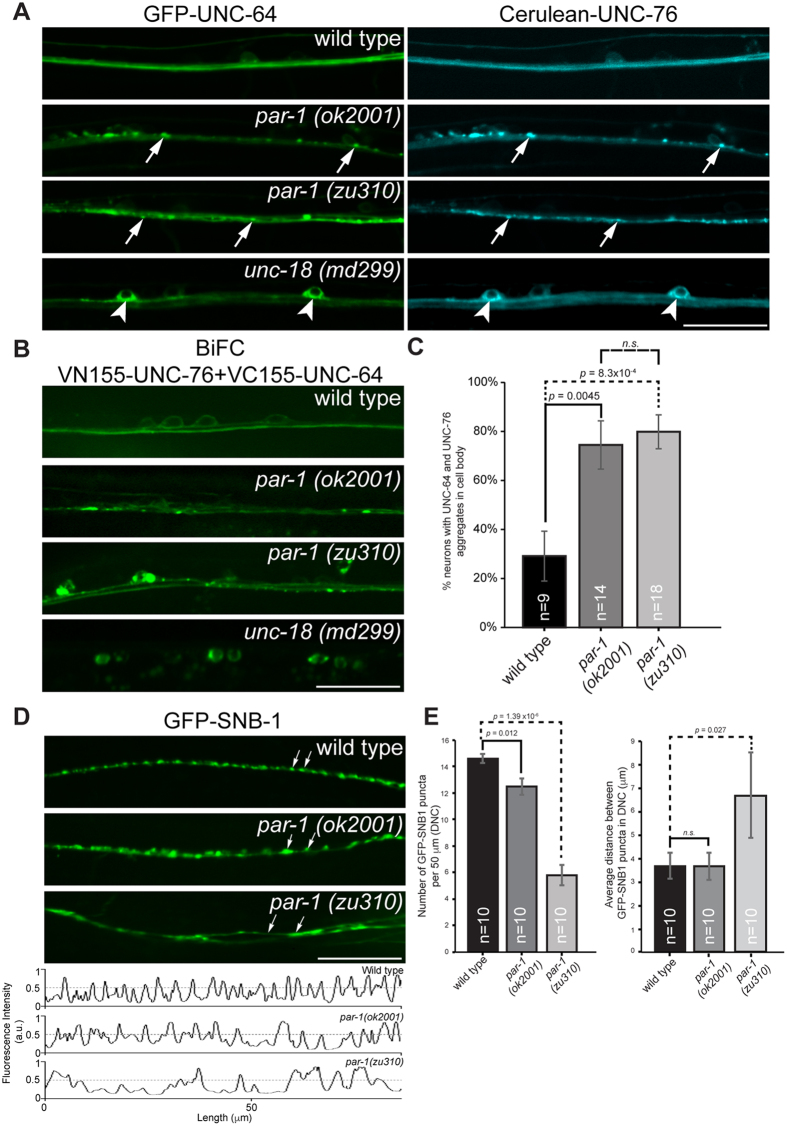
Axonal transport and synaptic defects in *par-1* mutants. (**A**) Localization of GFP-UNC-64 and Cerulean-UNC-76 in ventral nerve cords (VNC) of various *C. elegans* strains. GFP-UNC-64 and Cerulean-UNC-76 are uniformly distributed in VNC of wild type transgenic worms expressing either protein (top panel). Abnormal axonal aggregates (arrows) of both proteins appear in both *par-1* mutants (middle 2 panels). In *unc-18* mutants, strong cell body retention of both proteins (arrowheads) is observed, consistent with UNC-18’s function in ER export of UNC-64 (bottom panel). Scale bar, 20 μm. (**B**) Localization of bimolecular fluorescence complexes (BiFC) containing VN155-UNC-76 and VC155-UNC-64 in various *C. elegans* strains. BiFC complexes exhibit uniform distribution in wild type worms (top panel). Cell body retention of BiFC complexes similar to their singly expressed counterparts in *unc-18* mutants supports that they are not artefacts arising from GFP complementation (bottom panel). *par-1* mutants exhibit abnormal aggregation of the complexes in VNC axons and neuronal cell bodies distinct from those in *unc-18* mutants. Scale bar, 20 μm. (**C**) Quantification of neurons exhibiting cell body aggregates of UNC-64 and UNC-76 as exemplified in (**B**) (*p* < 0.01, *t*-test). Error bars represent S.E.M. (**D**) Defective presynaptic organization in *par-1* mutants. GFP-SNB-1 was used here as a marker for presynaptic specializations. Wild type worms display small regularly spaced GFP-SNB-1 punctae in dorsal nerve cords (DNC). In *par-1* mutants, these punctae are replaced by large abnormal aggregates (arrows, *par-1 (ok2001)* panel) or become diffusely distributed (arrows, *par-1 (zu310)* panel). Anomalies are also observed in VNC (see Supplementary Fig. S3). Scale bar, 20 μm. (**E**) Quantification of presynaptic specializations in wild type and *par-1* mutants. Line scans were drawn along the DNC using ImageJ software (Fig. 3D, bottom panel). Fluorescent intensity peaks greater than 0.5 were considered as genuine GFP-SNB-1 punctae and included for the analyses. GFP-SNB-1 punctae densities along a 50 μm stretch of the DNC and average inter-punctae distances were calculated. *par-1* mutants exhibit significantly lower GFP-SNB-1 densities against wild type worms (*p* < 0.05, *t*-test). Furthermore, *par-1 (zu310)* exhibited larger inter-punctae distances indicative of presynaptic organization defects (*p* < 0.05, *t*-test). Error bars represent S.E.M.

UNC-64 is a known FEZ1 cargo and is uniformly distributed in the axons of ventral nerve chord (VNC) neurons in wild type worms (Fig. 3A, top panels)^19^,^40^. In contrast to this, UNC-64 becomes aggregated in the axons and cell bodies in both *par-1* mutants (*ok2001* and *zu310*) (Fig. 3A, middle two panels). This phenotype is very similar to what we previously observed in *unc-76 (e911)* mutants^19^. Likewise, the distribution of UNC-76 (which is evenly distributed in wild type worms) also becomes punctate in both *par-1* mutants and colocalises with UNC-64 aggregates (Fig. 3A, middle two panels). Colocalisation of the two proteins in these aggregates supports the idea that they indeed represent disrupted transport vesicles arising from impaired axonal transport.

To clarify whether PAR-1 specifically affects axonal transport, we further determined the distribution of UNC-64 and UNC-76 in *unc-18* mutants, where transport of UNC-64 is affected at an earlier step. As previously reported, UNC-64 is primarily retained in the ER in neuronal cell bodies of *unc-18* mutants (Fig. 3A, arrowheads in bottom panels), consistent with a blockage during ER export^40^. Remarkably, UNC-76 also exhibits a similar distribution, suggesting that transport complexes of UNC-64/UNC-76 are already formed at this stage. The striking differences between phenotypes observed in *unc-18* and *par-1* mutants are consistent with the notion that UNC-18 and PAR-1 regulate distinct steps in the trafficking of UNC-64 to synapses (i.e. ER export versus post-export motor transport).

To show that complexes of UNC-64 and UNC-76 are indeed present in these aggregates, we generated bimolecular fluorescence complementation (BiFC) transgenic worms. Here, N- or C-terminally truncated versions of Venus fluorescent protein were fused either protein to create VC155-UNC-64 or VN155-UNC-76 fusion proteins, respectively. Formation of a complex between the interacting proteins reconstitutes the functional Venus protein. The resulting fluorescence allows the subcellular location of these complexes to be determined^41^.

Like their counterparts tagged with intact fluorescent proteins, reconstituted Venus BiFC signal for VC155-UNC-64 + VN155-UNC-76 complexes was uniformly distributed in wild type worms (Fig. 3B, top panel). Retention of BiFC signal in the ER of neuronal cell bodies from *unc-18* mutants provided evidence that these signals indeed represented functional UNC-64 + UNC-76 complexes *in vivo* and that they were not spuriously formed (Fig. 3B, bottom panel). In agreement with what we observed with full-length GFP-tagged proteins, BiFC signal for VC155-UNC-64 + VN155-UNC-76 complexes are strongly aggregated in axons and cell bodies of *par-1* mutants (Fig. 3B, middle 2 panels). We also quantified the amount of neurons exhibiting BiFC signals in their cell bodies in wild type and *par-1* mutants. Significantly more VC155-UNC-64 + VN155-UNC-76 complexes were trapped in the cell bodies of both *par-1* mutants as compared to wild type worms (Fig. 3C). This supports the notion that cargo and adapter are present in the same complexes in aggregates arising from impaired axonal transport in *par-1* mutants.

If loss of PAR-1 disrupts FEZ1-mediated transport, does this defect also cause synapses to be disrupted? To address this, we analysed the localization of presynaptic marker GFP-SNB-1 in these mutants. Like in *unc-76* mutants, the amount of GFP-SNB-1 punctae in the ventral and dorsal nerve cords of *par-1 (zu310)* mutants was reduced and irregularly spaced, at times appearing as large aggregates (Fig. 3D, middle panel and accompanying line scan). *par-1 (ok2001)* mutants also exhibited defects in GFP-SNB-1 distribution, although the phenotype of this mutant was relatively mild (Fig. 3D, bottom panel and accompanying line scan). We further evaluated the extent of this defect on presynaptic sites by quantifying the density of GFP-SNB-1 punctae and the average spacing between them in the dorsal nerve cord. Strikingly, *par-1* mutants exhibited a significantly lower GFP-SNB-1 density as compared to wild type worms (Fig. 3E, left chart). In addition, *par-1 (zu310)*, which corresponds to the stronger allele, also exhibited dramatically larger average distances between intervening GFP-SNB-1 punctae (Fig. 3E, right chart). Thus, loss of the motor adapter as well as perturbation of its function via inactivation of its kinase both leads to similar abnormalities in axonal transport and presynaptic organisation.

### FEZ1 aggregates in the brains of 3XTg-AD mice

In addition to regulating intracellular transport, MARKs have been implicated in Alzheimer’s disease (AD) pathology via their ability to phosphorylate the microtubule-associated protein tau which influences its propensity to bind microtubules and to self-aggregate^42^. Disrupted axonal transport, manifesting as anomalous cargo accumulations in axons, has been observed in AD patients and transgenic mouse models^43^,^44^. Such anomalies were also previously observed in *unc-76*/FEZ1 and *unc-116*/Kinesin-1 mutants^18^,^19^,^45^. Furthermore, altered MARK activity leading to loss of synapses has previously been implicated in AD pathogenesis^46^. Our observation that loss of both FEZ1 and MARK/Par-1 function causes impaired axonal transport and synapse reduction suggests that FEZ1 abnormalities might also occur during AD progression. To investigate this connection, we analysed FEZ1 distribution in the hippocampus of age-matched wild type and 3XTg-AD transgenic mice, an AD transgenic model^20^. In this model, tau abnormalities appear at 9–12 months of age while intraneuronal Aβ aggregates become readily observable beginning at 3 months.

**Figure 4:**
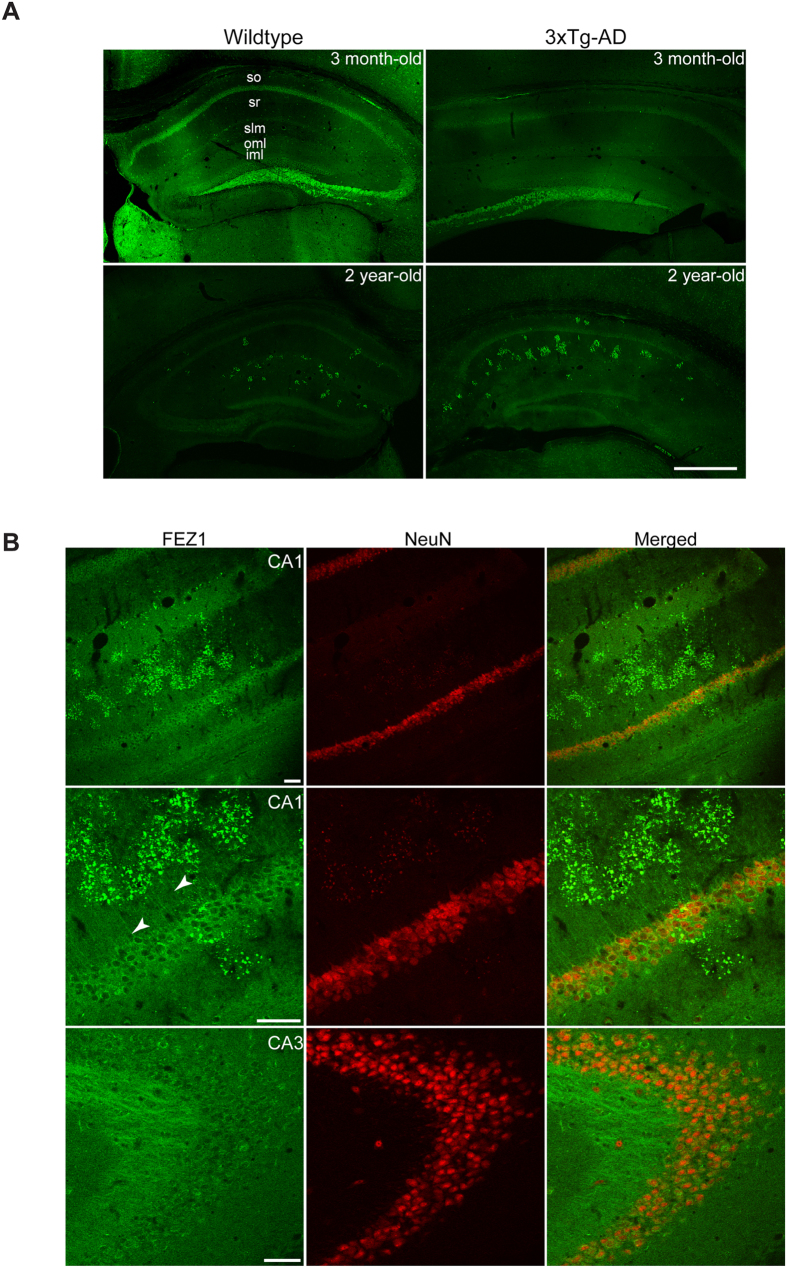
FEZ1 abnormally aggregates in aged as well as in 3XTg-AD mice. (**A**) Low magnification views of the hippocampal region from 3 month-old or 2 year-old wild type or 3XTg-AD mice immunostained for endogenous FEZ1. No aggregation of FEZ1 is observed in 3 month-old wild type mice although slight punctate staining could already be observed in 3XTg-AD mice at a similar age (Supplementary Fig. S5 and Fig. 5, top panel, respectively). By 2 years of age, FEZ1 aggregation is readily observed in both wild type and 3XTg-AD mice, with the latter exhibiting much higher levels of aggregation. Abbreviations: stratum oriens (so), stratum radiatum (sr), stratum lacunosum-moleculare (slm), outer molecular layer (olm) and inner molecular layer (iml) of the dentate gyrus. Scale bar, 500 μm. (**B**) (Top and middle panels) Higher magnification views of the CA1 region from a 2 year-old 3XTg-AD mouse showing FEZ1 aggregation in these regions. Dendritic FEZ1 staining can clearly be observed (arrowheads). (Bottom panel) No aggregation of FEZ1 is observed in CA3 mossy fibre projections. Scale bars, 50 μm.

In agreement with previous studies, FEZ1 expression was detected in hippocampal cornu ammonis (CA) regions 1–3 and in the dentate gyrus (Fig. 4A)^47^. Both neuropil and cell body layer of neurons in the CA regions of 3 month-old and 2 year-old wild type and 3XTg-AD mice display FEZ1 staining, with the latter region showing greater intensity (Fig. 4A). Co-staining with the pan-neuronal marker NeuN confirms the expression of FEZ1 in pyramidal neurons (See Supplementary Fig. S4). While a relatively weaker signal is observed in dendrites projecting from CA1–2 pyramidal neurons, strong staining of mossy fibre projections into the CA3 region indicates that FEZ1 is a predominantly axonal protein (See Supplementary Fig S4; also Fig. 4B). The presence of FEZ1 in both dendrites and axons agrees with previous reports indicating that FEZ1 is involved in regulating the development of both types of processes^13^,^16^.

Unlike in 3 month-old mice, aggregation of FEZ1 is observed in the CA1 neuropil region of 2 year-old wild type mice (Fig. 4A and Supplementary Fig. S4). Age-matched 3XTg-AD mice show an even higher degree of FEZ1 aggregation (Fig. 4A,B, top and middle panels). Interestingly, these aggregates were not observed in CA3 and mossy fibre projections (Fig. 4B, bottom panel). Thus, FEZ1 aggregation occurs as a consequence of ageing but is further aggravated if accompanied by AD pathogenesis. Because FEZ1 aggregation is more pronounced in 3XTg-AD mice, we decided to focus our subsequent studies on these mice.

**Figure 5:**
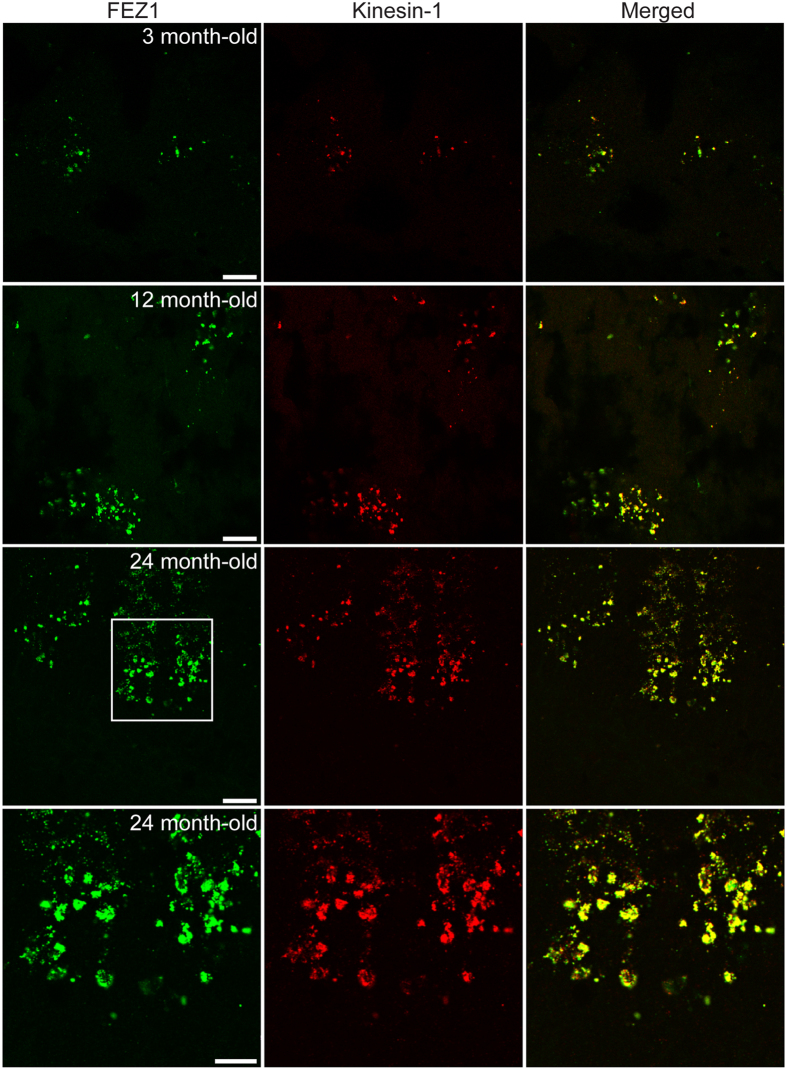
FEZ1 aggregates increase with age in 3XTg-AD mice and contain Kinesin-1. Hippocampi from 3, 12 and 24 month-old 3XTg-AD mice were co-stained with FEZ1 and Kinesin-1. Aggregates containing both proteins could already be observed at 3 months of age and progressively increase with aging and disease progression. Scale bars for the top 3 panels indicate 20 μm. Scale bar for the bottom panel indicates 10 μm.

The extent of FEZ1 aggregation appears to progressively increase with age in 3XTg-AD mice. While small aggregates could be observed at 3 months, this phenomenon continued to intensify at 12 and 24 months (Fig. 5). Remarkably, Kinesin-1 is also present in FEZ1 aggregates. These co-aggregates support our hypothesis that dysfunctional FEZ1-Kinesin-1 contributes to the onset and progression of impaired intracellular transport.

### Known and putative cargoes are present in FEZ1 aggregates

**Figure 6:**
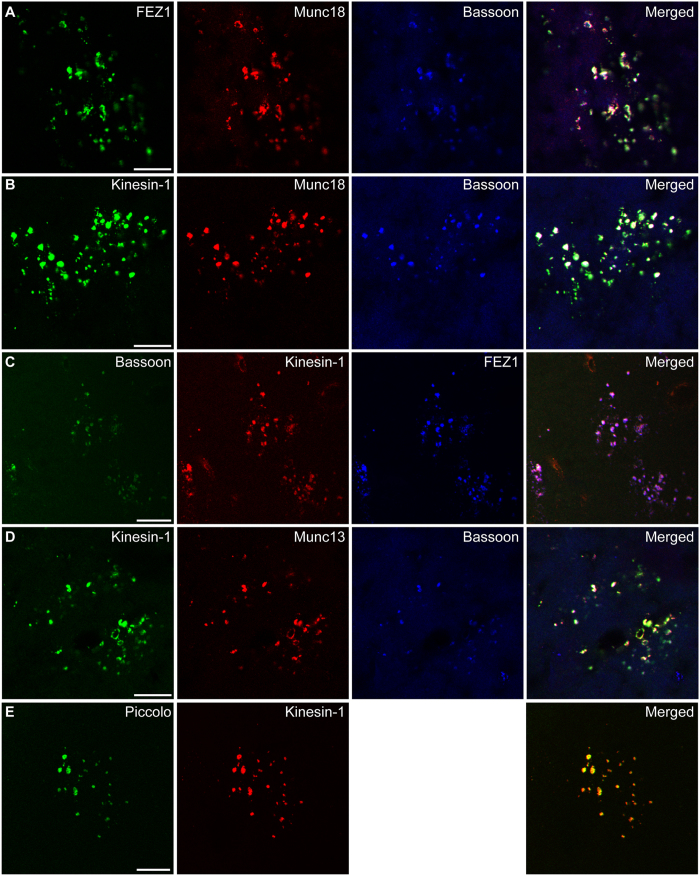
Putative FEZ1-Kinesin-1 cargoes co-aggregate in FEZ1-Kinesin-1 aggregates. Co-immunostaining of known and putative FEZ1-Kinesin-1 presynaptic cargo proteins with FEZ1 or Kinesin-1 in the hippocampus from 2 year-old 3XTg-AD mice. (**A**–**C**) Munc18 and Bassoon are present in FEZ1-Kinesin-1 aggregates. (**D**,**E**) Munc13 and Piccolo are additional components of FEZ1-Kinesin-1 aggregates. Scale bars, 20 μm.

Abnormal synaptic transmission reported for 3XTg-AD mice might be related to reduced delivery of material to the synapse affecting its functions^20^. To determine if FEZ1-Kinesin-1 aggregates could represent trapped motor-cargo complexes, we tested for the presence of putative cargoes identified from our IP-MS data. Indeed, the presynaptic proteins Bassoon and Munc18 colocalised with FEZ1 and Kinesin-1 aggregates in 2 year-old 3XTg-AD mice (Fig. 6A,B). As a control, triple staining of Bassoon, Kinesin-1 and FEZ1 further demonstrated that these proteins colocalised in FEZ1-Kinesin-1 aggregates (Fig. 6C). Two additional putative presynaptic cargoes tested (Munc13 and Piccolo) were also present in FEZ1-Kinesin-1 aggregates (Fig. 6D,E). Therefore, these aggregates very likely represent motor-cargo packages whose delivery to synapses has been prematurely disrupted as a result of impaired intracellular transport.

### Aggregated FEZ1 is unphosphorylated and colocalise with Reelin

**Figure 7:**
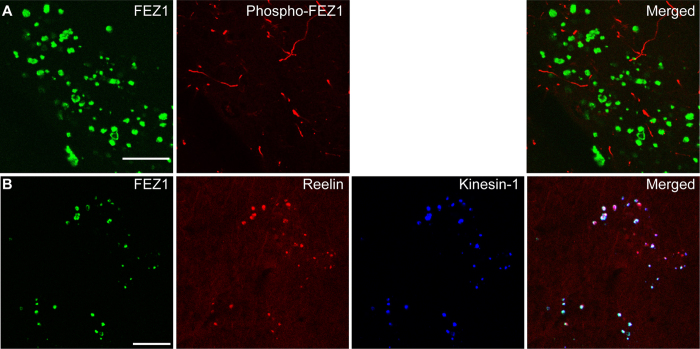
FEZ1-Kinesin-1 aggregates contain unphosphorylated FEZ1 and Reelin. (**A**) FEZ1-Kinesin-1 aggregates contain unphosphorylated FEZ1. Double staining of brain sections from 2 year-old 3XTg-AD mice using antibodies directed against FEZ1 and phosphorylated FEZ1 reveals the absence of phosphorylated FEZ1 in FEZ1-Kinesin-1 aggregates. (**B**) FEZ1-Kinesin-1 aggregates colocalise with Reelin aggregates. Triple staining of brain sections from 2 year-old 3XTg-AD mice using antibodies directed against FEZ1, Reelin and Kinesin-1 reveals the presence of all 3 proteins in FEZ1-Kinesin-1 aggregates. Scale bars, 20 μm.

Our previous data indicated that loss of FEZ1 function by preventing phosphorylation at S58 leads to the appearance of axonal FEZ1 aggregates. The appearance of similar aggregates in 3XTg-AD mice strongly suggests that they may also arise from functionally impaired FEZ1 caused by loss of phosphorylation. We tested for the presence of FEZ1 S58 phosphorylation in these aggregates using the S58 phospho-specific antibody. In the brains of 2 year-old 3XTg-AD mice, the antibody stains a subset of neuronal processes. Strikingly, immunolabelling with the antibody is not observed at sites of FEZ1 aggregation, demonstrating that they do not contain detectable amounts of S58-phosphorylated FEZ1 (Fig. 7A).

Noteworthy, these aggregates do not contain Aβ or hyperphosphorylated Tau, suggesting that they are not related to Aβ plaques or neurofibrillary tangles (See Supplementary Fig. S6). Instead, the timing of appearance and the distribution pattern of FEZ1 aggregates in the hippocampus appear to share some similarity to the previously reported Reelin aggregates^48^. Reelin aggregates are extracellular accumulations found in the brains of both aging and 3XTg-AD mice and originate via the extrusion of misfolded and aggregated proteins from dystrophic neurites^49^. Importantly, Reelin aggregates are postulated to originate from cargo stranded as consequence of compromised axonal transport^50^. Like FEZ1-Kinesin-1 aggregates, Reelin aggregates increase with age in both wild type and 3XTg-AD mice. Both forms of aggregate are found predominantly in hippocampal areas but are not observed in the cortical brain regions. To examine the relationship between these aggregates, we performed triple immunofluorescence labelling of FEZ1, Reelin and Kinesin-1. Colocalisation of all 3 proteins in FEZ1 aggregates indicate a possible link between FEZ1 and Reelin aggregates and support that FEZ1 aggregates are likely also to originate intracellularly and subsequently accumulate extracellularly with deterioration of the neuronal processes (Fig. 7B).

## Discussion

Despite their importance in Kinesin-based intracellular transport, there have been limited studies characterizing the cargo spectrum and mechanisms of regulation of motor adapters. In this study, we report that FEZ1, a Kinesin-1 adapter involved in neuronal development and axonal transport, is associated with cargoes enriched for presynaptic functions and is regulated by MARK/Par-1, a kinase implicated in the pathogenesis of Alzheimer’s disease.

The cargo spectra for only a few Kinesin-1 adapters involved in axonal transport have been reported. Calsyntenin was shown to associate with vesicles originating from early and recycling endosomal pathways^51^. However, neither calsyntenin nor markers associated with calsyntenin-1-associated vesicle pools (syntaxin-13; Rabs 4, 5 and 11) were detected in FEZ1-Kinesin-1 vesicles. This indicates that the two adapters are unlikely to bind identical cargoes or be co-transported by Kinesin-1. Nevertheless, calsyntenin-vesicles do share a limited number of cargoes that are also associated with FEZ1-Kinesin-1 vesicles.

Interestingly, JIP3 (but not JIP1 or JIP2) was identified in our data. Simultaneous binding of JIP1 and JIP3 was previously thought to fully activate Kinesin-1 but a more recent study revealed that JIP3 alone can also activate the motor albeit less effectively^5^,^7^. Thus, full activation of the motor would require additional binding of other proteins. Our finding that JIP3 and FEZ1 are both present in FEZ1-Kinesin-1 vesicles suggests that concurrent binding of both proteins to Kinesin-1 may fulfil this function as was previously shown for JIP1 and FEZ1^8^.

JIP3 is associated with two morphologically distinct vesicle populations: an anterograde- and a retrograde-migrating pool^6^. The former pool of vesicles appears to carry proteins involved in axonal development whereas the latter pool probably represents retrograde moving signalling cargoes. This is consistent with JIP3’s involvement in axonal growth as well as in retrograde signalling trafficking during conditions of neuronal injury^10^,^52^. FEZ1 also functions during neuritogenesis^15^,^16^. Furthermore, several proteins involved in axonal development are associated with FEZ1-Kinesin-1 vesicles (e.g. CRMP1 and GAP43). Nevertheless, only 29% and 8% of the proteins identified here overlap with retrograde and anterograde Syd vesicles, respectively. Thus, FEZ1-Kinesin-1 vesicles are unlikely to be related to the two previously characterized JIP3 pools.

Significantly, cargoes associated with FEZ1-Kinesin-1 vesicles are enriched for proteins involved in presynaptic function. Active zone proteins such as Piccolo and Bassoon which were previously reported to be transported using the Kinesin-1 adapter syntabulin were also detected in our immunoisolated samples^31^. However, syntabulin was not identified in these samples, suggesting that, while both adapters are involved in transporting presynaptic proteins, FEZ1-Kinesin-1 vesicles may represent a distinct group of cargo. Noteworthy, dramatic reduction and disruption of presynaptic sites are observed in both *unc-76* and *par-1* mutants. This is consistent with previous findings reporting that UNC-76 together with UNC-69 is important for proper presynaptic development in *C. elegans*^53^. These results are supportive that FEZ1 plays an important role not only in presynaptic protein delivery but also in the establishment and maintenance of synapses.

Impairment of Kinesin-1 mediated axonal transport is observed in a number of neurodegenerative disorders, including Alzheimer’s disease^12^,^43^,^44^. However, the consequences of this defect on the delivery of synaptic proteins have not yet been directly addressed. Loss of synapses is an early and consistent feature of Alzheimer’s disease^54^. Interestingly, FEZ1 binding to Kinesin-1 did not significantly affect the speed or processivity of the motor as assessed using *in vitro* motility assays. Instead, the number of vesicles observed to move was significantly increased^8^. This would suggest that, when binding of FEZ1 to Kinesin-1 is reduced (e.g. when FEZ1 becomes unphosphorylated), an increase in the number of stalled cargo-containing vesicles could appear over time and eventually lead to aggregate formation along axons. This would also diminish protein delivery to synapses thereby contributing to synaptic dysfunction. FEZ1 is a substrate of MARK/PAR-1. Defects in axonal transport and presynaptic organization in *par-1* mutants supports that abnormal kinase signalling potentially contributes to a FEZ1-dependent impaired Kinesin-1 transport. While we cannot completely exclude the possibility that FEZ1 aggregates could correspond to dystrophic neurites, Reelin-containing aggregates are known to originate intracellularly as a result of impaired axonal transport^48^,^50^. Thus, the presence of Reelin in FEZ1 aggregates supports the notion that these aggregates are likely to arise via this mechanism.

Nevertheless, how does one reconcile tau hyperphosphorylation with our observation that FEZ1 becomes under-phosphorylated under the same circumstances? Although we currently do not completely understand the mechanistic details of this phenomenon, increased physical association between tau and activated MARKs and colocalisation of these proteins have been reported in Alzheimer’s disease^55^,^56^. We speculate that tau sequestration of activated MARKs reduces their availability to phosphorylate FEZ1, resulting in an unphosphorylated pool of FEZ1 impaired for axonal transport that gradually aggregates. In future studies, it will be important to ascertain how altered MARK signalling is involved in FEZ1 aggregation during AD pathogenesis.

## Materials and Methods

### Constructs and antibodies

The open reading frames encoding human MARKs 1–3 were amplified by PCR. cDNA sequences encoding the inactive kinase mutants were generated by PCR mutagenesis. Inserts were then cloned into the GATEWAY entry vector pENTR/D-TOPO. Eukaryotic plasmids expressing GFP-tagged wild type or mutant kinases were obtained by shuttling the inserts from their respective entry vectors to pcDNA6.2/N-EmGFP-DEST. Plasmids expressing V5-tagged wild type or S58A FEZ1 were previously described^19^. *unc-64*, *unc-76* and *snb-1* coding sequences were amplified from a *C. elegans* cDNA library by PCR, subcloned into pDonr201 vector (Invitrogen) and subsequently cloned into corresponding Gateway (GW) destination vectors. The Rab3::GFP-GW plasmid has been previously described^19^. Rab3::VN155-GW, Rab3::VC155-GW and Rab3::Cerulean-GW vectors were derived from Rab::3-GFP-GW by replacing GFP with VN155, VC155 or Cerulean, respectively, between the *Asc*I and *Nhe*I restriction sites.

A list of antibodies used in this study can be found in Supplementary Table 1. The murine monoclonal against phosphorylated S58 of FEZ1 was generated following the standard protocol from Synaptic Systems (Göttingen, Germany). Briefly, three 8- to 10-week-old female BALB/c mice were immunized over a period of 17 days with an FEZ1 phospho-peptide (EIISFKpSMEDL) coupled to KLH. Cells from knee lymph nodes were fused with the mouse myeloma cell line P3 × 63Ag.653 (ATCC CRL-1580). Cell culture supernatants obtained from individual clones were then screened using an enzyme-linked immunosorbent assay (ELISA) and immunoblot assays to ascertain their specificity towards phospho-58. The final hybridoma used in this study was cloned two times by limiting dilution. The monoclonal antibody produced from this clone was determined to be of the IgG2a subclass.

### Cell culture and transfection

Human embryonic kidney (HEK) 293 and HeLa cells were maintained in Dulbecco’s modified Eagle’s medium supplemented with 10% fetal calf serum. For transfection using Lipofectamine reagent (Invitrogen), cells were seeded at a density of ~90% and transfected with various plasmids one day after plating following the recommended protocol.

### Immunoisolation

Rat brain cortices were homogenized in ice-cold sucrose buffer (5 mM Hepes, pH 7.4, 320 mM sucrose) and centrifuged for 2 min at 2,988 × *g*. The ensuing supernatant was re-centrifuged for 12 min at 14,461 × *g*. One milligram of protein from the resulting supernatant (S2) was then mixed with an equal volume of 2× PBS/BSA (0.6% BSA, 5.4 mM KCl, 3 mM KH_2_PO_4_, 274 mM NaCl, 16 mM Na_2_HPO_4_, pH 7.3) and used as starting material for immunoisolation of FEZ1 or Kinesin-1 transport vesicles. Antibodies recognizing FEZ1 or Kinesin-1 were added to the mixture and incubated overnight at 4 °C. To isolate the immune-complexes, Dynabeads Protein A (Invitrogen) was added to the mixture and incubation was continued for an additional hour. After 3 washes with PBS plus 0.3% BSA, the complexes were resuspended in 2× LDS buffer (Invitrogen) and analysed by mass spectrometry. The samples were then resolved using a 4–12% gradient SDS-PAGE gel and immunoblotted to visualize the protein bands.

### Mass spectrometry, sample preparation and data analysis

Eluted proteins were separated on 4–12% gradient SDS-PAGE gels (Invitrogen) and stained with Colloidal Coomassie Blue. Each gel lane was cut into 23 equal gel slices and proteins therein were in-gel digested with trypsin as described^57^. Tryptic peptides from each gel slice were analysed as described^58^ by nanoflow HPLC (Agilent 1100, Agilent Technologies) coupled to nanoelectrospray LTQ-Orbitrap XL mass spectrometer (Thermo Fischer Scientific). Raw MS data files were analysed by MaxQuant (version 1.3.0.5)^22^ and Andromeda^59^ using the UniProt rat protein database (version 05.13) and with selected “label-free protein quantification” (LFQ). Results from MaxQuant were further processed using Perseus (version 1.3.0.4, www.perseus-framework.org). Contaminant and reverse entries were filtered and all LFQ intensities were logarithmised. Missing values were imputed with random numbers using normal distribution (width = 0.3, down shift = 1.8) to simulate low abundance intensity values^21^. A modified *t* test (250 permutations, FDR = 0.01, S_0_ = 2)^60^,^61^ was applied to identify proteins significantly enriched within each IP experiment using four biological replicates and compared to identical control IP.

### Functional Analysis

The Ingenuity Pathway Analysis software (build version 21901358) was used to perform functional analysis on the list of common proteins identified in FEZ1 and Kinesin-1 immunoisolated vesicles to identify biological functions significantly enriched within each data set. Only clusters having a *p* value < 0.01 and containing at least 10 proteins were considered for the analysis. The heat maps were generated using Microsoft Excel.

### *In vitro* kinase screening and co-immunoprecipitation assays

The ability of 190 different serine/threonine kinases to phosphorylate a FEZ1 peptide containing S58 (SEIISFKSMEDLVNEF) was evaluated using a KinaseFinder assay (ProQinase GmbH) according to the manufacturer’s protocol. The list of kinases used can be found in Supplementary Table 4. Validation of the ability of MARKs to specifically recognize S58 was determined using a KinaseFinder Verification assay (ProQinase GmbH). Briefly, dose-dependent responses of MARKs to 3 different concentrations of the FEZ1 peptide were determined. In addition, we also determined the activities of each MARK against a control peptide in which S58 was mutated to an alanine (SEIISFKAMEDLVNEF).

For co-immunoprecipitation assays, cells were lysed one day after transfection with ice-cold HNE buffer (150 mM NaCl, 1 mM EDTA, 1% Triton X-100, 50 mM Hepes, pH 7.2) containing Complete EDTA-free protease inhibitor mixture (Roche). Cell lysates were cleared by centrifugation at 10,000 × *g* for 10 min at 4 °C and the resultant supernatant was incubated with anti-GFP antibodies for 3 h. Thirty microliters of protein G-Sepharose were then added to the mixture and incubated continued for an additional hour. Next, immunoprecipitates were washed 4× with HNE buffer. Proteins were subsequently eluted with 2 × LDS buffer separated using electrophoresis and analysed by immunoblotting.

### C. strains and generation of transgenic animals

*C. elegans* strains were cultured at 20 °C as described before^62^. The wild type N2 and mutant strains *unc-18 (md299), unc-79 (e911), par-1 (zu310)* and *par-1 (ok2001)* were obtained from the *C. elegans* Genetic Center (CGC, University of Minnesota) which is funded by the NIH Office of Research Infrastructure Program (P40 OD010440).

Transgenic strains were generated by microinjection^63^ of the plasmids (1) *prab3::gfp-unc-64* and *prab-3::cerulean-unc-76* (10 ng/μl each); (2) *prab3::VC155-unc-64* and *prab3::VN155-unc-76* (30 ng/μl each) and (3) *prab3::gfp-snb-1* (5 ng/μl). In all cases the markers pRF4 (*rol-6 (su1006*)) (100 ng/μl) and *odr-1-RFP* (50 ng/μl) were co-injected. The wild type transgenic animals were crossed with the respective mutants using classical genetic approaches and the progeny was genotyped by PCR.

For imaging, live adult animals were immobilized with 5 mM Levamisole in M9 buffer on 4% agarose pads at 20 °C. Confocal images of Cerulean-UNC-76 and GFP-UNC-64 were acquired using an inverted confocal microscope (Leica SP5X) using a 63x/NA1.4 objective. The BiFC signal was acquired using an Axiovert 200 M microscope equipped with a 60x/NA1.4 objective, a spinning disk confocal unit (Yokagawa CSU10) and an Andor™ iXon EM-CCD camera. Average intensity projections of four single plane images were performed using ImageJ software.

### Immunofluorescence labelling of brain sections

All animal experiments were performed according to the European Communities Council Directive (86/609/EEC). The experiments with mice were approved by local authorities (Regierungspräsidium Leipzig, Germany; reference number T40/13). All staining procedures were applied to free-floating 30 μm-thick coronal frozen sections containing hippocampi from brains of 3XTg-AD transgenic mice with age-dependent β-amyloidosis and tau hyperphosphorylation^20^ or age-matched wild-type mice perfusion-fixed with 4% paraformaldehyde. For single labelling of FEZ1, sections were extensively rinsed with 0.1 M Tris-buffered saline (TBS) and blocked with 5% normal donkey serum in TBS containing 0.3% Triton X-100 (NDS-TBS-T) for 1 h. The sections were then incubated overnight with rabbit anti-FEZ1 (1:200 in NDS-TBS-T) and subsequently exposed to donkey anti-rabbit IgG conjugated to AlexaFluor 488 (Jackson ImmunoResearch, 20 μg/ml TBS containing 2% bovine serum albumin) for 1 h. Sections for double and triple fluorescence staining were similarly processed using primary and secondary antibodies as listed in Supplementary Table 1. After all staining procedures, rinsed sections were treated with Sudan Black B to quench autofluorescence^64^. Finally, the sections were mounted using glycerol/gelatine (Sigma). Images were acquired using a LSM 710 (Zeiss) or a Leica SP2 confocal microscope.

## Additional Information

**How to cite this article**: Butkevich, E. *et al.* Phosphorylation of FEZ1 by Microtubule Affinity Regulating Kinases regulates its function in presynaptic protein trafficking. *Sci. Rep.* **6**, 26965; doi: 10.1038/srep26965 (2016).

## Supplementary Material

Supplementary Information
