# Supplementary material for: Phosphorylation of FEZ1 by Microtubule Affinity Regulating Kinases regulates its function in presynaptic protein trafficking
Source: Sci Rep. 2016 Jun 1;6:26965. doi: 10.1038/srep26965 (PMC4887895; doi:10.1038/srep26965)
Supplement: Supplementary Information [file srep26965-s1.pdf]

Phosphorylation of FEZ1 by Microtubule Affinity Regulating Kinases regulates its function in presynaptic protein trafficking

Eugenia Butkevich, Wolfgang Härtig, Miroslav Nikolov, Christian Erck, Jens Grosche, Henning Urlaub, Christoph F. Schmidt, Dieter R. Klopfenstein, John Jia En Chua

Supplemental Procedures

Table S1. List of antibodies used in this study.

Antigen	Host species of primary antibody	Dilution/ Concentration	Source	Fluorophores of secondary antibodies used
Bassoon	Guinea Pig	1:300, 1:200	Synaptic Systems, Göttingen, Germany	AlexaFluor (AF) 488, AF647
FEZ1	Rabbit	1:200	Chua et al., 2012	Cy2, AF488, AF647
FEZ1 phospho S58	Mouse	1:200	This study; Synaptic Systems, Göttingen, Germany	Cy3
Kinesin-1	Mouse (IgM, clone KN-03)	1:100	Abcam, Cambridge, UK	Cy3
Munc13-1	Rabbit	1:100	Synaptic Systems, Göttingen, Germany	Cy2
Munc18	Rabbit	1:100	Synaptic Systems, Göttingen, Germany	Cy2
Munc18	Mouse (clone 131.1)	1:200	Synaptic Systems, Göttingen, Germany	Cy3
NeuN	Mouse (biotinylated, clone A60)	1:100	Millipore, Billerica, MA, USA	Cy3
Piccolo	Rabbit	1:300	Synaptic Systems, Göttingen, Germany	AF488
Reelin	Mouse (Cy3-tagged, clone G10)	20 μg/ml	Millipore; fluorochromated by WH	Cy3
GFP	Rabbit	1:20000	Synaptic Systems, Göttingen, Germany	HRP
FLAG	Mouse	1:2000	Stratagene, La Jolla, CA, USA	HRP
V5	Mouse	1:100	Santa Cruz Biotechnology, Heidelberg, Germany	HRP

Supplemental Figures

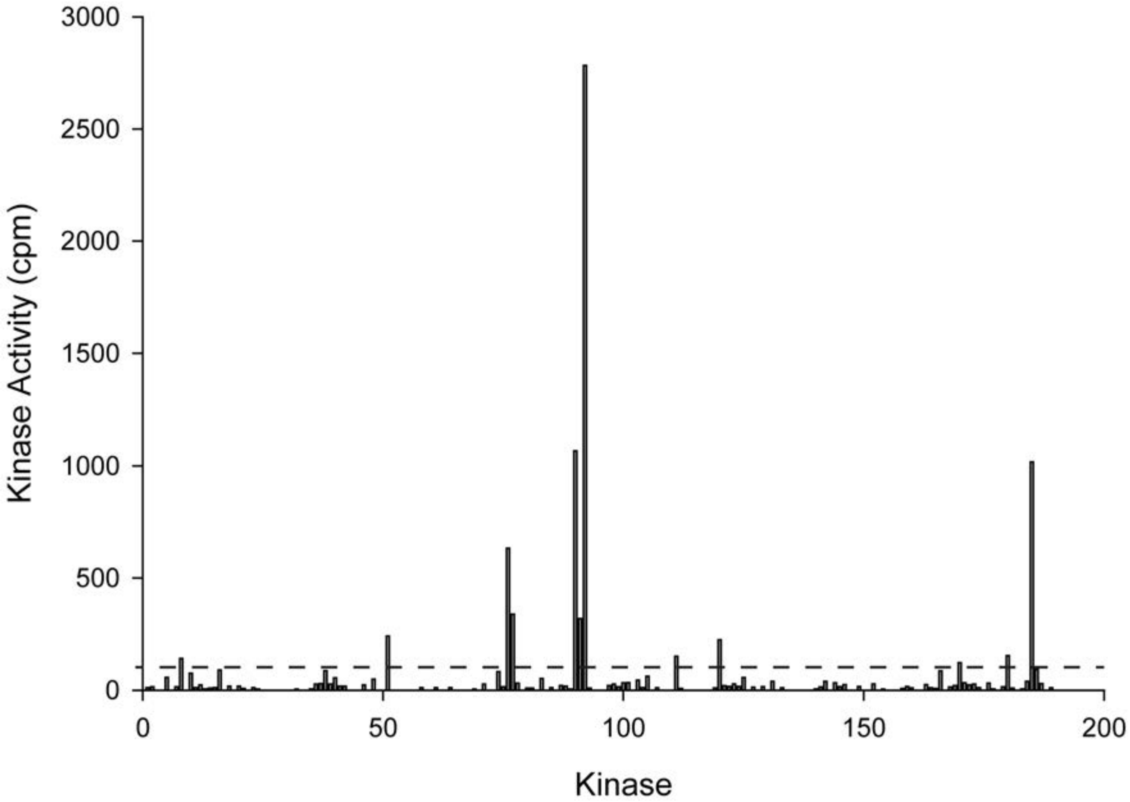

Supplementary Figure S1. Results of the *in vitro* serine/threonine kinase screen. One hundred and ninety serine/threonine kinases were screened against the wild type FEZ1 peptide (SEIISFKSMEDLVNEF) as described in the main text. Dotted line indicates the threshold above which kinases are considered to exhibit significant activity against the peptide.

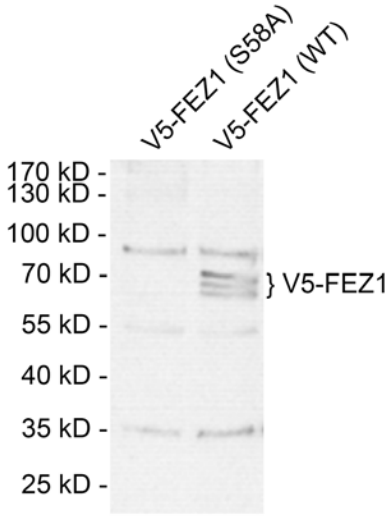

Supplementary Figure S2. Specificity of monoclonal phospho-antibody against phosphorylation at FEZ1 S58. Wild type or the FEZ1(S58A) phospho-mutant bearing V5 epitope tags were transiently expressed in HEK 293 cells. Cell lysates were then immunoblotted using the monoclonal phospho-antibody targeting phosphorylated S58. The antibody recognizes only wild type FEZ1 phosphorylated at S58 but not when the site is replaced by an alanine residue.

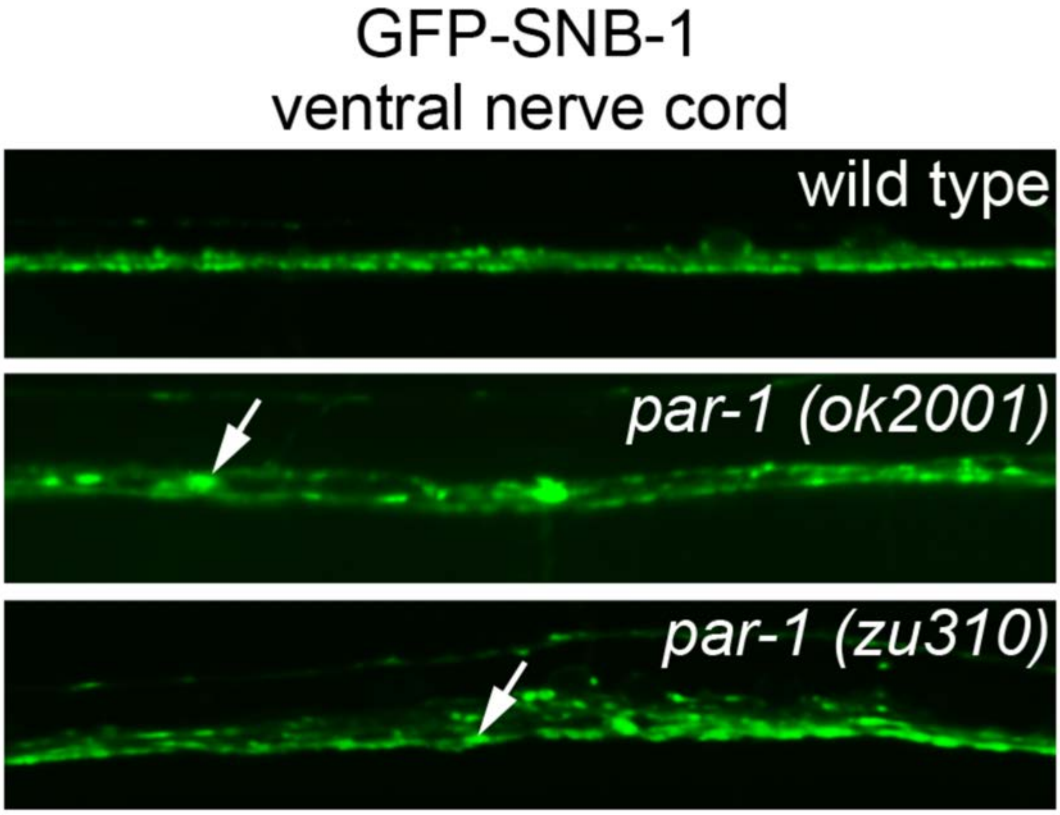

Supplementary Figure S3. Defects in presynaptic specializations are present the ventral nerve cords of *par-1* mutants. As for the DNC, GFP-SNB-1 punctae corresponding to presynaptic sites are regularly spaced in the VNC of wild type worms. In both *par-1* mutants, GFP-SNB-1 punctae either become dispersed or exhibit abnormal aggregation (arrows).

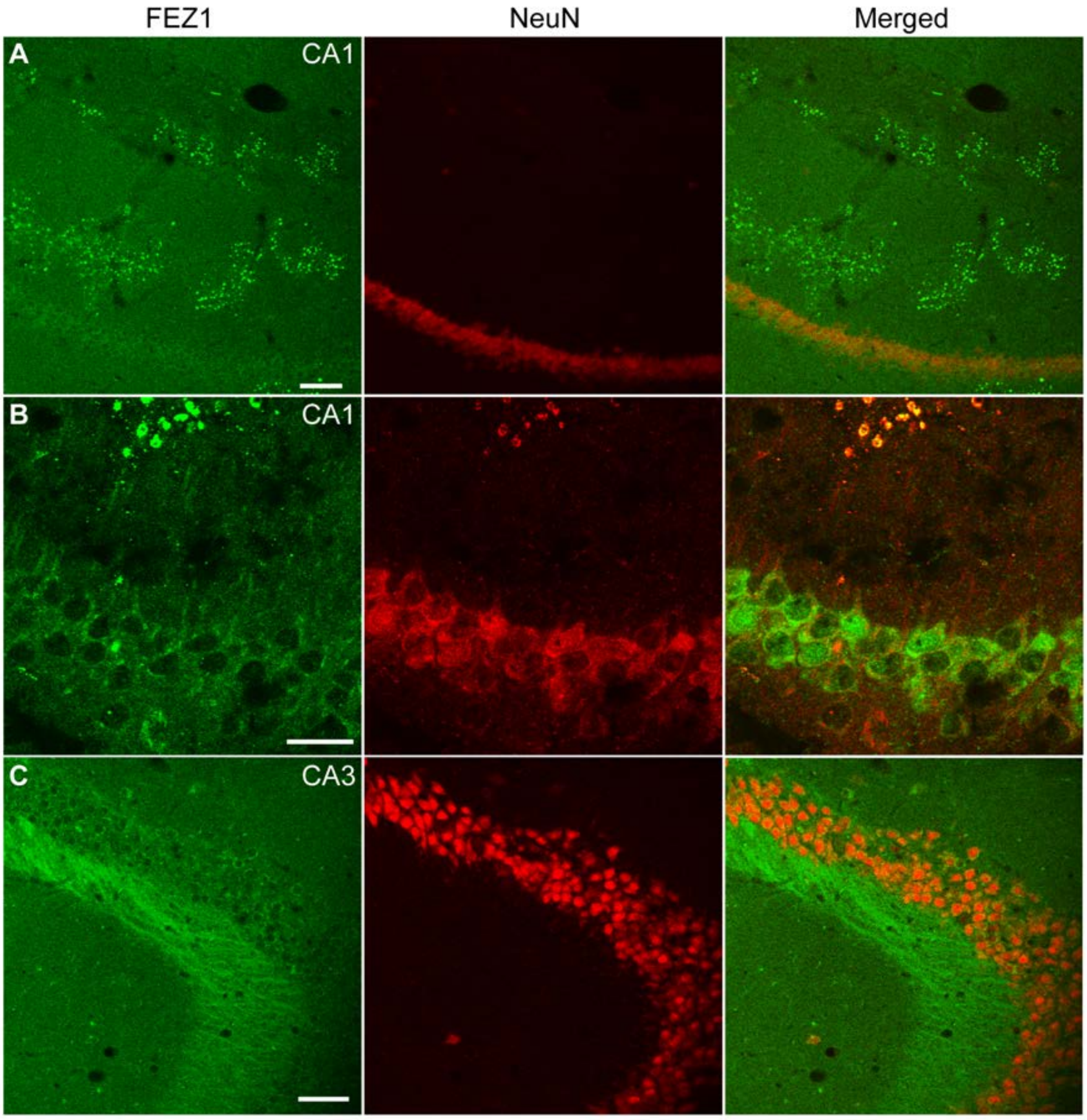

Supplementary Figure S4. Distribution of FEZ1 in the hippocampus of a 2 year-old wild type mouse. (A and B) Higher magnification views of the CA1 region showing FEZ1 aggregation in these regions. FEZ1 expression occurs mainly in neurons as revealed by the presence of FEZ1 in NeuN-immunopositive cell bodies. Scale bars, 50 μm (A) and 20 μm (B). (C) No aggregation of FEZ1 is observed in CA3 mossy fiber projections. Scale bar, 50 μm.

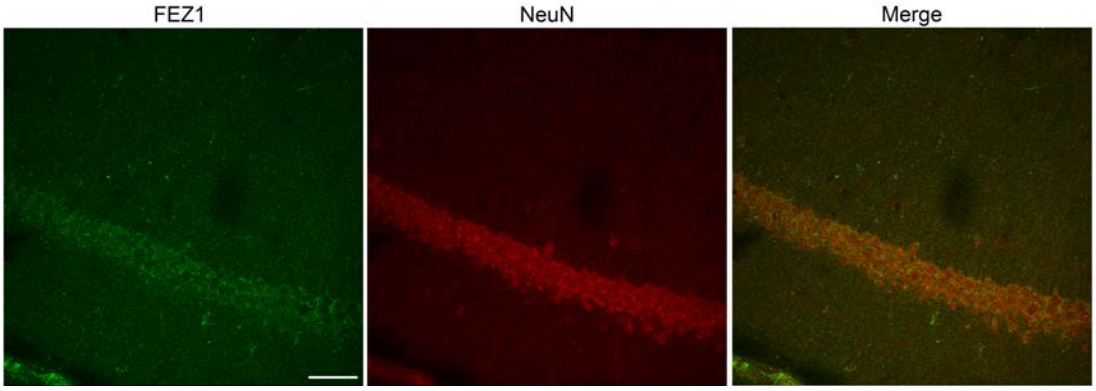

Supplementary Figure S5. Lack of aggregation in the hippocampus of a 3 month-old 3x-Tg-AD type mouse. Higher magnification views of the CA region showing lack of FEZ1 aggregation in these regions. Scale bar, 50 μm.

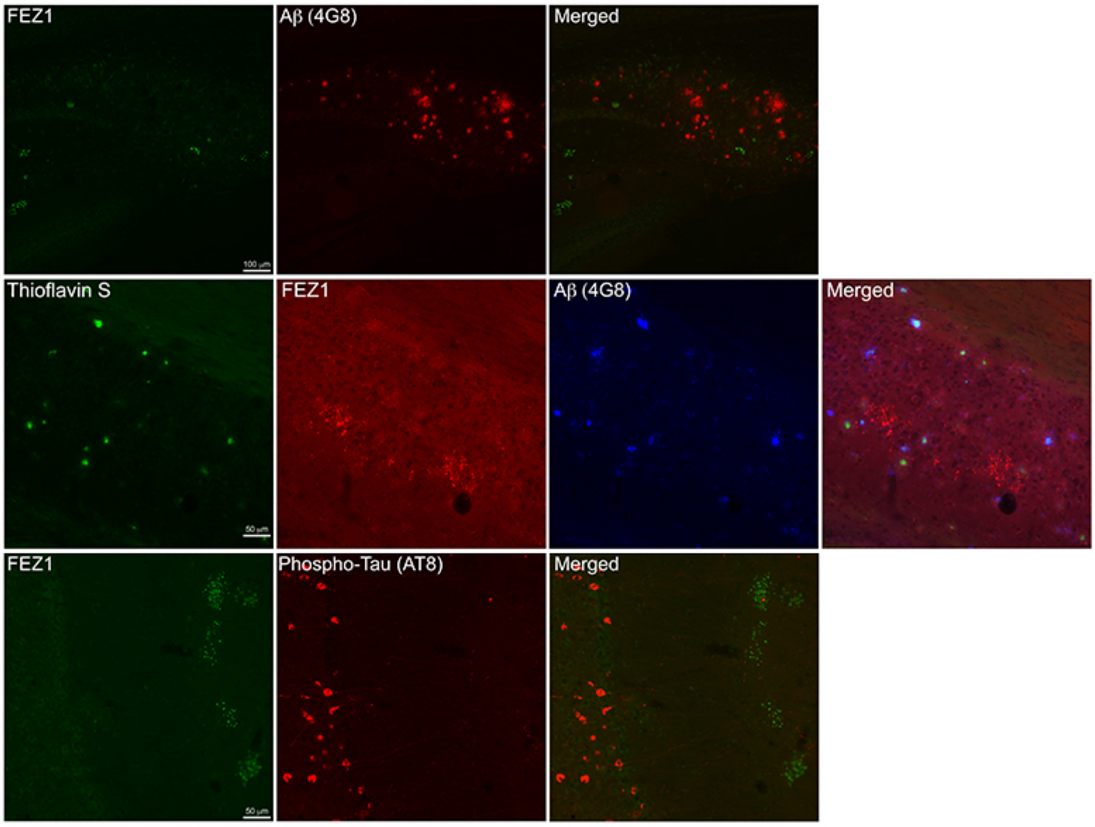

Supplementary Figure S6. FEZ1 aggregates do not contain Aβ or hyperphosphorylated tau. Brain sections from 2 year-old 3x-Tg-AD mice were stained for FEZ1 and Aβ (top panel); FEZ1, Aβ and Thioflavin S (middle panel); FEZ1 and phospho-tau (bottom panel). Images were obtained using a Keyence fluorescence microscope (BZ-9000).

Supplementary Tables

Supplementary Table S2. List of proteins significantly enriched in both immunoisolated FEZ1 and Kinesin-1 containing vesicles by quantitative mass spectrometry.

Supplementary Table S3. List of kinases tested for activity against FEZ1 serine-58.

Supplementary Table 2. List of proteins significantly enriched in both immunoisolated FEZ1 and Kinesin-1 containing vesicles by quantitative mass spectrometry

Group	Gene Symbol	Gene Name	log LFQ intensity IP1 Control	log LFQ intensity IP2 Control	log LFQ intensity IP3 Control	log LFQ intensity IP4 Control	log LFQ intensity IP1 Kinesin-1	log LFQ intensity IP2 Kinesin-1	log LFQ intensity IP3 Kinesin-1	log LFQ intensity IP4 Kinesin-1	log LFQ intensity IP1 FEZ1	log LFQ intensity IP2 FEZ1	log LFQ intensity IP3 FEZ1	log LFQ intensity IP4 FEZ1	t-test Significant Kinesin-1 IP	t-test Significant FEZ1 IP	t-test Significant Kinesin-1 and FEZ1 IP	-Log t-test p value Kinesin-1 IP	t-test Difference Kinesin-1 IP	-Log t-test p value FEZ1 IP	t-test Difference FEZ1 IP	Majority UniProt protein IDs
Active Zone	Bsn	bassoon (presynaptic cytomatrix protein	22.1156	24.62845	25.24985	23.95961	32.18766	31.22969	32.2413	31.75298	32.38623	31.68407	32.83915	32.14349	+	+	+	4.466861514	7.864530087	4.586984344	8.274856567	G3V984;O88778
Active Zone	Cask	calcium/calmodulin-dependent serine p	21.58528	20.66983	22.66562	22.22122	28.71355	27.73712	28.6065	28.68264	29.06551	27.68726	28.61207	28.77718	+	+	+	4.992543594	6.649465561	4.863766027	6.750015736	Q62915
Active Zone	Erc2	ELKS/RAB6-interacting/CAST family mer	22.63988	24.19642	22.40201	22.8025	27.35128	26.59548	27.23863	26.80907	28.02263	26.75366	27.49213	25.99828	+	+	+	3.987574966	3.988414288	3.299231724	4.05647707	2
Active Zone	Lin7a	lin-7 homolog A (C. elegans)	22.94859	23.99235	23.18138	27.72826	28.98242	27.89452	28.20415	28.63457	28.73918	28.08527	27.71017	28.90308	+	+	+	1.886288886	3.966273308	1.839159059	3.896782875	2;M0R7K1
Active Zone	Lin7c	lin-7 homolog C (C. elegans)	22.71503	21.58083	21.90885	22.44874	27.30034	26.92988	21.58278	26.88693	26.44517	23.75237	22.77846	26.76129	+	+	+	1.346590242	3.51161623	1.460308306	2.770960808	Q792I0
Active Zone	Pclo	piccolo (presynaptic cytomatrix protein)	23.38578	23.2727	21.54833	21.66892	27.58176	27.03612	27.1379	27.053	28.3333	27.6095	27.94765	28.09522	+	+	+	4.033154763	4.733257294	4.387051668	5.52748251	D3Z9C7;F1M7V4
Active Zone	Pclo	piccolo (presynaptic cytomatrix protein)	21.08066	21.8596	23.41148	21.19017	30.7939	30.44352	31.1566	30.4451	31.98797	31.37394	31.60731	31.49109	+	+	+	5.367711578	8.824300289	5.663601073	9.729598999	Q9JKS6;Q9JKS6-3;Q9JKS6-2
Active Zone	Ppfia3	protein tyrosine phosphatase, receptor	22.03802	22.55579	23.05197	25.01631	27.72338	27.82984	28.34832	27.86313	28.44691	28.6619	28.33807	28.40027	+	+	+	3.43218435	4.775646687	3.719050464	5.296267509	F1LSE6;Q91Z79
Active Zone	Unc13a	unc-13 homolog A (C. elegans)	22.70497	22.16095	22.49215	21.96703	27.86136	27.32809	28.42178	28.11048	27.54429	27.62575	28.39489	28.1155	+	+	+	5.955961763	5.599152565	6.167649434	5.588833809	Q62768;Q62768-3;Q62768-2
Adhesion and Cell Surface	Alcam	hypothetical protein LOC100133690; ac	21.02549	22.34282	22.25985	22.8341	29.87282	29.31834	30.21674	30.16015	30.20163	29.58765	30.30269	30.17902	+	+	+	5.698258922	7.776445866	5.867552031	7.9521842	O35112
Adhesion and Cell Surface	Bcan	brevican	22.62606	22.98462	21.65729	22.31517	26.62157	26.35028	27.01755	25.67374	28.71224	27.97684	28.54933	28.73641	+	+	+	4.255333503	4.019998074	5.76939344	6.097919941	G3V8G4;P55068;P55068-2
Adhesion and Cell Surface	Cadm3	cell adhesion molecule 3	21.85163	23.11056	22.66637	23.24605	30.0879	29.14136	30.42202	30.09245	30.37938	29.61915	29.81472	30.06072	+	+	+	5.613717413	7.217279911	6.05020628	7.249838352	Q1WIM3
Adhesion and Cell Surface	Cd81	CD81 molecule	23.22871	20.70872	22.12811	20.98279	29.57937	28.53249	29.74005	29.89879	29.30069	29.13568	29.97992	30.03026	+	+	+	4.633415384	7.675590038	4.821009608	7.849550247	Q6P9V1;Q62745
Adhesion and Cell Surface	Cdh13	cadherin 13, H-cadherin (heart)	21.56188	22.94514	22.06806	22.97054	28.9579	28.87531	28.79984	28.97937	29.00935	29.26264	29.3226	29.20065	+	+	+	5.823319897	6.516699791	5.906737618	6.812402725	F1M7X3
Adhesion and Cell Surface	Cdh2	cadherin 2, type 1, N-cadherin (neurona	22.04147	21.95527	24.10734	22.00516	25.94829	27.96014	28.1398	28.13232	27.87403	28.38546	28.17783	27.95942	+	+	+	3.266537267	5.017828941	4.317876015	5.571875095	G3V803;Q9Z1Y3
Adhesion and Cell Surface	Cntn1	contactin 1	26.046	28.15919	27.84739	27.56391	32.3785	32.1575	32.43233	32.54979	32.50045	32.32595	32.38193	32.43864	+	+	+	4.347564222	4.975410938	4.393395091	5.007623196	Q63198
Adhesion and Cell Surface	Cntn2	contactin 2 (axonal)	22.86033	21.86384	23.11301	23.05573	26.50279	26.08111	27.04937	27.11118	26.82457	26.8947	26.73117	27.26472	+	+	+	4.341822258	3.962884903	4.968927245	4.205562592	G3V758;P22063
Adhesion and Cell Surface	Cntnap1	contactin associated protein 1	22.01125	21.7416	22.77385	23.94756	29.23822	29.81164	29.60327	29.32642	29.32896	30.10212	30.54894	29.61716	+	+	+	4.981033494	6.876325607	4.883337331	7.280734539	P97846
Adhesion and Cell Surface	Cspg5	chondroitin sulfate proteoglycan 5 (neu	21.50877	23.02446	22.54447	22.32579	27.88145	26.352	26.10827	26.39216	27.22426	26.22946	26.12023	27.33858	+	+	+	3.821231938	4.332598209	4.164727131	4.377256393	Q9ERQ6;F1M4R7
Adhesion and Cell Surface	Ctnna1	catenin (cadherin-associated protein), a	21.43846	22.24694	23.33223	24.20655	26.81653	26.42145	26.80735	27.30408	27.05982	27.06538	27.16077	27.12149	+	+	+	3.151292529	4.031307697	3.397215895	4.295820236	Q5U302
Adhesion and Cell Surface	Ctnna2	catenin (cadherin-associated protein), a	23.28198	22.4153	22.5925	23.50093	29.52657	28.87209	29.39773	28.92782	29.66997	29.12726	29.66284	29.10545	+	+	+	6.00750923	6.233371735	6.118956972	6.443699837	D4A6H8
Adhesion and Cell Surface	Ctnnd2	catenin (cadherin-associated protein), d	23.15336	23.24734	22.56259	21.76138	28.20918	26.03997	27.63464	27.08881	28.17793	27.11942	27.1849	27.76376	+	+	+	3.673333002	4.561983109	4.585139161	4.88033247	F1M787
Adhesion and Cell Surface	Epdr1	ependymin related protein 1 (zebrafish)	22.28483	23.07882	20.37467	23.58932	25.92134	26.07292	25.73138	25.98553	26.05666	25.98639	25.06883	25.65349	+	+	+	2.640794851	3.595880508	2.403516465	3.35943079	Q5XII0
Adhesion and Cell Surface	Gja1	gap junction protein, alpha 1, 43kDa	23.11467	21.46301	22.20459	21.78611	29.59071	29.09519	29.89183	29.80229	29.92139	29.64219	29.79792	30.04869	+	+	+	5.812776445	7.452911377	6.111156562	7.710453033	P08050
Adhesion and Cell Surface	Hapln1	hyaluronan and proteoglycan link prote	22.02906	23.49074	21.71746	22.24238	28.23316	27.5786	27.85596	27.5679	28.51461	27.99313	27.61484	28.26566	+	+	+	4.889839224	5.438995361	4.931896808	5.727152824	P03994;P03994-2
Adhesion and Cell Surface	Icam5	intercellular adhesion molecule 5, telen	22.49953	22.4879	22.59241	21.80596	28.96166	28.37352	29.72684	29.37111	29.55208	28.90191	28.98357	29.63625	+	+	+	5.959199425	6.761831284	6.706440416	6.922000408	D4A435
Adhesion and Cell Surface	Igsf21	immunoglobin superfamily, member 21	22.03345	23.02243	22.12823	22.55138	26.62422	25.60128	25.7029	25.74983	26.45447	25.44944	24.93161	25.98444	+	+	+	4.39156799	3.485682964	3.746341265	3.271119118	M0RAS4
Adhesion and Cell Surface	Itfg1	integrin alpha FG-GAP repeat containing	21.80721	21.95894	22.17605	23.08293	26.23085	26.19545	27.10149	26.93787	26.64787	26.61358	26.43371	26.64623	+	+	+	4.645535974	4.36013031	5.242368138	4.329062939	Q5U355;Q8R4E1
Adhesion and Cell Surface	Itgav	integrin, alpha V (vitronectin receptor, a	22.85404	21.87647	20.77722	21.52626	25.68635	26.22689	27.00045	26.61625	27.09748	26.5589	27.27711	26.70543	+	+	+	3.974322073	4.623986721	4.505793846	5.151232243	F1LZX9
Adhesion and Cell Surface	Itih3	inter-alpha (globulin) inhibitor H3	22.83709	23.25243	20.46169	22.2373	26.82189	28.14822	28.68177	27.36143	26.66519	28.70955	28.48669	28.97123	+	+	+	3.538607587	5.556202412	3.518299387	6.011038303	Q63416;D3ZBS2
Adhesion and Cell Surface	L1cam	L1 cell adhesion molecule	22.89668	22.66545	22.42915	22.00478	29.9421	29.15369	30.55014	30.66008	30.21257	29.28685	30.353	30.4517	+	+	+	5.888600995	7.577489376	6.361935545	7.577019215	D3ZPC4;Q05695;Q05695-2
Adhesion and Cell Surface	Mllt4	similar to Afadin (Protein AF-6); myeloid	22.36453	23.82759	21.82581	21.84725	26.18747	25.49857	26.41233	26.09305	26.40027	25.3761	25.51367	26.72962	+	+	+	3.383877417	3.581560612	3.071449926	3.538621426	2;F1LT10;F1LWK4
Adhesion and Cell Surface	Ncam1	neural cell adhesion molecule 1	21.9863	23.13316	22.90291	20.80541	32.94805	32.08211	32.86703	32.52468	33.33285	32.50097	33.4268	33.06185	+	+	+	5.783364407	10.39851904	5.880206499	10.8736701	P13596;F1LNY3
Adhesion and Cell Surface	Ncam2	neural cell adhesion molecule 2	21.67848	22.3854	23.24089	22.43288	29.80335	29.32725	29.80589	29.68065	30.07839	29.45862	30.01824	29.67704	+	+	+	6.157489172	7.219872475	6.118293016	7.37365818	F1M8G9
Adhesion and Cell Surface	Ncan	neurocan	22.80171	21.9601	21.02922	24.59726	27.3533	25.71075	26.3423	25.70862	28.1716	26.5377	26.93078	27.21438	+	+	+	2.302864471	3.681667328	2.832002625	4.616539478	G3V8R2;P55067
Adhesion and Cell Surface	Ncdn	neurochondrin	21.61857	21.94582	22.44016	22.34815	28.31928	28.9954	29.6288	29.09534	28.97635	28.47003	29.5786	30.08752	+	+	+	6.12321905	6.921528816	5.718489506	7.189947128	O35095
Adhesion and Cell Surface	Nfasc	neurofascin homolog (chicken)	23.27377	21.78099	22.79475	21.92754	30.16507	30.56756	30.42992	30.45297	31.09622	31.31181	31.46699	31.05885	+	+	+	6.211705858	7.959618092	6.44904166	8.789208889	P97685;P97685-2;P97685-3
Adhesion and Cell Surface	Nlgn2	neuroligin 2	22.62537	22.31325	22.73815	22.26743	26.45533	26.61274	26.8125	26.39841	26.45252	26.81372	27.04843	26.77668	+	+	+	6.819510133	4.083692551	6.613841101	4.28678894	2;F1LQ41;D3ZJH3
Adhesion and Cell Surface	Nlgn3	neuroligin 3	21.77583	22.4632	23.55491	21.98501	27.60159	27.28947	27.42902	27.58592	27.96664	27.23745	27.63844	27.49366	+	+	+	4.787386521	5.0317626	4.710645352	5.139310837	2889-4;D4A2G5;Q62889-
Adhesion and Cell Surface	Nptn	neuroplastin	22.8977	23.09922	21.59269	22.13811	28.84715	27.53044	28.91946	28.56169	29.72297	28.89527	30.1488	30.19061	+	+	+	4.844316199	6.032753468	5.4047169	7.307482719	P97546;D3ZDF0;P97546-3
Adhesion and Cell Surface	Nrcam	neuronal cell adhesion molecule	21.71153	22.28858	22.38088	22.47057	29.18338	28.88145	28.60647	28.9597	29.56587	29.25881	29.0617	29.37499	+	+	+	7.216687007	6.694859982	7.463430869	7.102452278	7686-3
Adhesion and Cell Surface	Nrxn1	neurexin 1	21.56863	22.55182	23.17803	22.17206	28.08345	26.63062	26.5657	27.0167	28.30422	26.80082	28.01691	27.70326	+	+	+	4.153379798	4.706481457	4.559099211	5.338667393	12;Q63372-4;Q63372-
Adhesion and Cell Surface	Nrxn3	neurexin 3	23.00858	21.43452	23.3333	23.01214	27.37285	25.56063	26.77631	25.54262	27.67405	26.86325	26.78019	27.31976	+	+	+	2.931893363	3.615967274	4.078460569	4.462174416	2;Q07310-3;Q07310-
Adhesion and Cell Surface	Ntm	neurotrimin	23.35235	22.94086	22.41796	22.5926	28.80341	27.61534	28.6223	28.0626	29.02126	27.97667	29.55298	29.1205	+	+	+	5.422724936	5.449967861	5.341818838	6.091908932	G3V964;Q62718
Adhesion and Cell Surface	Odz4	odz, odd Oz/ten-m homolog 4 (Drosoph	21.75101	20.94875	23.33305	20.69489	27.01914	25.2123	25.70564	22.63336	25.86872	25.26119	27.08284	25.51743	+	+	+	1.709124656	3.46068716	2.983938522	4.250623703	F1LZ38
Adhesion and Cell Surface	Pcdh1	protocadherin 1	23.62003	22.28388	22.41312	22.84197	27.38895	26.79141	27.35397	26.28993	27.82972	26.99367	27.50074	27.26955	+	+	+	4.347093076	4.166315556	4.929331938	4.608667374	F1M8K1
Adhesion and Cell Surface	Pvrl1	poliovirus receptor-related 1 (herpesviru	22.66421	21.88717	23.63647	22.50321	26.53463	24.30522	26.66871	26.26817	26.99895	21.78169	27.12682	27.26446	+	+	+	2.589122939	3.271421909	1.184539995	3.120214462	F1LNP8
Adhesion and Cell Surface	Sdk2	sidekick homolog 2 (chicken)	21.95846	21.86951	22.19075	20.61	25.89252	24.57329	25.78342	23.05165	26.21212	25.09861	25.96426	25.64309	+	+	+	2.251771329	3.168039799	4.107361512	4.072339058	D3ZDA6
Adhesion and Cell Surface	Sez6l2	seizure related 6 homolog (mouse)-like	21.85245	21.94965	21.83514	21.70012	25.42307	22.06309	25.88092	26.11406	25.80997	27.2641	26.4476	26.34435	+	+	+	1.731721064	3.035945892	5.291394341	4.632167816	D4A6P1
Adhesion and Cell Surface	Tjp1	tight junction protein 1 (zona occludens	23.10633	22.07548	22.08296	23.1002	27.23114	25.74568	26.0231	25.63084	27.32783	25.65919	25.30156	26.20184	+	+	+	3.557632975	3.566450596	3.248601233	3.531362057	F1M4A0
Adhesion and Cell Surface	Tnc	tenascin C	21.84655	22.80317	21.62043	22.47538	25.02132	25.25021	25.84029	27.77656	26.11398	25.53112	27.59199	29.08631	+	+	+	2.836812061	3.785711765	2.942217443	4.894471169	M0RA80
Adhesion and Cell Surface	Tnr	tenascin R (restrictin, janusin)	22.15359	22.17787	24.5969	23.33479	28.99424	29.72874	30.20093	29.67966	29.25575	30.04986	30.1454	30.26349	+	+	+	4.343109836	6.585104465	4.476178869	6.862837315	F1LQ63
Adhesion and Cell Surface	Tnr	tenascin R (restrictin, janusin)	22.19742	22.95934	22.29406	23.03786	31.3604	31.42747	32.32574	31.33479	31.60452	31.80524	32.59508	32.37101	+	+	+	6.840401501	8.989931107	7.009827932	9.471795082	Q05546;Q05546-2
Adhesion and Cell Surface	Tprg1l	tumor protein p63 regulated 1-like	22.76952	22.63426	21.34704	21.64753	26.81641	27.29535	27.71947	27.20098	26.7898	27.27959	28.07674	27.325	+	+	+	4.875216018	5.158464909	4.671416648	5.268194199	A8WCF8
Adhesion and Cell Surface	Vcan	versican	23.42843	22.44795	21.19925	22.8137	29.78329	28.62083	29.63157	29.55298	30.02261	29.32294	30.10475	29.89865	+	+	+	4.865520169	6.924835205	5.199929386	7.36490345	2;D3ZFC3
Apoptosis	Birc6	baculoviral IAP repeat-containing 6	22.56974	22.39585	22.87736	22.14295	23.66101	27.89989	26.61639	26.19766	26.60894	26.68686	25.70203	27.3262	+	+	+	2.143048329	3.597264767	4.496435445	4.084533215	F1LY70
Apoptosis	Dad1	defender against cell death 1	22.33225	22.76075	22.25753	22.05824	26.69019	27.11694	25.96175	21.96265	27.01265	24.42683	25.74271	26.82043	+	+	+	1.38390428	3.08068943	2.999068631	3.648462772	P61805
Apoptosis	Pdcd6	aryl-hydrocarbon receptor repressor; pr	24.58966	21.20003	23.9407	22.587	27.00056	27.42662	27.22536	27.03065	27.33594	27.56848	27.00142	27.48784	+	+	+	2.774805726	4.09145546	2.857958025	4.269077778	G3V7W1
Apoptosis	Pdcd6ip	programmed cell death 6 interacting pro	21.10788	22.9684	21.79396	22.78345	27.23233	28.83096	27.04458	28.04171	27.27088	27.83906	28.02015	28.19864	+	+	+	4.084088212	5.62397337	4.650853296	5.668759823	Q9QZA2
APP	Apoo	apolipoprotein O	23.18925	21.79682	22.71557	22.67958	24.69636	26.0978	25.35881	25.83567	24.81817	25.89507	26.03368	25.89111	+	+	+	3.325414566	2.901853085	3.557285171	3.064199448	M0R7G4;M0R7V3
APP	App	amyloid beta (A4) precursor protein	22.46584	22.95816	21.72959	22.65298	27.97328	26.13935	27.36311	26.93787	27.0451	26.46284	26.51185	26.66384	+	+	+	4.235770617	4.651758194	5.156981498	4.219265938	P08592-2;P08592

Supplementary Table 2. List of proteins significantly enriched in both immunoisolated FEZ1 and Kinesin-1 containing vesicles by quantitative mass spectrometry

Group	Gene Symbol	Gene Name	log LFQ intensity IP1 Control	log LFQ intensity IP2 Control	log LFQ intensity IP3 Control	log LFQ intensity IP4 Control	log LFQ intensity IP1 Kinesin-1	log LFQ intensity IP2 Kinesin-1	log LFQ intensity IP3 Kinesin-1	log LFQ intensity IP4 Kinesin-1	log LFQ intensity IP1 FEZ1	log LFQ intensity IP2 FEZ1	log LFQ intensity IP3 FEZ1	log LFQ intensity IP4 FEZ1	t-test Significant Kinesin-1 IP	t-test Significant FEZ1 IP	t-test Significant Kinesin-1 and FEZ1 IP	-Log t-test p value Kinesin-1 IP	t-test Difference Kinesin-1 IP	-Log t-test p value FEZ1 IP	t-test Difference FEZ1 IP	Majority UniProt protein IDs
APP	Itm2c	integral membrane protein 2C	27.28533	21.85289	21.1172	22.43485	27.67909	27.53919	27.38673	26.84697	26.19672	27.58118	27.13104	26.44798	+	+	+	1.605086487	4.190426826	1.367174389	3.666662693	Q5PQL7
Autophagy	Atg9a	ATG9 autophagy related 9 homolog A (S	21.79725	22.81203	21.98062	22.95718	25.2126	24.84427	24.62193	25.58285	25.78307	25.25187	25.44455	25.02745	+	+	+	3.518971922	2.678641796	3.976230664	2.989966869	Q5FWU3
Axonal Growth	Crmp1	collapsin response mediator protein 1	23.08642	21.52036	25.17287	21.53155	29.73269	29.31913	29.9238	29.45166	29.80295	29.63215	29.94838	30.61114	+	+	+	3.615494052	6.779019356	3.708541425	7.170851231	Q62950
Axonal Growth	Dpysl2	dihydropyrimidinase-like 2	25.06546	22.35514	24.76491	27.90661	32.46893	32.18996	32.49042	32.49788	32.51202	32.85127	33.20474	33.16684	+	+	+	3.195243333	7.388765335	3.336282566	7.910681725	P47942
Axonal Growth	Dpysl3	dihydropyrimidinase-like 3	21.83787	20.64279	22.53059	21.20696	28.4056	26.63353	29.06134	29.11974	28.36811	27.92886	29.1125	29.0616	+	+	+	4.11745824	6.750499725	5.120127644	7.063214779	Q62952-2;Q62952
Axonal Growth	Gap43	growth associated protein 43	22.47329	22.24755	21.14741	23.96812	32.91709	28.61803	28.72351	29.40939	32.55465	28.83147	29.84504	29.06175	+	+	+	3.155577626	7.457913399	3.49426893	7.614134789	P07936
Axonal Growth	Lsamp	limbic system-associated membrane pro	23.52079	23.42987	22.43171	22.29379	26.61989	27.55377	27.61133	27.52283	26.75468	28.48727	28.75955	29.30965	+	+	+	4.477379542	4.407915115	3.827504161	5.408747673	Q62813-2;Q62813
Axonal Growth	Negr1	neuronal growth regulator 1	22.36157	22.47423	21.72253	22.10362	27.65854	27.33756	28.42001	29.06113	28.4883	22.85814	29.24118	28.46428	+	+	+	5.102834068	5.953822613	1.851651936	5.097487926	Q9Z0J8;D4A2V0
Axonal Growth	Tmem35	transmembrane protein 35	22.84548	22.89298	21.93064	23.73969	26.47186	27.3652	25.27291	26.69192	26.19946	27.14433	26.30226	26.33188	+	+	+	3.125710631	3.598275661	3.833856531	3.642286301	Q6JAM9
Channels and Receptors	Aqp4	aquaporin 4	20.41505	22.01504	23.7799	21.63262	29.12541	22.35135	27.82566	28.01079	28.4369	27.17083	27.32989	27.96229	+	+	+	1.570289085	4.867650032	3.580180459	5.764325142	P47863;P47863-2
Channels and Receptors	Atp1a1	ATPase, Na+/K+ transporting, alpha 1 po	30.05466	30.37513	30.06612	28.16816	33.52461	32.77898	33.58699	33.58318	33.391	33.22288	33.60234	33.62788	+	+	+	3.316352632	3.702421188	3.500656005	3.795007706	P06685
Channels and Receptors	Atp1a2	ATPase, Na+/K+ transporting, alpha 2 (+	32.27645	32.0796	32.26886	31.37316	35.75389	34.80468	35.48135	35.3559	35.61747	35.06041	35.52192	35.64323	+	+	+	4.575928442	3.34943819	5.020494099	3.461241245	P06686
Channels and Receptors	Atp1a3	ATPase, Na+/K+ transporting, alpha 3 po	31.634	31.08708	31.50876	30.33926	34.71108	33.72182	35.03809	34.71149	34.83069	34.28258	35.04066	35.04372	+	+	+	3.791057625	3.403344631	4.398057413	3.657137394	P06687
Channels and Receptors	Atp1b1	ATPase, Na+/K+ transporting, beta 1 pol	28.6702	28.07893	30.52757	30.06124	33.10738	31.65224	32.85423	33.14413	32.84373	32.58192	33.05897	32.57046	+	+	+	2.600319092	3.355010509	2.955511921	3.429284573	P07340
Channels and Receptors	Atp1b2	ATPase, Na+/K+ transporting, beta 2 pol	22.05947	21.09381	26.41347	21.71748	29.89865	29.8826	29.8499	30.25474	30.12213	30.72289	29.70581	29.86477	+	+	+	2.9666123	7.150415421	2.97737144	7.282846928	Q5M9H4;P13638
Channels and Receptors	Atp1b3	ATPase, Na+/K+ transporting, beta 3 pol	20.83748	23.3455	22.29216	22.89993	27.83623	28.333	29.39585	29.34872	28.73159	28.42166	29.65323	29.22672	+	+	+	4.122634042	6.384684086	4.456299499	6.664532185	Q63377
Channels and Receptors	Atp2a2	ATPase, Ca++ transporting, cardiac musc	28.216	28.28573	28.82841	27.4649	31.6486	31.70136	31.74367	31.85846	31.6186	31.55969	31.26752	31.64513	+	+	+	4.788952019	3.539261341	4.547332499	3.323972225	2;E9PSX6
Channels and Receptors	Atp2b1	ATPase, Ca++ transporting, plasma mem	26.31526	28.29018	27.77511	27.49465	33.13533	32.43356	32.89475	33.01199	33.23763	32.85573	33.1244	33.21943	+	+	+	4.717767221	5.400107384	4.931709196	5.640498161	4;P11505;F1LRW6;P11505-5
Channels and Receptors	Atp2b2	ATPase, Ca++ transporting, plasma mem	22.56387	21.49387	27.23462	27.05175	31.59602	31.42602	31.77529	31.95119	31.95622	31.79405	32.12037	32.21434	+	+	+	2.497319252	7.101103306	2.597764002	7.435217857	P11506;P11506-14;F1LP22
Channels and Receptors	Atp2b2	ATPase, Ca++ transporting, plasma mem	21.41157	22.90964	20.62935	21.44395	26.26635	25.31261	22.99626	26.02282	26.22852	25.82495	26.58678	26.59974	+	+	+	2.15455462	3.550884724	4.042308155	4.711372375	6;P11506-8;D4A8B3
Channels and Receptors	Atp2b3	ATPase, Ca++ transporting, plasma mem	22.13276	22.35934	23.6915	22.40362	28.05972	27.97574	28.58581	28.96185	28.14938	28.45631	28.9738	29.39615	+	+	+	5.008917025	5.748975277	5.008338479	6.09710598	9;Q64568-3;Q64568-
Channels and Receptors	Atp2b4	ATPase, Ca++ transporting, plasma mem	22.98243	21.96507	22.0803	21.61158	30.4897	30.04088	30.52281	30.30737	30.5517	30.45591	30.70644	30.42832	+	+	+	6.692582609	8.180345535	6.868329851	8.375748634	3;D3ZH00
Channels and Receptors	Cacna1e	calcium channel, voltage-dependent, R t	22.79506	22.91849	22.16845	19.85664	27.07398	23.4303	26.97629	26.18135	26.46443	24.83851	27.76135	26.82711	+	+	+	1.936600988	3.980822563	2.541724191	4.538191795	F1LMS1;F1LNB9;Q07652
Channels and Receptors	Cacna2d1	calcium channel, voltage-dependent, alp	23.9573	23.18929	22.09611	22.49133	29.62359	29.15079	30.18104	29.97473	29.74063	29.64437	30.22458	30.13173	+	+	+	5.1796655	6.799030781	5.445914033	7.001821995	D3ZKP9;F7F134
Channels and Receptors	Cacna2d2	calcium channel, voltage-dependent, alp	21.36127	22.81721	23.30588	22.5468	26.75595	24.14328	26.39342	26.20508	25.59605	22.42784	26.95116	26.68083	+	+	+	2.470494091	3.366647243	1.392121367	2.906181335	F1M9X7;Q8CFG6
Channels and Receptors	Cacna2d3	calcium channel, voltage-dependent, alp	22.31805	22.30011	24.49588	22.821	27.83008	27.50074	27.05569	26.56706	27.95082	27.84002	27.98245	27.61597	+	+	+	3.457880818	4.254632473	4.049093513	4.863554955	Q8CFG5;F1LSR8
Channels and Receptors	Cnrip1	cannabinoid receptor interacting protei	20.48345	22.03361	23.26536	23.5657	30.36066	30.52206	30.85013	30.71071	30.44559	30.66195	30.96313	30.73231	+	+	+	4.621254248	8.273864269	4.648631203	8.36371851	Q5M7A7
Channels and Receptors	Epha4	EPH receptor A4	21.76543	21.93007	21.60726	23.11677	27.63159	27.28012	27.57058	27.42374	28.17774	27.41877	27.87003	27.68412	+	+	+	5.297031312	5.371627331	5.256711809	5.682781696	D3ZZK3
Channels and Receptors	Gabbr1	gamma-aminobutyric acid (GABA) B rece	23.39384	23.1871	22.69466	22.02146	27.11793	25.65826	26.56417	26.66085	26.98685	26.28533	26.95338	26.67128	+	+	+	3.841890544	3.676040173	4.537256193	3.89994812	2;F7FE05;Q9Z0U4-4;Q9Z0U4-
Channels and Receptors	Gabbr2	gamma-aminobutyric acid (GABA) B rece	22.62023	22.60736	22.55323	21.96447	27.29325	26.18611	26.18132	26.20443	26.98696	26.42883	26.32248	26.66058	+	+	+	4.831802816	4.029955864	5.900663697	4.163391113	O88871
Channels and Receptors	Gnai1	guanine nucleotide binding protein (G p	22.79504	20.72953	22.65397	22.8025	29.72884	29.86691	29.65882	29.70142	30.36411	29.90999	29.78124	29.3764	+	+	+	5.212753665	7.493740082	5.072653726	7.61267519	P10824;F1LZ83
Channels and Receptors	Gnao1	guanine nucleotide binding protein (G p	21.67078	23.23473	23.02637	22.46769	28.24168	27.83225	28.98444	29.10107	28.05641	27.53416	28.97503	28.86281	+	+	+	4.862999454	5.939968109	4.643832003	5.757210732	D4ABT0;P59215-2
Channels and Receptors	Gnao1	guanine nucleotide binding protein (G p	29.16265	29.85803	30.81886	31.61092	34.08124	33.70852	34.0912	33.99463	34.02779	34.06968	33.79672	34.03477	+	+	+	3.243707339	3.606281281	3.268555786	3.619627953	P59215
Channels and Receptors	Gnb1	guanine nucleotide binding protein (G p	30.23695	29.3592	27.50415	29.39601	33.05816	32.91682	33.46466	33.5	33.65704	32.91116	33.74795	33.36137	+	+	+	3.342624592	4.110833168	3.399644525	4.295301437	P54311
Channels and Receptors	Gnb2	guanine nucleotide binding protein (G p	21.85596	22.97123	23.28692	28.19498	30.84041	30.32721	31.64736	31.42202	31.47697	30.722	32.17412	31.37906	+	+	+	2.547912576	6.98197794	2.662106128	7.36076498	P54313
Channels and Receptors	GNB2L1	guanine nucleotide binding protein (G p	23.05173	23.1806	21.08053	23.78229	26.17575	26.91616	26.90392	27.74894	26.8236	27.24082	27.39673	27.55896	+	+	+	3.09952983	4.162405968	3.498001411	4.481239319	P63245
Channels and Receptors	Gria1	glutamate receptor, ionotropic, AMPA 1	21.09134	22.49337	23.05441	21.57442	27.09023	26.50464	27.25593	27.45285	27.03695	27.00196	27.47746	27.42069	+	+	+	4.31122641	5.022526264	4.534193643	5.180879116	P19490;M0R5P7
Channels and Receptors	Gria2	glutamate receptor, ionotropic, AMPA 2	26.02848	21.96465	23.8557	22.749	29.33132	29.5775	29.09146	29.04416	29.44634	29.43116	29.62013	29.25013	+	+	+	3.126113381	5.611653805	3.211232092	5.787484646	P19491;P19491-3
Channels and Receptors	Gria3	glutamate receptor, ionotrophic, AMPA	21.67016	21.3259	22.60779	22.88781	27.38583	26.87783	27.25503	27.20955	26.82881	26.93382	27.50847	27.36453	+	+	+	4.905919474	5.059144974	4.771577623	5.035992146	P19492
Channels and Receptors	Grid2	glutamate receptor, ionotropic, delta 2	22.01838	21.04793	24.02759	22.11781	25.08404	23.78212	25.86514	26.70951	22.10296	25.83009	25.72858	27.74439	+	+	+	1.876839294	3.057274818	1.206465354	3.048577785	Q63226;F1LXB6
Channels and Receptors	Grin1	glutamate receptor, ionotropic, N-meth	22.27083	21.22078	23.00297	22.35372	27.05445	22.13317	27.68992	27.02147	27.11098	27.24191	27.92411	27.25764	+	+	+	1.50921407	3.762674332	4.807423734	5.171581268	4;P35439-6;P35439-
Channels and Receptors	Grin2b	glutamate receptor, ionotropic, N-meth	24.25856	24.20862	22.2976	23.0681	28.43762	25.8328	27.14102	26.46287	27.97564	27.58735	28.55801	27.20564	+	+	+	2.519717248	3.510355473	3.653046301	4.373437405	G3V746;Q00960
Channels and Receptors	Grm2	glutamate receptor, metabotropic 2	22.74904	22.838	21.68485	21.56325	26.5137	26.26105	27.15779	27.1487	26.45362	26.40711	26.64238	26.4634	+	+	+	4.514907972	4.561527252	4.792993062	4.282844067	P31421
Channels and Receptors	Grm3	glutamate receptor, metabotropic 3	22.89075	21.5298	22.83654	22.51254	28.6059	27.26517	28.18253	28.20051	28.53293	27.29658	28.39648	28.34921	+	+	+	4.942979086	5.621121407	4.972566727	5.701394558	P31422
Channels and Receptors	Kcna2	potassium voltage-gated channel, shake	22.20705	22.50521	21.95276	21.94392	25.33644	25.78669	26.36721	25.68357	25.95338	26.21786	26.94916	26.95815	+	+	+	5.163906853	3.64124155	5.280012371	4.367401123	P63142;P15384
Channels and Receptors	Kcnab2	potassium voltage-gated channel, shake	22.28233	22.8712	22.81891	21.82152	28.20238	28.47854	28.56881	28.83376	27.87549	28.25755	28.24304	28.5856	+	+	+	6.203413108	6.072380543	6.015990278	5.791927814	P62483
Channels and Receptors	Kcnj10	potassium inwardly-rectifying channel, s	22.0159	22.01138	22.66571	21.57105	26.68325	26.27441	26.07261	21.73182	26.25649	26.58305	26.0299	25.32862	+	+	+	1.416598346	3.124513149	4.570052107	3.983503819	P49655
Channels and Receptors	Kcnma1	potassium large conductance calcium-ac	22.55459	23.51874	23.55199	23.31191	25.86592	25.40996	25.94505	25.73322	26.16068	24.8974	26.09194	26.40712	+	+	+	4.136019813	2.504229546	3.191428649	2.654977798	8;Q62976-3;Q62976-
Channels and Receptors	Lgalsl	lectin, galactoside-binding-like	22.5639	22.62089	22.95794	22.91105	26.232	25.63533	25.76159	25.76719	26.18175	25.83072	25.92177	26.35252	+	+	+	5.822256063	3.085585594	6.147974546	3.308247566	B4F7A3
Channels and Receptors	Lphn1	latrophilin 1	21.73546	23.503	23.22173	22.51199	25.96908	26.83702	26.45007	26.00977	26.59278	26.52093	26.4651	26.16636	+	+	+	3.699301653	3.573441982	4.002246878	3.693247318	4;D3ZBB0;D4A144;O88917-
Channels and Receptors	Ntrk2	neurotrophic tyrosine kinase, receptor,	22.18351	22.89701	22.95324	21.93531	26.42385	26.8853	26.8597	26.92445	27.36135	27.15817	27.75181	27.3692	+	+	+	5.297838862	4.281057358	5.626346397	4.917863846	Q63604
Channels and Receptors	Pgrmc1	progesterone receptor membrane comp	21.29558	22.7495	22.37621	22.82294	28.47862	28.82089	28.32096	28.29119	29.17369	28.63962	28.7831	29.36661	+	+	+	5.507675575	6.166856289	5.591986527	6.67969799	P70580
Channels and Receptors	Pgrmc2	progesterone receptor membrane comp	23.41946	23.1668	22.46684	21.99635	25.49409	25.40133	25.34045	25.20101	26.15522	25.60102	25.51343	25.81399	+	+	+	3.644522841	2.596856594	3.829539208	3.008554459	Q5XIU9
Channels and Receptors	Ryr2	ryanodine receptor 2 (cardiac)	22.35169	23.12002	21.56075	22.72005	29.89894	30.33575	30.13369	30.07137	29.91371	30.14759	30.09786	30.10387	+	+	+	6.275094845	7.671812534	6.320904162	7.62762785	2;F1LS89;F1LRZ1
Channels and Receptors	S1pr1	sphingosine-1-phosphate receptor 1	23.15796	21.48313	21.09188	22.53981	26.25003	25.68811	25.50543	25.66145	25.36289	25.97636	25.94949	25.74845	+	+	+	3.500367021	3.70805788	3.520647903	3.691099644	P48303
Channels and Receptors	Scn2a	sodium channel, voltage-gated, type II, a	22.28991	22.07825	21.64723	22.24457	29.80281	29.29667	30.04009	29.68169	29.51047	29.33127	30.50351	30.19308	+	+	+	7.499063861	7.640323639	6.56058174	7.819591045	P04775
Channels and Receptors	Scn2b	sodium channel, voltage-gated, type II, b	22.63264	22.30303	21.98997	21.88663	28.16744	26.3298	27.48278	25.8768	28.70754	27.37443	28.28947	27.00624	+	+	+	3.878770355	4.761139393	4.924272798	5.641356945	P54900
Channels and Receptors	Sirpa	signal-regulatory protein alpha	22.20708	21.75659	21.51277	22.3676	31.31587	30.49305	30.87225	30.79025	31.18098	31.0177	31.11948	31.23917	+	+	+	7.377651785	8.906843185	8.103117067	9.178322315	P97710
Channels and Receptors	Thy1	Thy-1 cell surface antigen	29.1497	30.03328	29.15197	29.63552	33.07166	31.71301	32.96046	32.87571	32.47141	31.68571	32.73574	32.93865	+	+	+	3.775547811	3.162593365	3.845955793	2.965256214	P01830
Chaperone	Cct2	chaperonin containing TCP1, subunit 2 (	23.33125	20.56204	25.80546	22.07597	26.42996	27.2615	26.59463	26.04514	25.39175	26.79464	26.9901	27.80119	+	+	+	1.729422029	3.639129639	1.688066456	3.800743103	Q5XIM9
Chaperone	Cct3	chaperonin containing TCP1, subunit 3 (	22.26936	23.31243	22.75498	21.99129	25.23246	23.06987	26.63727	26.87279	26.18412	22.58364	26.73092	27.24146	+	+	+	1.686899694	2.87108469	1.525330631	3.103016853	Q6P502

Supplementary Table 2. List of proteins significantly enriched in both immunoisolated FEZ1 and Kinesin-1 containing vesicles by quantitative mass spectrometry

Group	Gene Symbol	Gene Name	log LFQ intensity IP1 Control	log LFQ intensity IP2 Control	log LFQ intensity IP3 Control	log LFQ intensity IP4 Control	log LFQ intensity IP1 Kinesin-1	log LFQ intensity IP2 Kinesin-1	log LFQ intensity IP3 Kinesin-1	log LFQ intensity IP4 Kinesin-1	log LFQ intensity IP1 FEZ1	log LFQ intensity IP2 FEZ1	log LFQ intensity IP3 FEZ1	log LFQ intensity IP4 FEZ1	t-test Significant Kinesin-1 IP	t-test Significant FEZ1 IP	t-test Significant Kinesin-1 and FEZ1 IP	-Log t-test p value Kinesin-1 IP	t-test Difference Kinesin-1 IP	-Log t-test p value FEZ1 IP	t-test Difference FEZ1 IP	Majority UniProt protein IDs
Chaperone	Cct4	chaperonin containing TCP1, subunit 4 (	22.33447	22.03936	22.9319	23.14298	26.44821	26.23734	26.47135	25.64691	25.54295	23.2761	26.22839	27.81134	+	+	+	4.519634764	3.588778496	1.719296076	3.102519989	Q7TPB1
Chaperone	Cct7	chaperonin containing TCP1, subunit 7 (	21.84281	21.51248	21.28223	21.738	26.63048	23.09094	25.69328	22.49189	27.29763	22.73812	26.14711	25.59798	+	+	+	1.542366237	2.882769108	2.118094125	3.851331234	D4AC23
Chaperone	Clu	clusterin	23.38351	22.92301	21.89059	22.1987	26.79377	26.0936	26.24924	26.24274	26.52367	25.77334	26.07714	26.0938	+	+	+	4.249777262	3.74588728	4.093761588	3.518037796	G3V836;P05371
Chaperone	Dnajc13	DnaJ (Hsp40) homolog, subfamily C, me	22.47972	22.23379	21.78287	21.54682	26.90093	26.99108	27.50786	27.38624	26.70147	26.93596	27.49876	26.78556	+	+	+	6.005715707	5.185725212	5.711317566	4.969638824	D3ZNI6;D3ZN27
Chaperone	Dnajc5	DnaJ (Hsp40) homolog, subfamily C, me	23.11299	22.97796	23.07966	21.89899	30.10237	28.51537	28.74756	29.19587	30.44342	28.74496	29.43374	29.56848	+	+	+	5.080679775	6.372890949	5.24501495	6.780252457	P60905
Chaperone	Hsp90aa1	heat shock protein 90kDa alpha (cytosol	28.48953	28.56009	29.79349	29.48725	32.58208	32.10603	32.20087	32.54038	32.71067	32.00965	32.62712	32.61292	+	+	+	4.07674903	3.274748325	4.060996174	3.40749979	P82995
Chaperone	Hsp90b1	heat shock protein 90kDa beta (Grp94),	23.0671	22.93641	25.92259	23.11498	30.86791	30.22906	30.71071	30.79242	30.60329	30.73409	30.50616	30.65121	+	+	+	4.074298601	6.889756203	4.107534322	6.863417149	Q66HD0;Q66HD0-2
Chaperone	Hspa4	heat shock 70kDa protein 4	23.25984	21.60474	22.3699	22.59786	29.56975	29.46494	30.38803	30.13284	29.7	29.59612	30.26763	30.39713	+	+	+	5.759195666	7.430800915	5.865744815	7.532135487	F1LRV4;O88600
Chaperone	Hspa4l	heat shock 70kDa protein 4-like	22.93699	22.5838	22.22919	22.17892	28.33411	28.35439	28.67751	28.68415	28.37985	28.57232	29.01972	29.01502	+	+	+	7.036211606	6.030315876	6.693021672	6.264502048	B4F772
Chaperone	Hspa5	hypothetical gene supported by AF2162	25.52746	21.55853	27.01701	22.57426	31.21013	31.44953	31.19642	31.10218	31.11694	31.76959	31.46334	31.04876	+	+	+	2.845228848	7.070250511	2.864411252	7.180339813	P06761
Chaperone	Hspa8	heat shock 70kDa protein 8	30.3226	30.85289	30.66382	30.50919	33.36372	33.48183	33.87041	33.61632	33.37968	33.55476	33.81077	33.89546	+	+	+	5.878321879	2.995948792	5.835819847	3.073045731	R8M9;D4A4S3
Chaperone	Hsph1	heat shock 105kDa/110kDa protein 1	21.388	23.03449	21.6389	22.24544	29.40129	28.9548	30.06561	29.69102	29.52811	29.21625	29.78202	30.00129	+	+	+	5.595545171	7.451472282	5.824081169	7.55521059	Q66HA8
Chaperone	Hyou1	hypoxia up-regulated 1	23.13359	22.60097	22.35209	24.00764	28.60573	27.36953	29.04973	29.07388	28.745	28.41199	29.158	28.78366	+	+	+	4.272923772	5.501143932	5.169867158	5.751092434	F1LN18;Q63617
Chaperone	Lrpap1	low density lipoprotein receptor-relatec	21.75472	22.13766	23.04399	20.94357	26.74227	25.86938	26.09102	26.50691	26.55445	23.14355	25.0203	26.46863	+	+	+	3.991744979	4.332411289	1.972199531	3.326752663	Q99068
Chaperone	Sgta	small glutamine-rich tetratricopeptide r	23.15879	22.34694	22.23796	22.62326	25.7044	25.48511	25.75804	26.54799	26.30492	25.89127	26.5018	26.64361	+	+	+	4.376658779	3.282149792	5.126362758	3.743665695	O70593
Cytoskeletal Motor Related	Dctn1	dynactin 1 (p150, glued homolog, Droso	22.33328	22.9013	22.3046	22.73908	28.62317	29.67786	29.13284	28.88457	28.30003	29.20639	29.43633	29.71749	+	+	+	6.479954606	6.510046005	5.909452039	6.595496178	D4A8U7;P28023
Cytoskeletal Motor Related	Dctn2	dynactin 2 (p50)	22.56987	22.5026	26.51608	21.18364	26.19316	27.0879	27.36995	27.98587	27.29999	26.83557	27.89504	27.52507	+	+	+	1.770791803	3.966172218	1.931083772	4.195870876	Q6AYH5
Cytoskeletal Motor Related	Dctn3	dynactin 3 (p22)	21.4213	21.81363	23.00353	22.55107	27.27533	27.1831	27.09406	26.93618	27.17722	26.13721	26.89689	26.68137	+	+	+	4.99746914	4.924787521	4.429233716	4.525792599	D4A1B8
Cytoskeletal Motor Related	Dynll1	dynein, light chain, LC8-type 1	20.9634	22.03052	22.09262	22.4107	27.61477	29.79388	28.16394	22.39092	28.7024	30.10787	27.331	22.46388	+	+	+	1.694544572	5.116565704	1.686113029	5.276972771	P63170
Cytoskeletal Motor Related	Dynll2	dynein, light chain, LC8-type 2	29.73999	29.03617	22.48513	22.16658	30.98421	30.45826	30.44016	29.75247	30.32914	31.62593	30.10362	30.60276	+	+	+	1.159818743	4.55180645	1.226105144	4.808393955	Q78P75
Cytoskeletal Motor Related	Dynlrb1	dynein, light chain, roadblock-type 1	23.43427	24.1395	21.68314	23.33802	27.70431	30.09673	28.41131	28.86874	28.19037	29.13205	28.02094	29.1305	+	+	+	3.621376424	5.621539116	4.011057889	5.469734192	P62628
Cytoskeletal Motor Related	Kif21a	kinesin family member 21A	22.72574	22.47773	22.46289	21.66332	28.05915	27.87941	28.23558	28.02702	28.01452	28.09315	28.55468	28.72826	+	+	+	6.419885779	5.717868328	6.094263493	6.015232086	ZJJ7;D4A1V5
Cytoskeletal Motor Related	Kif2a	kinesin heavy chain member 2A	23.42799	22.28051	24.84364	20.61366	27.662	26.87923	27.17798	27.21067	26.2465	26.41408	26.89285	27.34866	+	+	+	2.558645364	4.441020489	2.261232338	3.934070587	2;Q9WV63;F1M745;F1M8L1
Cytoskeletal Motor Related	Kif5b	kinesin family member 5B	22.90785	23.7137	21.75844	23.3926	28.92912	29.79974	28.83237	28.66601	27.05662	26.14026	26.52281	25.76853	+	+	+	4.751416081	6.11366415	3.282771205	3.428909302	Q2PQA9
Cytoskeletal Motor Related	Klc2	kinesin light chain 2	23.67409	23.22341	23.27105	21.91358	29.0694	26.15677	28.41687	28.37696	25.50948	26.18809	26.21686	24.77676	+	+	+	3.275367988	4.984470844	2.686806112	2.652267456	B2GV74
Cytoskeletal Motor Related	Ktn1	kinectin 1 (kinesin receptor)	22.99575	22.91395	22.98619	21.56329	26.42717	25.32003	25.28926	26.33209	27.97498	25.50942	24.98889	25.43667	+	+	+	3.333270404	3.227344036	2.3478985	3.362695217	D4A4Z9;D4AD67;D4ADW6
Cytoskeletal Motor Related	LOC10036	axonal transport of synaptic vesicles-like	22.62519	24.19785	22.29766	23.16734	27.63333	26.96818	27.4932	27.82007	27.85555	27.349	27.72592	27.98696	+	+	+	4.16159634	4.406686306	4.389283276	4.65734911	1LNY5
Cytoskeletal Motor Related	Mapk8ip3	mitogen-activated protein kinase 8 inter	23.8126	22.27704	21.75221	22.2349	27.68224	26.50325	26.67357	26.64883	26.70662	26.15821	25.61181	26.32521	+	+	+	3.789935085	4.357786179	3.48452996	3.681274414	E9PSK7
Cytoskeletal Motor Related	Myh10	myosin, heavy chain 10, non-muscle	21.49973	26.31223	26.34531	22.37936	33.75941	32.72915	32.71067	32.56202	33.356	32.69907	32.71976	32.57977	+	+	+	3.279793571	8.806151867	3.28393241	8.704489231	G3V9Y1;Q9JLT0
Cytoskeletal Motor Related	Myh14	myosin, heavy chain 14	21.60301	23.87977	23.17033	22.59037	28.38714	27.00002	27.1025	27.50066	27.70853	27.33262	27.78101	27.79538	+	+	+	3.734518764	4.686713219	4.191232053	4.843515873	F1LNF0
Cytoskeletal Motor Related	Myh9	myosin, heavy chain 9, non-muscle	21.07062	21.59199	21.8967	22.41605	30.62767	30.32066	30.20688	30.12608	30.36442	30.22561	30.17367	30.13345	+	+	+	6.896442119	8.57648325	7.010212236	8.480451107	G3V6P7;Q62812
Cytoskeletal Motor Related	Myl12b	myosin, light chain 12B, regulatory	20.54227	22.28154	26.98609	26.88844	29.56778	28.16188	27.57586	27.81623	29.80386	28.49587	27.83274	28.69724	+	+	+	1.287477647	4.105848789	1.443395117	4.532839775	P18666;P13832
Cytoskeletal Motor Related	Myl6	myosin, light chain 6, alkali, smooth mu:	28.54056	27.84362	22.86946	22.10835	29.80275	28.9196	28.16907	28.37161	29.68926	29.26667	28.61712	28.83741	+	+	+	1.061202828	3.475257397	1.180423305	3.762115955	Q64119
Cytoskeletal Motor Related	Myo1d	myosin ID	22.35307	22.16961	21.62551	23.60414	25.75677	23.58085	25.28588	24.65655	24.60846	24.61268	25.08363	25.20113	+	+	+	2.038934173	2.381927967	2.805402705	2.438389301	Q63357
Cytoskeletal Motor Related	Myo5a	myosin VA (heavy chain 12, myoxin)	21.70988	27.98044	27.94302	26.72858	33.03556	32.09481	32.46329	32.24295	32.39164	32.27189	31.94918	32.2533	+	+	+	2.262967614	6.368672848	2.199815354	6.126022816	Q9QYF3
Cytoskeletal Motor Related	Myo6	myosin VI	23.25452	21.44876	22.99315	22.46351	27.50832	27.83292	27.89862	27.89851	28.69691	27.92751	28.21155	28.29312	+	+	+	4.853353538	5.244607925	4.964051702	5.742286205	D4A5I9;D3ZYX5
Cytoskeletal Motor Related	Nudc	nuclear distribution gene C homolog (A.	22.2721	22.87846	20.80576	23.86676	27.37119	26.15201	27.27737	26.17076	27.14092	25.88888	27.12129	27.08811	+	+	+	2.986994634	4.287064075	3.063887764	4.354031563	Q63525;M0R9U5
Cytoskeleton Related	Actn1	actinin, alpha 1	21.21141	26.72845	26.29153	22.73954	30.32195	29.90009	29.7514	29.99064	30.49295	30.8027	29.98251	29.88837	+	+	+	2.266998091	5.748286724	2.353734351	6.04889822	Q9Z1P2
Cytoskeleton Related	Actn2	actinin, alpha 2	23.34789	21.81644	22.59318	21.66813	28.81928	28.09093	27.97711	28.06409	29.35651	28.42634	28.04932	27.57802	+	+	+	4.998236212	5.881443024	4.49404347	5.996137142	D3ZCV0
Cytoskeleton Related	Actn4	actinin, alpha 4	22.87284	22.9901	22.4945	22.0907	28.28229	27.77536	28.08431	28.00999	28.17764	27.98886	28.2562	27.85013	+	+	+	6.433026633	5.425951481	6.511452819	5.456172943	Q9QXQ0
Cytoskeleton Related	ACTR1A	ARP1 actin-related protein 1 homolog A	23.06005	22.66327	22.10648	24.89091	29.12166	29.18957	29.42598	30.00731	28.77897	29.11798	28.97736	29.58569	+	+	+	4.198103419	6.255952358	4.102295601	5.934817791	P85515
Cytoskeleton Related	Actr1b	ARP1 actin-related protein 1 homolog B	21.94946	22.43453	21.93236	22.52065	26.85626	22.10814	27.50552	27.6725	26.84302	20.81184	27.55296	27.18499	+	+	+	1.550082206	3.826354504	1.097605596	3.388953209	B2RYJ7
Cytoskeleton Related	Actr2	ARP2 actin-related protein 2 homolog (y	22.64907	22.27905	25.89807	21.18301	28.90461	28.36799	28.81607	29.55234	28.85371	28.56982	28.93143	29.01659	+	+	+	2.885112151	5.907954216	2.912437126	5.840589523	Q5M7U6
Cytoskeleton Related	Actr3	ARP3 actin-related protein 3 homolog (y	22.94994	21.81841	22.34353	23.0004	28.84529	23.75112	28.40548	28.81366	28.99399	28.02089	29.39946	28.67192	+	+	+	2.087795282	4.925819874	5.335015751	6.243496418	Q4V7C7
Cytoskeleton Related	Arpc1a	actin related protein 2/3 complex, subu	22.84802	22.03192	21.69668	22.85723	28.79033	28.23695	28.66418	28.89966	28.93677	28.51552	28.91274	28.45744	+	+	+	5.890684492	6.289313316	5.974326898	6.347152233	Q99PD4;D3ZXP7;D4A992
Cytoskeleton Related	Arpc2	actin related protein 2/3 complex, subu	21.97865	22.64219	21.69194	22.56965	29.33106	27.7557	29.06116	28.88809	28.95568	28.4857	29.1487	28.97482	+	+	+	5.374641036	6.538396358	6.531767921	6.670618057	P85970
Cytoskeleton Related	Arpc3	similar to actin related protein 2/3 com|	22.18862	22.29542	23.07154	22.99881	29.09635	27.35111	28.11674	28.06317	28.15117	27.5223	27.73253	28.51593	+	+	+	4.883954629	5.51824522	5.534540695	5.341888905	B2GV73
Cytoskeleton Related	Arpc4	tubulin tyrosine ligase-like family, meml	22.28159	21.07883	27.6284	27.3575	29.83686	30.0747	30.12743	29.88457	29.71784	30.14674	29.9348	29.95795	+	+	+	1.717545889	5.394312859	1.703276573	5.35275507	B2RZ72
Cytoskeleton Related	Arpc5l	actin related protein 2/3 complex, subu	21.22223	22.58023	21.20917	22.24686	27.83503	27.57687	28.06199	27.39795	27.92982	27.66729	28.21609	28.22177	+	+	+	5.337744263	5.903334618	5.492501879	6.194120884	A1L108;G3V7U8;A1L108-2
Cytoskeleton Related	Basp1	brain abundant, membrane attached sig	22.67991	22.68021	23.36011	22.07189	30.64324	30.69884	30.04934	29.96044	30.93726	30.32721	31.63197	30.8181	+	+	+	6.397272197	7.639935017	6.223902794	8.230605125	Q05175
Cytoskeleton Related	Cap1	CAP, adenylate cyclase-associated prote	24.51261	21.69466	22.1169	22.48735	27.83497	27.25494	28.1153	28.06635	28.74881	26.11466	29.38033	28.64179	+	+	+	3.633311771	5.115013123	2.933127077	5.518517494	Q08163
Cytoskeleton Related	Cfl1	cofilin 1 (non-muscle)	29.54631	29.50675	31.97248	31.95965	32.90363	33.1479	33.38895	33.54155	33.24898	33.18201	33.28016	33.25912	+	+	+	1.881669126	2.499213696	1.914445107	2.496272564	P45592
Cytoskeleton Related	Cfl2	cofilin 2 (muscle)	23.00368	21.2203	21.98331	21.30017	27.64182	28.07168	27.71371	28.23859	28.15596	28.08745	27.40682	28.09089	+	+	+	5.050532696	6.039586544	4.987919045	6.058411598	M0RC65
Cytoskeleton Related	Ckap5	cytoskeleton associated protein 5	20.72125	21.43216	27.4516	26.17429	28.29986	28.57503	28.73834	28.64441	29.1656	28.27755	28.52047	28.84972	+	+	+	1.472621061	4.619584084	1.511818803	4.758508682	F1M949;F1M0X7
Cytoskeleton Related	Clasp2	cytoplasmic linker associated protein 2	22.6966	22.85952	22.074	21.74464	27.44947	27.28313	26.92558	26.00097	29.78837	27.37219	27.26598	27.69737	+	+	+	4.470205634	4.571097374	3.915704577	5.687283993	Q99JD4
Cytoskeleton Related	Clip2	CAP-GLY domain containing linker prote	21.98026	23.00544	26.50543	22.48174	26.58663	27.02094	27.03988	25.7102	28.31389	27.63907	26.80661	27.48185	+	+	+	1.556896054	3.096195698	2.045541065	4.067137718	G3V949;O55156
Cytoskeleton Related	Cnpy2	canopy 2 homolog (zebrafish)	22.23998	22.52847	22.56274	22.71148	25.87593	26.89111	26.447	26.58377	26.5159	26.78432	25.99397	26.70491	+	+	+	5.548399019	3.938785553	5.94529366	3.989106178	A0JN30
Cytoskeleton Related	Coro1a	coronin, actin binding protein, 1A	21.98703	21.38584	22.3807	22.39605	28.45795	26.90058	28.05564	28.12352	27.66824	27.2179	28.53063	28.63111	+	+	+	5.103995347	5.847014904	5.14794239	5.974560738	Q91ZN1
Cytoskeleton Related	Coro1c	coronin, actin binding protein, 1C	21.5199	23.26079	21.49169	22.54488	25.27547	25.63851	25.48536	26.08103	25.69128	22.67253	26.00851	26.36046	+	+	+	3.504165628	3.415780544	1.694537538	2.978882313	G3V624
Cytoskeleton Related	Cotl1	coactosin-like 1 (Dictyostelium)	22.61922	21.85888	23.23873	23.38431	27.98207	28.25867	28.83596	28.77254	27.63672	28.1335	29.04325	28.84221	+	+	+	5.096253386	5.687023163	4.663044422	5.638631821	B0BNA5
Cytoskeleton Related	Csrp1	cysteine and glycine-rich protein 1	22.25226	22.17838	22.02936	22.44075	27.24445	26.14354	26.56612	26.76838	27.50839	22.90178	26.64691	26.59591	+	+	+	5.759160821	4.455434799	1.93613472	3.688063622	P47875

Supplementary Table 2. List of proteins significantly enriched in both immunoisolated FEZ1 and Kinesin-1 containing vesicles by quantitative mass spectrometry

Group	Gene Symbol	Gene Name	log LFQ intensity IP1 Control	log LFQ intensity IP2 Control	log LFQ intensity IP3 Control	log LFQ intensity IP4 Control	log LFQ intensity IP1 Kinesin-1	log LFQ intensity IP2 Kinesin-1	log LFQ intensity IP3 Kinesin-1	log LFQ intensity IP4 Kinesin-1	log LFQ intensity IP1 FEZ1	log LFQ intensity IP2 FEZ1	log LFQ intensity IP3 FEZ1	log LFQ intensity IP4 FEZ1	t-test Significant Kinesin-1 IP	t-test Significant FEZ1 IP	t-test Significant Kinesin-1 and FEZ1 IP	-Log t-test p value Kinesin-1 IP	t-test Difference Kinesin-1 IP	-Log t-test p value FEZ1 IP	t-test Difference FEZ1 IP	Majority UniProt protein IDs
Cytoskeleton Related	Cttn	cortactin	23.68242	22.17693	21.84665	23.14101	27.39885	25.63622	26.89076	23.09246	28.14414	27.27062	27.36236	26.79526	+	+	+	1.56048208	3.0428195	4.035019212	4.681341171	Q66HL2;D3ZGE6
Cytoskeleton Related	Cyfip1	cytoplasmic FMR1 interacting protein 1	22.19838	22.58588	26.48505	21.9734	30.07253	29.54455	30.10587	30.00691	30.37793	29.79014	30.41246	30.42982	+	+	+	3.078609623	6.621789455	3.18074767	6.941911697	D4A8H8
Cytoskeleton Related	Dbn1	drebrin 1	23.19369	22.45873	27.82966	22.83522	30.47547	29.8763	29.8229	29.42876	30.69752	29.94098	29.82384	30.15388	+	+	+	2.413093351	5.821533203	2.508830886	6.074730396	Q07266;Q07266-2
Cytoskeleton Related	Dst_predic	dystonin	21.9254	22.94118	21.75488	23.11243	26.95382	28.34659	27.50937	27.78226	27.83966	28.43762	28.267	27.96791	+	+	+	4.597102281	5.214539528	5.308479302	5.69457531	LU52
Cytoskeleton Related	Dstn	destrin (actin depolymerizing factor)	22.86032	22.04911	22.3186	27.19338	29.18497	28.98291	29.35473	29.49413	28.95208	29.31357	29.17424	29.46037	+	+	+	2.458824829	5.648832798	2.448279841	5.619711876	Q7M0E3;D4A315
Cytoskeleton Related	Ezr	hypothetical protein LOC100129652; ez	23.28249	23.20695	22.33063	21.85341	30.07648	29.03244	28.53879	28.7474	29.61558	29.22773	28.82581	28.76708	+	+	+	4.934977132	6.430406094	5.442502203	6.440679073	P31977
Cytoskeleton Related	Fry	furry homolog (Drosophila)	22.63849	22.72056	22.84886	21.94917	26.96014	28.7514	27.53786	21.56993	28.59811	30.14868	29.87545	27.58585	+	+	+	1.20677336	3.665558815	4.329936618	6.512747288	E9PTY6
Cytoskeleton Related	Fscn1	fascin homolog 1, actin-bundling protei	22.74627	22.46247	22.25425	22.39913	29.95934	22.39825	29.22509	29.72415	28.83126	23.178	28.6187	29.39893	+	+	+	1.590140809	5.361176491	1.872049046	5.041191578	P85845
Cytoskeleton Related	Gsn	gelsolin (amyloidosis, Finnish type)	22.37373	22.87218	26.03593	21.91756	29.70545	28.93267	28.00635	27.52126	29.42005	28.82696	28.50635	28.56416	+	+	+	2.603485432	5.241580486	2.933169189	5.529531002	Q68FP1;Q68FP1-2
Cytoskeleton Related	Lasp1	LIM and SH3 protein 1	22.55931	23.22382	21.61728	21.45557	29.70662	25.62148	27.28798	26.88483	28.6071	28.17603	26.9266	26.76497	+	+	+	2.792397088	5.161236286	3.912207873	5.404681683	Q99MZ8
Cytoskeleton Related	Macf1	microtubule-actin crosslinking factor 1	23.01331	21.80131	23.88722	22.55384	29.8171	30.54342	30.23638	30.21292	30.67548	30.70726	30.76841	30.42782	+	+	+	5.425910055	7.388532162	5.678459748	7.830821514	D3ZHV2
Cytoskeleton Related	Map1s	microtubule-associated protein 1S	22.18386	21.83402	22.29171	22.48215	23.89477	25.13324	24.75598	24.40224	24.44323	24.76956	24.71623	24.6418	+	+	+	3.666551237	2.348622322	5.406765845	2.444770336	P0C5W1
Cytoskeleton Related	Map6	microtubule-associated protein 6	22.9998	27.35422	29.22853	27.68679	30.5845	30.08942	30.67337	30.17331	30.68403	30.40294	30.42142	30.55161	+	+	+	1.420036901	3.56281662	1.485466988	3.69766283	Q63560;F1LQZ9
Cytoskeleton Related	Mapre1	microtubule-associated protein, RP/EB f	22.77458	22.87203	24.06687	21.39621	27.72871	27.51998	27.81549	26.88821	27.88017	26.40477	27.38212	27.5974	+	+	+	3.70765941	4.71067524	3.429063242	4.538691998	Q66HR2
Cytoskeleton Related	Mapre2	microtubule-associated protein, RP/EB f	23.53014	21.46693	21.9287	26.99809	27.78144	28.27866	28.21109	27.97246	28.63599	27.85287	28.27075	28.41454	+	+	+	1.965339972	4.579947472	2.05144745	4.812573433	Q3B8Q0;M0R7M8;M0R5N3
Cytoskeleton Related	Mapre3	microtubule-associated protein, RP/EB f	22.29255	22.41432	27.54562	25.86762	28.58721	28.69049	28.49697	28.48405	28.71777	28.96375	28.73905	28.69853	+	+	+	1.672594372	4.034655571	1.762424371	4.249752998	Q5XIT1
Cytoskeleton Related	Mapt	microtubule-associated protein tau	21.65771	21.99808	27.43841	22.39794	30.31883	28.72988	28.8444	28.07903	29.41498	28.73295	29.76902	29.63879	+	+	+	2.094774099	5.619999409	2.316824249	6.015902996	E9PTR5;P19332-3;P19332-7
Cytoskeleton Related	Marcks	myristoylated alanine-rich protein kinas	22.50938	22.19488	23.18162	22.64861	30.4515	25.21486	25.72471	26.48974	30.98834	25.05196	27.03664	27.22692	+	+	+	1.940164498	4.336580276	2.11498813	4.942343235	P30009;F1LMW7
Cytoskeleton Related	Mtpn	myotrophin; leucine zipper protein 6	22.70867	22.45203	23.2858	22.56772	29.00865	28.13149	28.91117	28.8595	29.2738	29.19524	29.0071	29.26155	+	+	+	6.222928311	5.97414732	7.286527503	6.430867672	P62775
Cytoskeleton Related	Nckap1	NCK-associated protein 1	23.06703	27.75786	27.55947	27.03674	30.71555	29.97131	30.21523	30.09886	30.53853	30.35584	30.43032	30.37005	+	+	+	1.882529705	3.894963741	1.982755302	4.068408966	P55161;F1LSM5
Cytoskeleton Related	Nckipsd	NCK interacting protein with SH3 domai	23.24931	22.23516	22.44091	22.70846	25.81502	26.37425	25.929	24.3058	26.17141	25.92601	25.71733	25.5099	+	+	+	2.972990558	2.947555065	4.722379584	3.172702789	D3ZWX4
Cytoskeleton Related	Plec	similar to Plectin 1 (PLTN) (PCN) (Hemid	21.31449	29.82857	28.74826	27.72234	30.89786	31.84307	31.16003	31.12201	31.4353	32.31311	31.09402	31.4345	+	+	+	1.192211671	4.352327347	1.283944939	4.665817261	427;P30427-4;P30427-
Cytoskeleton Related	Rdx	radixin	22.58775	22.51637	22.77722	22.1757	27.60081	26.54659	26.14165	26.59007	27.52522	26.89365	27.1036	27.03349	+	+	+	4.806194866	4.205515862	6.568369393	4.62473011	E9PT65
Cytoskeleton Related	Srcin1	hypothetical LOC100132856; hypotheti	22.37051	21.408	22.71915	23.68715	29.97104	29.35595	29.97596	29.92931	30.06252	29.88635	30.39008	30.15798	+	+	+	5.204352105	7.261861801	5.376025096	7.578029633	Q9QXY2;Q9QXY2-2
Cytoskeleton Related	Stmn1	stathmin 1	21.80733	24.50158	23.2323	22.28566	29.20504	28.90165	29.22702	28.40499	29.27963	28.73357	29.17088	28.91399	+	+	+	4.130721666	5.977955341	4.237415456	6.067800999	P13668;D3Z8V0
Cytoskeleton Related	Synpo	synaptopodin	23.09225	21.36888	21.9868	22.61611	28.08699	27.52298	28.00871	27.21679	28.13104	28.19385	26.74906	27.10159	+	+	+	4.838880635	5.442854881	4.259650029	5.277877331	Q9Z327;Q9Z327-3;Q9Z327-2
Cytoskeleton Related	Tagln3	transgelin 3	22.26976	22.82044	25.30671	27.2589	30.21997	29.89605	30.07661	29.91028	29.7271	30.30943	30.04166	30.03682	+	+	+	2.542750993	5.611774445	2.537314009	5.614800453	P37805
Cytoskeleton Related	Tln1	talin 1	22.76949	21.58399	22.50567	27.06866	26.40662	27.3887	26.78194	26.65335	27.1057	26.49445	27.01244	27.07521	+	+	+	1.438478483	3.325702667	1.503564289	3.439996719	G3V852
Cytoskeleton Related	Tmod2	tropomodulin 2 (neuronal)	22.12396	22.74718	22.41497	22.51693	28.3482	28.25255	27.52156	27.69876	27.80815	28.14482	27.61604	28.22995	+	+	+	6.335901239	5.504509449	6.903638976	5.49898243	P70566
Cytoskeleton Related	Tpm1	tropomyosin 1 (alpha)	22.10274	22.92953	22.81598	21.72595	27.31553	26.62199	27.01009	27.16375	27.57427	27.00796	27.38714	27.53066	+	+	+	5.133733404	4.634290695	5.387635137	4.981458187	4;P04692-
Cytoskeleton Related	Tpm3	tropomyosin 3	22.90198	22.82192	25.98848	22.12937	29.22959	27.26204	28.86623	28.52339	29.49968	27.24409	28.86118	28.30195	+	+	+	2.701700163	5.009873867	2.651763896	5.01628685	Q63610-3
Cytoskeleton Related	Tppp3	tubulin polymerization-promoting prote	21.00392	22.5796	21.93827	22.637	25.96748	24.15143	25.49503	23.29193	26.33415	24.87718	25.60425	24.7886	+	+	+	2.007834355	2.686772346	3.169931711	3.361348629	Q5PPN5
Cytoskeleton Related	Tpt1	similar to tumor protein, translationally	23.01418	21.85371	21.92995	24.21784	28.3697	27.58355	28.19817	27.82414	28.01585	27.70748	28.18806	27.81781	+	+	+	3.973932455	5.23996973	4.02054521	5.178379059	P63029;M0R8Q2
Cytoskeleton Related	Trio	triple functional domain (PTPRF interact	22.6133	22.57716	22.6756	21.9797	27.95532	28.68789	28.19938	27.0876	28.49396	27.63734	28.03312	28.52679	+	+	+	5.230262233	5.521109581	6.17659569	5.711363792	F1M0Z1;D3ZFR5
Cytoskeleton Related	Twf1	twinfilin, actin-binding protein, homolo	23.80933	24.33097	21.97564	21.81481	25.41371	26.12412	25.20322	25.26202	26.02282	25.2959	25.74563	25.99397	+	+	+	2.020022963	2.518078327	2.254085626	2.781893253	Q5RJR2
Cytoskeleton Related	Wasf1	WAS protein family, member 1	21.83373	22.98554	22.13192	21.3774	28.8814	29.08891	29.12127	28.66712	28.95258	29.33251	29.76327	28.94913	+	+	+	5.906551107	6.857526302	5.775776914	7.1672225	Q5BJU7
Endocytosis	Ap1m1	adaptor-related protein complex 3, delt	22.46224	22.90871	23.63506	22.23179	26.27142	28.41925	27.54481	28.333	25.86415	27.12633	26.99367	26.7179	+	+	+	3.766528494	4.832666397	4.036654951	3.866059303	Q32Q06
Endocytosis	Ap3d1	adaptor-related protein complex 3, delt	22.71187	21.87068	25.25018	23.02837	27.69352	28.74785	29.39419	28.99213	27.70517	28.29053	28.51367	28.34206	+	+	+	3.306109659	5.491649151	3.282436968	4.997584343	B5DFK6
Endocytosis	Clta	clathrin, light chain (Lca)	22.92755	22.77092	22.98264	22.3432	29.78706	23.42342	28.05652	27.29185	29.88686	24.16076	25.20725	24.31209	+	+	+	1.753639615	4.38363266	1.218010444	3.135659695	P08081
Endocytosis	Cltb	clathrin, light chain (Lcb)	22.92995	22.19736	21.93266	22.75237	30.16255	29.42748	28.83422	29.70086	29.76064	27.75449	27.87144	28.70141	+	+	+	5.931965843	7.078192711	4.627495676	6.068909645	P08082
Endocytosis	Cltc	clathrin, heavy chain (Hc)	26.86821	32.22268	32.18734	31.28516	34.58424	36.21212	35.53852	35.55199	34.52262	34.64705	35.26389	34.80386	+	+	+	1.976328976	4.830874443	1.75263284	4.168509483	F1M779;P11442
Endocytosis	Epn1	epsin 1	23.70482	23.22488	22.41615	23.45007	28.75643	29.30798	27.81568	27.87602	28.09396	29.05851	28.41195	28.0802	+	+	+	4.584328708	5.24004364	5.161932508	5.212172985	O88339
Endocytosis	Epn2	epsin 2	22.51694	21.68354	22.87663	22.01637	25.80565	26.40263	25.80912	25.80472	25.96847	22.94458	25.82367	25.35787	+	+	+	4.721765304	3.682156563	1.971607288	2.750272751	Q5O5I9;F1LQ45;Q9Z1Z3
Endocytosis	Itsn2	intersectin 2	23.37419	22.92972	21.60686	21.69295	26.22122	26.40105	26.96235	26.90449	26.53127	26.74804	26.55621	26.55672	+	+	+	3.921685998	4.221343517	4.086134655	4.197130203	M0R7A6;M0RAL8
Endocytosis	Lrp1	low density lipoprotein-related protein	31.39069	29.93312	26.99529	22.75744	31.82839	31.30024	31.57538	31.69183	32.07256	31.44638	31.81134	31.12454	+	+	+	1.038842793	3.829826355	1.038445534	3.84457159	G3V928
Endocytosis	Necap1	NECAP endocytosis associated 1	22.79644	22.73129	22.79847	26.64031	27.70048	27.31752	28.31264	28.28776	28.11118	27.46933	28.55146	28.58064	+	+	+	2.235285347	4.162971497	2.356083359	4.436527252	P69682
Endocytosis	Pacsin1	protein kinase C and casein kinase subs	22.21718	20.26294	24.49106	21.34362	28.74471	28.38257	29.56087	29.09484	28.76756	29.03525	29.41766	30.1939	+	+	+	3.493267095	6.867049694	3.586393321	7.274891853	F1LPP3;Q9Z0W5
Endocytosis	Picalm	phosphatidylinositol binding clathrin as	22.78313	22.97025	22.29431	22.79058	25.32316	27.53051	27.66797	27.23745	26.06281	25.94664	26.34349	26.0731	+	+	+	3.531728874	4.230207443	6.027975813	3.396942616	O55012;E9PTD2;O55012-2
Endocytosis	Sgip1	SH3-domain GRB2-like (endophilin) inte	23.85508	22.9398	26.98815	21.59607	28.26874	27.78743	28.84071	28.7346	28.31151	28.09527	28.20625	29.24617	+	+	+	2.096147725	4.563098431	2.112188552	4.620026112	F05;D4A7H8;D3ZBD8
Endocytosis	Sh3gl3	SH3-domain GRB2-like 3	22.7455	25.98765	28.2878	26.7213	22.76013	22.53669	22.86032	22.31136	21.96662	22.91703	22.62899	24.46526	+	+	+	1.523028819	-3.31843948	1.211627593	-2.941091537	O35180;F1M8F8
Endocytosis	Synj1	synaptojanin 1	22.73667	21.99566	26.09467	26.37773	29.43141	31.23329	30.22803	30.20432	29.99051	30.62532	30.40507	30.55773	+	+	+	2.622898463	5.973082542	2.760571786	6.093477726	4;Q62910-
ER/Golgi	Acbd3	acyl-Coenzyme A binding domain conta	22.14003	22.60892	22.23666	23.30216	25.34204	25.71269	25.8328	25.58164	25.51217	25.52937	26.04849	25.34871	+	+	+	2.902151911	3.506127357	2.788606834	3.528533459	G3V6E4;Q7TNY6
ER/Golgi	Aco1	aconitase 1, soluble	23.38181	22.00518	22.15998	21.25307	26.43477	26.17714	24.5044	25.70823	26.49268	26.53202	25.32406	24.56541	+	+	+	3.620616974	4.992073059	4.170518533	5.41990757	G3V6S2;Q63270
ER/Golgi	Acsbg1	acyl-CoA synthetase bubblegum family	22.41548	23.2666	20.87912	22.99087	27.38377	27.36466	27.86756	27.90438	27.64389	27.44134	28.1249	28.02158	+	+	+	7.254418239	7.451755524	7.459743612	7.373700142	Q924N5
ER/Golgi	Armc10	armadillo repeat containing 10	22.71504	21.88318	21.89339	21.96806	29.83605	29.58767	29.30252	29.54045	29.51674	29.40007	29.39656	29.6411	+	+	+	3.277168379	3.728060246	3.37222816	3.642284393	B1WBW4
ER/Golgi	Atp8a1	ATPase, aminophospholipid transporter	21.4585	20.80271	21.53392	23.17652	25.42387	26.09007	25.38617	24.98377	25.21056	25.73941	25.38367	25.20713	+	+	+	4.456621279	5.822838783	4.556387073	6.00756073	F1M439;F1LUT4;F1M585
ER/Golgi	Atp9a	ATPase, class II, type 9A	23.00936	24.1261	21.57792	22.91252	28.76613	28.41103	28.91966	28.82043	28.87713	28.76477	28.85281	29.16142	+	+	+	4.937246566	3.72206974	5.216210767	3.598638535	M0R6E0;M0R874
ER/Golgi	Bcap31	B-cell receptor-associated protein 31	21.61144	22.39846	21.79765	22.3479	26.35807	25.36842	25.48459	25.61866	25.68937	26.02357	25.4179	25.41918	+	+	+	4.682345736	4.388093472	2.469386148	3.036437988	Q6AY58
ER/Golgi	Calr	calreticulin	21.3737	22.71957	22.02681	22.50602	26.81556	26.82978	26.61246	25.92068	25.70182	26.22672	23.5662	25.27711	+	+	+	6.859614705	6.107497692	5.80220183	6.72474575	P18418
ER/Golgi	Calu	calumenin	22.84864	22.49578	23.21817	23.20248	29.07048	29.00244	29.39067	28.73146	30.23364	28.7898	30.02697	29.61365	+	+	+	1.55242979	3.011320114	2.424434472	2.934744835	G3V6S3;Q3MID6;O35783
ER/Golgi	Canx	calnexin	22.57108	21.97512	21.91155	21.25315	26.41356	22.07265	24.97128	26.29869	26.15775	23.99785	25.52283	23.77146	+	+	+	2.186971641	4.506828308	2.12944074	4.302945614	P35565

Supplementary Table 2. List of proteins significantly enriched in both immunoisolated FEZ1 and Kinesin-1 containing vesicles by quantitative mass spectrometry

Group	Gene Symbol	Gene Name	log LFQ intensity IP1 Control	log LFQ intensity IP2 Control	log LFQ intensity IP3 Control	log LFQ intensity IP4 Control	log LFQ intensity IP1 Kinesin-1	log LFQ intensity IP2 Kinesin-1	log LFQ intensity IP3 Kinesin-1	log LFQ intensity IP4 Kinesin-1	log LFQ intensity IP1 FEZ1	log LFQ intensity IP2 FEZ1	log LFQ intensity IP3 FEZ1	log LFQ intensity IP4 FEZ1	t-test Significant Kinesin-1 IP	t-test Significant FEZ1 IP	t-test Significant Kinesin-1 and FEZ1 IP	-Log t-test p value Kinesin-1 IP	t-test Difference Kinesin-1 IP	-Log t-test p value FEZ1 IP	t-test Difference FEZ1 IP	Majority UniProt protein IDs
ER/Golgi	Cdipt	CDP-diacylglycerol--inositol 3-phosphati	23.58639	28.1062	27.21642	27.84805	31.19531	30.29581	31.35584	31.9374	31.21859	30.22539	31.03597	31.48889	+	+	+	3.140391635	5.236557484	2.838571407	4.897213936	P70500
ER/Golgi	Cisd2	CDGSH iron sulfur domain 2	22.59345	21.96865	25.33344	21.85563	28.27338	28.52182	27.93556	27.96664	27.56776	28.81528	27.25611	27.70088	+	+	+	3.976974922	5.928967476	4.092614427	5.996705532	D4AAE9
ER/Golgi	Copa	coatomer protein complex, subunit alph	23.28988	21.68345	22.58467	24.68034	28.83225	29.47011	28.5996	29.05225	28.86606	29.15311	29.06974	29.13626	+	+	+	3.829481188	5.135103703	4.161275642	5.201127052	G3V6T1
ER/Golgi	Copb1	coatomer protein complex, subunit beta	23.65022	22.54915	22.10564	21.92782	26.53441	28.81985	27.66519	27.75379	26.6803	28.37977	27.9531	28.02417	+	+	+	3.451597057	4.715040684	4.331968506	4.50640583	P23514
ER/Golgi	Copb2	coatomer protein complex, subunit beta	22.50095	21.86998	23.03686	22.33154	25.60077	28.51024	26.98913	27.49937	26.03601	27.67128	26.7179	27.33977	+	+	+	3.493759331	4.06891346	4.267851894	4.164284706	O35142
ER/Golgi	Copg2	coatomer protein complex, subunit gam	21.82087	22.89474	23.37357	22.34741	25.85503	27.91279	26.5705	26.37393	26.22411	27.37551	26.81274	26.68137	+	+	+	4.701069722	3.855841637	4.304402195	3.833647251	D4ABY2
ER/Golgi	Cpd	carboxypeptidase D	22.82479	23.03706	22.90839	22.1906	25.8646	26.77178	26.66844	27.07938	25.65001	27.10169	26.64072	26.903	+	+	+	3.501515822	4.249480247	3.162443137	4.112740517	F1LPC6;Q9JHW1;Q9JHW1-2
ER/Golgi	Emc1	ER membrane protein complex subunit	23.99862	21.43463	22.20519	21.77056	26.53484	26.79761	26.58377	26.49071	26.53899	26.89966	26.81287	25.60846	+	+	+	4.599990497	5.493805408	4.697382588	5.348166943	D4A994
ER/Golgi	Emc2	ER membrane protein complex subunit	23.41532	22.48123	21.70383	23.21215	27.45732	28.71516	28.14501	28.47026	27.66397	28.58814	27.7415	28.2116	+	+	+	3.11573939	3.23371172	3.138218012	3.188257217	B0BNG0
ER/Golgi	Emc3	ER membrane protein complex subunit	23.218	21.64158	23.83541	23.51431	25.94624	26.7506	26.29686	26.15045	26.33334	26.43925	25.86022	26.32951	+	+	+	3.62047287	3.355736732	3.853640011	3.058777809	Q5U2V8
ER/Golgi	Ergic1	endoplasmic reticulum-golgi intermedia	22.47677	22.33063	21.99483	23.56864	25.45604	26.70253	25.85353	25.78172	25.35037	25.64801	25.85522	25.75239	+	+	+	1.915411012	3.368935108	1.975274513	3.536268711	F1LU48
ER/Golgi	Erp29	endoplasmic reticulum protein 29	22.24578	23.11005	26.47829	23.0459	27.46279	26.82808	26.93865	27.12623	27.58003	27.40998	26.59619	27.43889	+	+	+	2.278685532	4.969020844	2.268915433	4.9814744	P52555
ER/Golgi	Erp44	endoplasmic reticulum protein 44	22.61784	25.33923	26.42052	21.48438	28.7986	29.45262	28.79517	28.69166	28.6052	29.57306	28.63862	28.97098	+	+	+	1.390550733	3.086922169	1.475362818	3.091364861	Q5VLR5
ER/Golgi	Esd	esterase D/formylglutathione hydrolase	22.75785	22.9128	21.12986	22.41897	27.1278	22.34409	26.99378	25.10149	27.07419	26.09632	26.09225	22.32218	+	+	+	3.914618703	3.956925869	4.857849143	4.176487446	B0BNE5
ER/Golgi	Glg1	golgi apparatus protein 1	22.11024	23.09553	23.26136	22.90114	25.9018	26.47146	27.43492	27.3878	26.98642	26.46183	27.23818	27.3878	+	+	+	1.592567679	4.09209919	1.679709803	4.332531929	G3V8G5;Q62638
ER/Golgi	Golga7	golgi autoantigen, golgin subfamily a, 7	28.20802	22.31198	22.62392	24.15628	29.25579	28.18806	28.29667	27.92807	29.26307	28.91795	28.70415	27.74516	+	+	+	3.878490551	3.00494051	3.654824584	2.986231804	Q6AYQ1
ER/Golgi	Golgb1	golgin B1, golgi integral membrane prot	22.30299	22.96401	22.49819	23.47536	25.93118	26.33424	25.22777	25.76711	25.70559	26.23186	25.05577	26.19225	+	+	+	1.645069344	4.950472832	3.013326699	6.025657177	G3V6A8
ER/Golgi	Hdhd2	haloacid dehalogenase-like hydrolase do	23.01709	22.03924	22.16632	21.35725	30.97658	27.78675	26.27895	23.33951	30.69437	28.44884	27.19573	26.34359	+	+	+	4.591297532	4.111990929	4.600844698	4.227925301	Q6QI86
ER/Golgi	Hook3	hook homolog 3 (Drosophila)	21.95625	22.15456	23.0977	23.14881	26.22589	27.04479	26.8484	26.68619	26.91513	26.26372	27.14793	26.94224	+	+	+	2.655214048	-2.66128826	2.863660632	-2.809535027	F1MA65
ER/Golgi	Hsd17b12	hydroxysteroid (17-beta) dehydrogenas	26.24056	26.34228	27.42694	26.44351	23.38045	25.18427	23.23735	24.00607	24.72588	23.11837	24.30145	23.06945	+	+	+	2.649570885	4.766582966	2.775167595	5.018529892	Q6P7R8;D3ZPU3
ER/Golgi	Kdelr1	KDEL (Lys-Asp-Glu-Leu) endoplasmic ret	23.09823	19.78081	23.73443	22.91559	26.23118	27.4235	27.4996	27.44111	26.61133	27.32225	27.82699	27.8426	+	+	+	1.91242297	3.752366066	2.00964325	3.867630005	Q569A6
ER/Golgi	Lman2	lectin, mannose-binding 2	26.6513	22.20254	23.27025	22.38119	28.03302	27.10819	27.01201	27.36152	27.30591	27.57766	27.65922	27.43301	+	+	+	5.337644491	4.199424267	5.764159184	4.515965462	B0BNG3
ER/Golgi	Lrrc59	leucine rich repeat containing 59	23.24384	22.68006	22.28106	22.70105	27.06312	27.37202	26.74881	26.51976	27.43865	27.51706	27.14501	26.86915	+	+	+	5.502328843	4.577185154	4.935832922	4.493526936	Q5RJR8
ER/Golgi	Manf	mesencephalic astrocyte-derived neuro	21.96126	22.64659	23.11111	22.07175	26.81029	27.06979	27.14141	27.07796	27.47491	26.73092	26.50724	27.05175	+	+	+	3.356179831	3.485677242	2.049529928	2.826660156	P0C5H9
ER/Golgi	Mgst3	microsomal glutathione S-transferase 3	21.60422	22.81615	22.47185	23.64046	26.58477	26.36998	25.34282	26.1778	26.5819	23.72614	25.90713	25.62414	+	+	+	1.834690132	4.960216045	1.839458189	4.973721027	D4ADS4;D3ZZQ8
ER/Golgi	Mlec	malectin	22.70242	22.44511	27.10879	27.98685	29.61261	30.4716	29.75073	30.24909	29.48865	30.32002	30.37254	29.95685	+	+	+	3.490030909	3.363307476	3.373721328	3.313305378	Q5FVQ4
ER/Golgi	Mmgt1	membrane magnesium transporter 1	22.68912	22.75814	23.3056	24.46534	26.45803	27.21076	26.71437	26.28828	26.30248	27.30895	26.51605	26.34394	+	+	+	1.411000549	3.209012508	3.401622217	4.433445454	B5DF51
ER/Golgi	Nsfl1c	NSFL1 (p97) cofactor (p47)	23.10292	22.87916	20.97153	22.55284	26.28258	28.0403	25.34001	22.67962	27.34528	27.64203	26.11698	26.13594	+	+	+	4.102249211	4.746310234	4.417610947	4.854521751	O35987
ER/Golgi	Pacs1	phosphofurin acidic cluster sorting prot	22.78238	22.3972	21.8933	20.8102	26.84566	26.9776	27.09778	25.94728	27.01223	26.77052	27.07979	26.43863	+	+	+	1.325918563	3.731907845	1.696336303	4.576165199	F1LPG3;O88588
ER/Golgi	Pdia3	protein disulfide isomerase family A, me	21.52192	20.74511	26.21573	26.08817	27.56012	27.91986	27.67263	26.34594	28.2736	28.08917	28.30326	28.20955	+	+	+	6.659327577	6.440283775	6.88668587	6.550332069	P11598
ER/Golgi	Pdia4	protein disulfide isomerase family A, me	23.128	22.56275	22.12103	22.568	29.0855	28.62965	29.24518	29.18059	28.92292	29.22046	29.03438	29.40335	+	+	+	1.256175164	3.67414093	3.094251409	4.972755909	G3V6T7;P38659
ER/Golgi	Pdia6	protein disulfide isomerase family A, me	23.00416	22.10857	19.93806	22.72238	28.20359	21.70485	26.0002	26.5611	28.0926	26.63118	26.63159	26.30883	+	+	+	5.01484157	4.364637852	6.453495444	4.784714222	Q63081
ER/Golgi	Pitpnm1	similar to Pyruvate kinase, isozymes M1	23.41637	23.24949	23.68279	23.31083	28.15967	27.06394	27.49618	28.39824	28.28299	27.8208	28.64454	28.04999	+	+	+	2.572532738	2.880156994	2.977736908	3.239315987	Q5U2N3
ER/Golgi	Ppib	peptidylprolyl isomerase B (cyclophilin B	23.1032	25.08383	25.53828	24.73242	27.17026	28.22986	27.39485	27.18338	27.8962	28.21405	27.52567	27.77906	+	+	+	5.758480504	6.749500751	5.869478536	6.658109188	P24368
ER/Golgi	Praf2	PRA1 domain family, member 2	22.48396	21.65278	23.23407	22.07566	29.51296	28.94226	28.81568	29.17358	29.23423	29.02374	29.04234	28.77859	+	+	+	4.197019232	4.849228382	5.024691537	4.243733406	D3ZAA0
ER/Golgi	Prkcsh	protein kinase C substrate 80K-H	22.35	22.20963	22.96762	23.22221	27.2922	28.73399	27.42109	26.69909	27.14433	27.36277	26.6679	26.54938	+	+	+	3.426607226	4.76993084	3.849980592	5.330377579	B1WC34
ER/Golgi	Rcn2	reticulocalbin 2, EF-hand calcium bindin	24.28269	21.44664	22.56391	22.086	28.15282	26.88577	27.21558	27.20481	28.33611	27.75423	27.90478	27.70563	+	+	+	2.625877594	3.290500164	3.064804821	3.311664104	Q62703
ER/Golgi	Rdh11	retinol dehydrogenase 11 (all-trans/9-ci	24.77993	23.66892	22.44477	23.35856	25.59309	26.89493	27.41046	27.51571	26.16457	27.05734	26.99583	27.28109	+	+	+	2.409497765	2.996777058	3.977543707	3.70250988	Q6TUD3;Q6AXX5
ER/Golgi	Rrbp1	ribosome binding protein 1 homolog 18	21.62516	22.50693	22.37647	21.96739	26.31363	23.3794	25.63879	25.13124	26.71004	25.88623	25.7318	24.95793	+	+	+	2.418219072	4.964787006	2.423148581	4.983423233	F1M853;F1M5X1
ER/Golgi	Rtn1	reticulon 1	23.49556	23.09543	21.9557	22.89763	26.4303	25.85619	30.34678	28.6702	26.55979	25.6299	29.95269	29.23565	+	+	+	2.781705957	3.174314499	2.882058598	3.314896584	Q64548
ER/Golgi	Rtn1	reticulon 1	28.00244	26.65663	29.14022	29.01236	31.12885	31.2883	31.69524	31.39652	31.42122	31.32088	31.4827	31.84644	+	+	+	1.643363221	4.413711071	1.636425191	4.421675205	Q64548-2
ER/Golgi	Rtn3	reticulon 3	22.72079	22.66025	27.62659	27.75653	29.19643	29.72329	29.67314	29.82614	29.07125	29.55852	29.7366	30.08449	+	+	+	2.507719356	2.438488483	2.772859897	2.786403179	Q6RJR6
ER/Golgi	Rtn4	reticulon 4	29.2994	27.01755	27.78874	27.71646	30.84916	30.45307	30.25417	30.01969	31.18364	30.94224	30.40639	30.43549	+	+	+	7.508059832	8.271906853	7.856862129	8.528227329	F1LQN3;Q9JK11
ER/Golgi	Rtn4	reticulon 4	22.25771	23.06244	22.17633	22.5885	30.95116	31.01651	30.64083	30.56411	30.93937	31.09409	30.99577	31.16866	+	+	+	4.310365926	4.344334126	4.570669119	4.509833336	Q9JK11-3
ER/Golgi	Sec11a	SEC11 homolog A (S. cerevisiae)	22.37472	24.05524	22.5612	22.5998	27.29307	27.68251	26.96279	27.02992	27.4243	27.58749	27.30365	27.31484	+	+	+	3.960670349	3.396006584	3.157003055	2.88897419	Q6P9X2;P42667
ER/Golgi	Sec11c	SEC11 homolog C (S. cerevisiae)	21.57849	21.12952	21.91192	22.56682	25.52432	25.57022	25.09253	24.58371	24.02141	25.60213	24.4051	24.71403	+	+	+	2.268608758	3.331003666	1.678439768	2.826298714	G3V878;Q9WTR7
ER/Golgi	Sec22b	SEC22 vesicle trafficking protein homolo	19.83062	23.53382	21.48957	21.48451	25.01767	24.55192	24.65665	25.43629	22.96659	24.63001	24.74124	25.30588	+	+	+	1.307714584	2.935056686	1.251241493	2.899754047	Q4KM74
ER/Golgi	Sec23a	Sec23 homolog A (S. cerevisiae)	22.25456	26.78444	26.07372	27.14734	28.48784	29.62246	27.84062	28.04937	28.0276	29.92705	27.8773	28.02712	+	+	+	1.848394858	4.105154991	2.130614082	4.354877472	B5DFC3
ER/Golgi	Sep15	15 kDa selenoprotein	22.31606	21.29328	26.25429	22.56061	26.63755	28.57838	27.3964	26.23253	27.4219	27.95588	27.25187	27.2141	+	+	+	4.412892508	3.045354366	4.234023509	3.037744999	Q923V8
ER/Golgi	Ssr4	signal sequence receptor, delta (translo	22.19005	21.59034	23.07509	22.00115	27.36227	28.17255	27.55318	27.51156	27.11595	27.93219	26.78768	27.00774	+	+	+	5.26955433	5.435733318	4.789567311	4.996732235	Q07984
ER/Golgi	Tecr	glycoprotein, synaptic 2	23.17777	22.64777	23.44498	22.91184	27.6224	27.55201	26.96763	27.26964	27.44411	27.36553	26.78494	27.07602	+	+	+	5.853604894	4.307329655	5.734977304	4.122060776	Q64232
ER/Golgi	Tmed10	transmembrane emp24-like trafficking p	28.61182	21.10599	27.52649	22.71459	29.82443	29.16813	27.84631	27.96587	28.88922	29.41814	28.33296	28.65901	+	+	+	1.01652988	3.711462498	1.087789106	3.835109711	Q63584
ER/Golgi	Tmed9	transmembrane emp24 protein transpo	22.45451	23.47967	22.00148	23.16012	28.32689	27.30217	26.06885	25.37302	27.85946	27.49244	26.77555	25.94036	+	+	+	2.790371068	3.993786335	3.648240711	4.243008137	Q5I0E7
ER/Golgi	Tmx1	thioredoxin-related transmembrane pro	22.34138	24.54609	23.10289	21.76409	27.1032	26.92909	27.07938	26.97771	27.62415	26.98967	27.01084	27.09073	+	+	+	3.294348699	4.083731174	3.317361535	4.240234375	Q52KJ9
ER/Golgi	Trappc2l	trafficking protein particle complex 2-lik	21.33229	21.53891	22.46166	22.54322	24.89023	25.80659	25.95859	25.92121	21.99081	25.67898	24.99187	26.18386	+	+	+	4.019894505	3.675135136	1.490122032	2.742363453	B2RYU6
ER/Golgi	Trappc3	trafficking protein particle complex 3	21.52069	22.42236	22.2014	21.9551	26.92048	27.43038	25.90793	26.21484	25.87636	27.27524	25.81556	26.96069	+	+	+	4.617455996	4.593520164	4.384268046	4.457073212	Q5U1Z2
ER/Golgi	Trappc4	trafficking protein particle complex 4	22.21282	21.38395	22.73256	23.19845	24.4419	25.28306	25.00736	25.43476	25.09043	23.02611	25.32313	25.41893	+	+	+	3.001442666	2.659821033	1.834869091	2.332703114	Q69BT7
ER/Golgi	Uggt1	UDP-glucose ceramide glucosyltransfera	23.2571	21.0131	21.2339	22.70679	27.99841	24.64213	28.24245	27.94112	28.22417	26.49816	28.8365	28.25876	+	+	+	2.636350052	5.153307915	3.661796858	5.901677132	Q9JLA3
ER/Golgi	Vcp	valosin-containing protein	20.96077	22.85605	21.82039	22.4847	30.26394	30.02446	30.69793	30.92103	30.05232	30.25192	30.55335	30.81955	+	+	+	5.757582445	8.446362972	5.819939824	8.38880682	P46462
ER/Golgi	Wfs1	Wolfram syndrome 1 (wolframin)	22.36161	22.44486	21.88499	23.02539	27.94743	28.76689	27.56732	27.61034	28.08639	28.4937	27.78032	27.67828	+	+	+	5.301282002	5.543786049	5.832785259	5.580459118	E9PT53
Exocytosis	Cadps	Ca++-dependent secretion activator	25.84252	24.4276	22.02968	25.75251	29.77844	30.25372	29.77983	29.15398	29.92606	29.70137	30.03944	30.41609	+	+	+	2.900440574	5.228414059	3.057743826	5.507664204	Q62717

Supplementary Table 2. List of proteins significantly enriched in both immunoisolated FEZ1 and Kinesin-1 containing vesicles by quantitative mass spectrometry

Group	Gene Symbol	Gene Name	log LFQ intensity IP1 Control	log LFQ intensity IP2 Control	log LFQ intensity IP3 Control	log LFQ intensity IP4 Control	log LFQ intensity IP1 Kinesin-1	log LFQ intensity IP2 Kinesin-1	log LFQ intensity IP3 Kinesin-1	log LFQ intensity IP4 Kinesin-1	log LFQ intensity IP1 FEZ1	log LFQ intensity IP2 FEZ1	log LFQ intensity IP3 FEZ1	log LFQ intensity IP4 FEZ1	t-test Significant Kinesin-1 IP	t-test Significant FEZ1 IP	t-test Significant Kinesin-1 and FEZ1 IP	-Log t-test p value Kinesin-1 IP	t-test Difference Kinesin-1 IP	-Log t-test p value FEZ1 IP	t-test Difference FEZ1 IP	Majority UniProt protein IDs
Exocytosis	Cadps2	Ca++-dependent secretion activator 2	22.76503	21.43411	23.11902	23.2621	24.4222	25.49054	25.32776	25.75906	24.76051	25.23403	25.64834	26.37817	+	+	+	2.665943969	2.604826927	2.73694756	2.860198975	1M068;F1LYK8;F1M5U6
Exocytosis	Cplx1	complexin 1	32.01442	31.42437	31.87104	31.16297	28.64743	28.08172	28.67976	28.35018	28.724	27.5564	28.77584	28.10934	+	+	+	4.920265844	-3.17842627	4.119454786	-3.326806068	P63041
Exocytosis	Scrn1	secernin 1	22.72238	23.07015	22.95705	23.13685	27.40007	26.04652	26.52046	27.55874	27.41224	23.776	27.02865	27.52604	+	+	+	4.365355143	3.909842968	2.077412358	3.464127541	Q6AY84
Exocytosis	Syt7	synaptotagmin XVII; synaptotagmin VII	22.46331	22.96113	23.02452	22.33475	26.49248	26.91319	27.3093	26.83062	26.83038	26.58878	28.27226	27.73402	+	+	+	5.627728694	4.190474033	4.442111278	4.660435677	9P37;Q99P35;Q62747
GTPase related	Abr	active BCR-related gene	22.78667	22.34558	26.92863	23.58166	27.10029	28.03779	27.42005	27.46209	27.8773	27.42638	27.57982	27.96124	+	+	+	1.840494899	3.594419479	1.96259541	3.800550938	D4A6K9
GTPase related	Adap1	ArfGAP with dual PH domains 1	21.68694	21.65207	22.22252	22.79057	27.43388	22.98665	27.22555	27.69392	26.92965	26.373	26.81005	27.16135	+	+	+	1.989516754	4.246975422	5.259873925	4.730488777	O88768
GTPase related	Agap2	ArfGAP with GTPase domain, ankyrin re	22.34326	22.67036	26.48468	23.51228	27.07979	25.65116	27.96983	27.22803	29.0553	26.38614	27.4377	27.89157	+	+	+	1.644935811	3.229553699	1.946076057	3.940029144	Q8CGU4
GTPase related	Arfgef1	ADP-ribosylation factor guanine nucleot	24.34411	22.64584	22.63262	21.89743	25.38166	26.58764	25.80053	24.9444	26.2438	25.48778	24.30201	26.8282	+	+	+	2.375130593	2.798556805	2.031461716	2.835449219	D4A631
GTPase related	Arfgef2	ADP-ribosylation factor guanine nucleot	22.79747	22.01749	21.92274	22.23568	26.22402	27.4817	27.21947	27.1025	27.42718	26.796	26.3146	28.03428	+	+	+	5.114910779	4.763575077	4.60615375	4.899671078	Q7TSU1
GTPase related	Arhgap26	Rho GTPase activating protein 26	22.35069	23.46957	22.57655	21.9804	25.92361	25.6551	25.9885	26.01761	26.37021	26.10051	25.7313	25.93434	+	+	+	4.25965458	3.301901817	4.235604869	3.439788342	D3ZMS4
GTPase related	Arhgap32	Rho GTPase-activating protein	21.88028	21.39506	22.53874	22.27006	26.11032	22.00385	26.43687	25.5024	26.62798	22.72717	25.12857	26.51779	+	+	+	1.532489959	2.99232626	1.855608296	3.229341507	F1MAK3
GTPase related	Arhgap35	Rho GTPase activating protein 35	22.99814	22.00443	23.21329	21.36878	24.53362	26.24009	25.48351	25.42861	24.57715	26.18206	26.20126	25.37113	+	+	+	2.798198237	3.025299072	2.817353743	3.186739445	D4AD82;P81128
GTPase related	Arhgdia	Rho GDP dissociation inhibitor (GDI) alp	22.49603	27.49358	27.9997	22.67239	30.83425	31.28074	30.93761	30.82941	30.67497	31.03564	30.80947	30.91242	+	+	+	2.085261145	5.805077076	2.049689472	5.692699432	Q5XI73
GTPase related	Arl1	ADP-ribosylation factor-like 1	30.40993	29.30572	30.7925	30.38484	27.00507	28.31523	27.45136	27.95571	27.29772	28.63145	26.38568	27.06671	+	+	+	2.983755296	-2.54140472	2.636221584	-2.877857208	P61212
GTPase related	Asap1	ArfGAP with SH3 domain, ankyrin repea	21.50005	21.98051	23.29321	21.46809	24.74883	25.00423	25.20743	23.02351	25.50694	24.82236	25.26607	25.55456	+	+	+	1.998374072	2.435538769	3.383826877	3.227016449	2;Q1AAU6-
GTPase related	Dmxl2	Dmx-like 2	25.76218	28.95435	30.17379	28.5192	31.08967	31.59197	31.54398	31.11942	30.95234	31.62575	31.93403	31.60744	+	+	+	1.71393962	2.983878613	1.800926235	3.177511215	F1M164;F1M3W5
GTPase related	Dock3	dedicator of cytokinesis 3	21.69927	22.49897	23.0747	22.07312	24.81695	25.12278	26.01801	25.38021	26.20288	25.33933	24.64515	26.06517	+	+	+	3.593339041	2.997971058	3.349755937	3.226620674	F1M4N6
GTPase related	Dock4	dedicator of cytokinesis 4	23.76031	22.37794	22.63307	22.52261	25.22887	25.07468	25.85065	25.69062	25.88008	25.47769	25.40406	25.71926	+	+	+	3.439669726	2.637721539	3.794076179	2.796790123	F1LZ81;M0R6K4
GTPase related	Dock7	dedicator of cytokinesis 7	21.64353	23.87744	23.56221	21.50922	28.89322	28.52073	27.7107	22.511	29.11553	22.72987	28.98636	29.95005	+	+	+	1.416166064	4.260811806	1.525499522	5.047350883	F1LRS2
GTPase related	Dock9	dedicator of cytokinesis 9	22.39527	21.68881	23.0743	21.82046	25.542	22.56474	25.65272	26.05277	25.96431	22.07808	25.5461	25.73281	+	+	+	1.695347331	2.708349705	1.422350828	2.585614681	F1LSM8
GTPase related	Elmo2	engulfment and cell motility 2	23.02278	24.5314	22.56222	22.31916	25.22192	25.96928	26.39496	26.58219	25.39908	25.03128	26.67909	26.15584	+	+	+	2.632155771	2.933197021	2.326730555	2.707431793	G3V982
GTPase related	Farp1	FERM, RhoGEF (ARHGEF) and pleckstrin	22.91377	22.30193	22.26763	21.54887	27.05393	24.92275	25.94777	25.87	27.25593	26.24105	26.91308	26.34924	+	+	+	3.416535475	3.690562248	4.703219599	4.431774139	F1LYQ8
GTPase related	Gdi1	GDP dissociation inhibitor 1	22.18763	23.06901	22.99881	23.48571	29.65717	30.0427	30.75573	30.50815	30.15593	30.11235	31.08556	30.69071	+	+	+	5.994496872	7.305651188	6.144759799	7.57585001	P50398
GTPase related	Gdi2	GDP dissociation inhibitor 2	22.69543	23.63736	22.13069	23.95501	29.76178	29.50992	30.2878	30.64083	30.08132	29.99389	30.91399	30.34615	+	+	+	5.103569374	6.945457458	5.326892706	7.2292099	P50399
GTPase related	Gna11	guanine nucleotide binding protein (G p	22.43344	23.37946	21.97148	22.12355	28.01983	27.33364	28.07984	27.61555	28.5029	27.89174	28.26656	27.59249	+	+	+	5.191151404	5.285232067	5.243552965	5.586442471	G3V6Q6;Q9JID2
GTPase related	Gna13	guanine nucleotide binding protein (G p	22.39301	20.92694	22.45195	22.78419	27.81213	26.49505	26.80366	27.30173	27.57499	25.8429	27.67633	27.4479	+	+	+	4.199150335	4.964120865	3.793586493	4.996509552	F1LNG7
GTPase related	Gnai2	guanine nucleotide binding protein (G p	22.32383	23.57242	22.63829	25.93766	29.68201	30.11905	30.64985	30.43052	30.04114	30.20338	30.80908	30.7605	+	+	+	3.637808868	6.602304459	3.732090828	6.835475922	P04897
GTPase related	Gnai3	guanine nucleotide binding protein (G p	23.3413	22.52796	22.80492	23.21566	27.43269	26.22338	26.49175	25.80035	27.83129	26.14354	26.75149	26.45284	+	+	+	3.960295142	3.51458168	4.051461488	3.822328091	P08753
GTPase related	Gnal	guanine nucleotide binding protein (G p	22.8509	22.01386	24.00018	22.08176	26.74201	25.2907	25.86365	26.10877	26.37893	25.81497	25.45054	25.46067	+	+	+	2.986469021	3.26460886	2.996791525	3.039604187	G3V8E8;P38406
GTPase related	Gnaq	guanine nucleotide binding protein (G p	22.48593	22.20998	26.22144	27.46716	30.50095	30.50256	30.85542	31.14382	30.63798	30.90254	30.81092	30.64272	+	+	+	2.440622716	6.154562473	2.451718378	6.152413845	D4AE68;P82471
GTPase related	Gnas	GNAS complex locus	22.89482	23.40715	21.5779	22.75432	28.57069	27.33296	28.12829	27.91804	28.48941	28.15788	28.3878	27.30077	+	+	+	4.580811198	5.328948021	4.586489351	5.425420761	2;M0R9J4
GTPase related	Gnaz	guanine nucleotide binding protein (G p	22.91181	23.29804	22.01365	21.67304	29.34295	29.3078	30.07559	29.78712	29.73784	29.29141	30.35237	30.18009	+	+	+	5.571788526	7.154233932	5.511427464	7.416295052	P19627
GTPase related	Gnb4	guanine nucleotide binding protein (G p	22.25904	23.1025	22.99803	22.22549	28.06676	27.51871	28.63758	28.82308	28.6173	27.37294	29.13099	28.34685	+	+	+	5.23945315	5.6152668	4.910583791	5.720752239	O35353;D4A752
GTPase related	Gnb5	guanine nucleotide binding protein (G p	22.45596	21.42004	25.34218	22.59816	29.10302	28.73486	29.05972	28.68889	28.99664	28.49007	28.9803	29.25365	+	+	+	3.38393127	5.942538738	3.374485288	5.976080894	P62882;M0RAX4
GTPase related	Gpr158	G protein-coupled receptor 158	21.76673	23.17414	21.31132	20.93395	27.95188	22.99492	28.08527	27.40251	28.21729	27.19338	29.09841	28.46252	+	+	+	1.984338935	4.812108994	4.294725943	6.446365833	D4A6L0
GTPase related	Gpr37l1	G protein-coupled receptor 37 like 1	21.27617	21.63016	22.03678	21.86247	28.30839	26.81604	27.52111	28.2283	27.79712	26.7278	27.77712	27.81623	+	+	+	5.356989681	6.017065525	5.803045079	5.82817173	B4F7C1;Q9QYC5
GTPase related	Hras1	Harvey rat sarcoma virus oncogene	23.2831	22.38363	27.84972	28.44451	30.42092	29.62669	30.25056	29.54258	30.23831	29.48033	30.27242	29.8428	+	+	+	1.538174554	4.469948292	1.542079375	4.468227863	P20171
GTPase related	LOC10036	T-cell lymphoma invasion and metastasi	21.18145	22.53634	22.16768	23.14051	24.27718	25.97491	25.16783	24.84479	24.83812	25.60789	25.18903	25.09196	+	+	+	2.687152014	2.809682846	3.24830558	2.925257206	D3ZMS5
GTPase related	Mras	muscle RAS oncogene homolog	23.93201	21.84117	22.05035	23.28378	26.63934	26.96818	27.30095	27.16672	27.36945	27.02422	27.34079	27.08659	+	+	+	3.739384081	4.241969585	3.903644002	4.428432941	P97538;Q5RKJ7
GTPase related	Nf1	neurofibromin 1	24.17551	23.47301	22.82086	21.95098	28.01041	28.55614	28.49137	28.11946	28.48961	27.87074	28.24004	28.48669	+	+	+	4.36616871	5.189256668	4.339654595	5.166679859	F1LM28;P97526
GTPase related	Ngef	neuronal guanine nucleotide exchange f	22.25092	22.58259	22.18434	22.51788	27.63124	28.26955	27.99712	27.59413	28.4691	28.59292	28.37094	28.02062	+	+	+	6.971728028	5.489075184	7.657557993	5.979460239	Q5BKC9;G3V856
GTPase related	Nras	neuroblastoma RAS viral (v-ras) oncoger	22.59621	23.0853	22.13758	22.88125	26.79005	26.40667	26.76724	26.29558	26.92536	25.98257	27.17102	25.89525	+	+	+	5.447524259	3.889801025	4.222596826	3.81846714	Q04970
GTPase related	Pafah1b1	platelet-activating factor acetylhydrolas	21.71628	22.84889	24.87629	23.52073	28.55168	27.95371	28.46649	28.36553	28.15108	28.29101	28.55076	28.72227	+	+	+	3.557164305	5.093807697	3.604068191	5.188234329	P63004
GTPase related	Rab21	RAB21, member RAS oncogene family	23.20679	23.54988	22.93284	23.70109	27.183	26.43567	27.47475	27.42654	27.70306	27.70464	27.72592	27.82347	+	+	+	4.853026947	3.782343864	6.579009379	4.391624928	Q6AXT5
GTPase related	Rab23	RAB23, member RAS oncogene family	22.12575	21.95892	24.05396	22.75245	26.01129	25.60541	25.81709	26.08479	26.09901	25.70277	26.2	26.03398	+	+	+	3.191304338	3.15687418	3.284617506	3.286168575	D3ZRM5
GTPase related	Rab3b	RAB3B, member RAS oncogene family	21.96853	22.80208	25.94985	22.51532	26.55445	26.67681	26.64127	25.92635	26.19625	26.17718	26.08875	26.04496	+	+	+	1.857553981	3.140777588	1.696658132	2.817842007	Q63941
GTPase related	Rab3gap2	RAB3 GTPase activating protein subunit	22.63279	22.42851	22.42115	21.39354	25.49534	27.33697	26.36668	24.31665	26.9301	26.87572	26.6675	27.35229	+	+	+	2.706041262	3.659911633	5.270304038	4.737405777	D4ABP4;F1LMT8
GTPase related	Rabgap1	RAB GTPase activating protein 1	22.53481	23.22753	21.67474	21.89575	24.82002	27.40617	26.15163	25.97147	25.99391	27.04156	25.38242	26.51096	+	+	+	2.984496398	3.754113674	3.639205729	3.899001598	D3ZX42
GTPase related	Rac1	ras-related C3 botulinum toxin substrate	25.89379	26.83653	28.32036	28.00233	31.59237	29.75285	31.40075	31.44352	31.44919	30.49524	31.94304	31.32619	+	+	+	2.763397216	3.784116745	3.158299845	4.040160656	Q6RUV5
GTPase related	Ralgapb	KIAA1219	23.14402	21.69039	21.76923	21.89779	25.66408	22.44951	25.81868	25.46995	25.07198	23.26391	26.21126	25.81729	+	+	+	1.686635572	2.725195885	2.158174242	2.965749741	P86410
GTPase related	Ran	RAN, member RAS oncogene family	21.1903	21.6054	26.64471	23.01884	28.15899	27.99572	28.81714	28.73818	28.53297	28.01781	28.87341	29.13673	+	+	+	2.258060189	5.312696934	2.327924106	5.525416851	P62828;Q66H11
GTPase related	Ranbp1	similar to RAN binding protein 1; RAN bi	21.96573	23.02048	22.95037	23.29859	27.29334	27.47507	28.00287	27.70971	26.76116	27.25503	22.80459	28.05129	+	+	+	5.193739853	4.811452866	1.524257447	3.409223557	D4A2G9
GTPase related	RAP1A	RAP1A, member of RAS oncogene family	22.9416	20.6165	23.502	21.77091	26.77165	25.90224	26.61498	22.38905	26.78806	26.24847	26.73686	26.38019	+	+	+	1.420581076	3.21172905	3.239642259	4.33064127	P62836
GTPase related	Rap1b	RAP1B, member of RAS oncogene family	27.54473	27.71116	28.61842	22.99811	30.47557	29.56752	30.26908	29.75778	30.298	29.29933	30.35005	29.01606	+	+	+	1.377715857	3.299384594	1.220807185	3.022758007	Q62636
GTPase related	Rap1gds1	RAP1, GTP-GDP dissociation stimulator 1	21.66169	22.0204	22.3532	23.21928	27.09154	26.14209	26.9486	26.48967	26.1559	26.07592	27.29351	27.3268	+	+	+	4.461754384	4.354335785	4.023230052	4.399394035	F1M7Y3
GTPase related	Rap2a	RAP2A, member of RAS oncogene family	24.23043	23.10435	21.83848	21.57355	29.30643	28.66895	29.41353	29.25183	29.13712	28.45148	29.42113	28.89934	+	+	+	4.281658454	6.473484039	4.168130184	6.290566921	D3ZK56;Q78P65
GTPase related	Rap2b	RAP2B, member of RAS oncogene family	22.43874	22.86623	22.28714	23.6861	27.88169	27.82953	28.09688	28.17926	27.86136	28.46045	28.31609	28.32002	+	+	+	5.412067762	5.177287579	5.413753366	5.419928551	P61227
GTPase related	Rapgef4	Rap guanine nucleotide exchange factor	22.68605	23.50729	21.61111	21.14938	24.70065	25.12766	25.39087	25.51488	23.2676	25.44332	25.59414	25.70786	+	+	+	2.715915546	2.945057392	1.897628254	2.764771938	F1LQ26;Q9Z1C7
GTPase related	Rasal1	RAS protein activator like 1 (GAP1 like)	22.1974	22.76116	25.74276	21.9527	28.58592	28.57481	28.74272	28.53419	29.06296	28.60045	28.97194	28.57885	+	+	+	3.093113393	5.445907116	3.153776481	5.640048027	D3ZHY9
GTPase related	Rgs7	regulator of G-protein signaling 7	21.54885	22.07752	22.70589	22.22659	26.16887	24.15683	26.61133	25.42803	25.71978	26.53133	27.54119	27.57268	+	+	+	2.968478654	3.451550961	4.060980458	4.701533318	P49803;D3ZWG2
GTPase related	Rgs7bp	regulator of G-protein signaling 7 bindin	22.53208	22.75717	21.96968	23.71716	25.85721	26.12951	25.70203	25.85648	26.39886	26.28798	26.66844	26.79228	+	+	+	3.804238445	3.14228344	4.216591787	3.792868614	Q5FVH8

Supplementary Table 2. List of proteins significantly enriched in both immunoisolated FEZ1 and Kinesin-1 containing vesicles by quantitative mass spectrometry

Group	Gene Symbol	Gene Name	log LFQ intensity IP1 Control	log LFQ intensity IP2 Control	log LFQ intensity IP3 Control	log LFQ intensity IP4 Control	log LFQ intensity IP1 Kinesin-1	log LFQ intensity IP2 Kinesin-1	log LFQ intensity IP3 Kinesin-1	log LFQ intensity IP4 Kinesin-1	log LFQ intensity IP1 FEZ1	log LFQ intensity IP2 FEZ1	log LFQ intensity IP3 FEZ1	log LFQ intensity IP4 FEZ1	t-test Significant Kinesin-1 IP	t-test Significant FEZ1 IP	t-test Significant Kinesin-1 and FEZ1 IP	-Log t-test p value Kinesin-1 IP	t-test Difference Kinesin-1 IP	-Log t-test p value FEZ1 IP	t-test Difference FEZ1 IP	Majority UniProt protein IDs
GTPase related	Rhoa	ras homolog gene family, member A	26.98783	27.04521	29.29914	29.13868	30.66738	29.86294	31.1313	31.27542	30.94077	30.24536	31.07355	30.97056	+	+	+	1.98389326	2.616547585	2.171690934	2.689844608	P61589
GTPase related	Rhob	ras homolog gene family, member B	22.79791	21.42723	27.07806	26.50241	29.31918	28.56369	29.90412	29.70563	29.71749	28.9447	30.05724	29.74951	+	+	+	1.880040286	4.921751022	1.985364509	5.16583252	P62747
GTPase related	Rhog	ras homolog gene family, member G (rh	23.20141	22.67753	22.53785	26.79811	27.66289	27.70352	28.05517	27.84948	27.66594	28.03244	27.93337	28.2109	+	+	+	2.130581687	4.01403904	2.195133669	4.156937599	Q32PX6
GTPase related	Rock2	Rho-associated, coiled-coil containing p	20.36706	21.93573	23.15666	22.93357	27.29807	26.80464	27.29544	26.84482	27.76787	26.61372	27.13261	28.2305	+	+	+	3.580417533	4.962488651	3.484567929	5.337924004	Q62868;F1LQT3
GTPase related	Rph3a	rabphilin 3A homolog (mouse)	22.84733	27.79185	25.96107	27.50066	29.96416	29.67145	29.76778	29.82557	29.9414	29.68494	29.85983	30.25113	+	+	+	1.803069057	3.782012463	1.855777666	3.909096718	F1LPB9;P47709
GTPase related	RRAS2	related RAS viral (r-ras) oncogene homo	22.01804	22.23543	23.0278	23.05684	28.4172	27.07918	27.23654	27.42238	27.47877	27.59605	26.74945	27.04916	+	+	+	4.752403766	4.954297543	5.075427093	4.633832455	Q5BJU0
GTPase related	Sar1a	SAR1 homolog A (S. cerevisiae)	31.95985	31.53417	30.98359	31.33479	28.34109	28.38022	28.88402	28.60116	28.92417	28.81032	27.71967	22.45153	+	+	+	4.723381907	-2.90148115	1.560816171	-4.476680756	Q6AY18
GTPase related	Sbf1	SET binding factor 1; SET binding factor	23.03126	22.26654	27.72058	27.641	28.47448	28.3277	28.71614	29.14014	28.68509	28.58799	28.36924	29.25957	+	+	+	1.260231065	3.499773026	1.283210475	3.560628414	D3ZNN0;M0RAP5
GTPase related	Syngap1	synaptic Ras GTPase activating protein 1	21.72736	22.88175	24.87417	22.20345	29.63343	29.26152	29.96925	29.57935	29.87551	29.55967	29.91528	29.78466	+	+	+	4.098428187	6.689206123	4.198081792	6.862094402	2;F1LRF8;Q9QUH6-
GTPase related	Wdr7	WD repeat domain 7	21.67858	23.03637	22.40365	21.57164	29.44192	29.8399	29.67442	29.41288	29.71908	29.81359	29.60244	30.25856	+	+	+	6.094303217	7.419718266	6.081356824	7.675854206	Q9ERH3;Q9ERH3-2
Kinase	Ak1	adenylate kinase 1	22.84874	26.13481	21.39662	22.78384	29.95352	29.29527	29.8493	29.60989	29.85735	29.65285	30.19988	29.77032	+	+	+	3.122361199	6.385990143	3.199722755	6.57909584	P39069;M0RC66
Kinase	Camk2a	calcium/calmodulin-dependent protein	29.7228	30.87019	30.22458	29.30389	31.94045	32.69088	32.66793	32.7025	32.49279	32.45066	32.7207	32.67101	+	+	+	3.166058345	2.470073223	3.513993111	2.553424358	P11275;F1LZG4
Kinase	Camkv	CaM kinase-like vesicle-associated	22.59284	22.22697	21.57827	22.24859	30.55426	29.08091	30.34879	29.44095	30.40192	29.56373	29.94629	29.94963	+	+	+	5.8149717	7.694557667	6.921326995	7.803722858	Q63092;F1LR80
Kinase	Cdc42bpb	CDC42 binding protein kinase beta (DM	21.57618	25.17953	25.26949	23.06145	27.52664	26.79278	27.60187	27.09073	28.47518	27.61751	27.9457	28.06229	+	+	+	2.055690983	3.481341362	2.467548535	4.253506184	Q7TT49
Kinase	Hk1	hexokinase 1	26.31872	27.29097	28.19315	22.91	30.65514	30.07035	31.11867	30.57254	30.8838	30.38833	30.86127	31.05265	+	+	+	2.031277069	4.42596817	2.132472276	4.618305206	P05708
Kinase	Kalrn	kalirin, RhoGEF kinase	22.56763	23.2769	21.00076	22.39475	25.86261	26.11006	25.36189	25.62109	26.08438	25.91438	26.77518	26.03683	+	+	+	3.312590792	3.428904533	3.558250227	3.892681122	7;F1LZV1;D4ADV6;P97924-
Kinase	Mapk1	mitogen-activated protein kinase 1	24.82026	23.34075	25.4693	29.39989	30.17783	30.20326	30.21778	29.84661	30.07061	29.72038	30.0966	30.06702	+	+	+	1.816644782	4.353823185	1.765368577	4.231104851	P63086
Kinase	Mapk10	mitogen-activated protein kinase 10	21.79876	22.33946	22.18263	22.09526	25.90447	21.9611	26.20512	25.80681	25.76807	23.26746	25.15599	26.11989	+	+	+	1.523115128	2.865347385	2.435789921	2.973828316	D3ZQ33;P49187
Kinase	Mapk3	hypothetical LOC100271831; mitogen-a	22.73699	21.76182	21.9155	22.58524	26.88716	26.27279	26.2065	26.68485	26.89435	26.69657	26.30487	26.36878	+	+	+	5.19111245	4.262937069	5.339228248	4.316253662	P21708-2;P21708
Kinase	Mark2	MAP/microtubule affinity-regulating kin	21.86083	22.67513	26.57744	23.14516	27.19225	27.62275	26.59035	26.33141	29.06258	27.44569	26.69936	27.19065	+	+	+	1.6881151	3.369549274	1.88380649	4.034927368	O08679
Kinase	Mink1	misshapen-like kinase 1 (zebrafish)	23.20788	22.47443	22.8915	22.07904	28.13805	26.61583	27.65116	25.77216	28.31752	27.52806	27.6478	28.01813	+	+	+	3.537492381	4.381086826	5.594138708	5.214665413	F1LN69;F1LP90
Kinase	Mtor	mechanistic target of rapamycin (serine	23.72906	23.66105	22.8094	23.15027	26.55723	29.00019	27.84625	26.73982	28.6006	28.39477	28.25917	27.77354	+	+	+	3.349636541	4.198427677	5.658649986	4.9195714	P42346
Kinase	Pdxk	pyridoxal (pyridoxine, vitamin B6) kinas	21.86801	21.81405	21.39144	22.95189	28.14977	27.97596	27.2964	27.77197	27.84224	28.54447	28.39607	28.39423	+	+	+	5.295796786	5.792180061	5.597364526	6.287909508	G3V647;O35331;D3Z8P5
Kinase	Pgk1	phosphoglycerate kinase 1	22.56118	22.69036	28.00512	28.20765	32.03597	31.55742	31.87763	31.19531	31.52515	31.55403	31.73125	30.96836	+	+	+	2.124370459	6.300503254	2.057205444	6.07861948	P16617
Kinase	Pkm2	phospholipase A2-activating protein	21.34383	21.58172	26.38716	22.50062	30.81	30.47373	31.6516	31.33862	30.40598	30.60797	31.67766	31.71444	+	+	+	3.290691929	8.115157604	3.260252344	8.148183346	P11980
Kinase	Prkaca	protein kinase, cAMP-dependent, cataly	24.06876	23.15263	22.10938	21.48601	28.6656	27.24236	28.32367	28.05279	28.30526	23.57019	28.4556	28.50233	+	+	+	3.783384685	5.366909981	1.817041745	4.504149437	27791-2
Kinase	Prkacb	protein kinase, cAMP-dependent, cataly	24.20334	21.64278	27.11048	27.67283	29.99456	29.55591	29.98019	30.10649	29.61966	30.11508	29.93199	29.84407	+	+	+	1.833327095	4.751928329	1.823128311	4.720340729	P68182
Kinase	Prkar2a	protein kinase, cAMP-dependent, regul	22.95045	21.36192	21.40843	23.28247	26.19423	26.19265	26.54585	27.06199	26.10943	25.99736	25.17718	26.19449	+	+	+	3.630485157	4.247860909	3.202035129	3.618795872	G3V8Q6;P12368
Kinase	Prkar2b	protein kinase, cAMP-dependent, regul	23.53733	24.73092	25.64587	22.10913	29.04437	29.00681	29.61903	29.51786	29.05056	28.94999	29.68135	29.35342	+	+	+	3.294251533	5.291206837	3.2732178	5.253020287	P12369
Kinase	Prkca	protein kinase C, alpha	24.07649	23.10899	22.16449	23.45102	28.39252	28.59808	28.41852	28.86756	29.04218	28.95124	28.68996	28.66821	+	+	+	4.888714238	5.368919373	5.036291562	5.637648582	F1LS98;P05696;F1M2P8
Kinase	Prkcb	protein kinase C, beta	22.15062	22.52439	21.9781	21.46427	31.26651	30.79289	30.78667	30.98223	31.34064	30.92578	30.9458	31.15394	+	+	+	7.52065555	8.92773056	7.62791654	9.062194824	2;P68403;F1LS42;F1LS36
Kinase	Prkcd	protein kinase C, delta	22.44494	23.19565	22.86227	21.74418	27.20909	27.22057	26.58978	26.79835	27.62568	27.28851	26.47681	27.19018	+	+	+	4.807702316	4.392690182	4.606659602	4.583536148	P09215
Kinase	Prkce	protein kinase C, epsilon	23.50547	22.77849	23.60107	22.34605	27.16835	26.96025	27.7413	27.64842	27.46544	26.16062	27.79817	28.05745	+	+	+	4.735734321	4.321811199	3.792488852	4.31264925	P09216
Kinase	Prkcg	protein kinase C, gamma	23.65537	28.22651	23.36043	24.24637	31.54536	31.5949	31.82794	31.80147	31.54494	31.82115	31.78004	32.04808	+	+	+	3.018232988	6.820249081	3.048625175	6.926381588	P63319
Kinase	Tnik	TRAF2 and NCK interacting kinase	21.79238	21.33655	23.20203	21.2416	27.06363	23.18375	25.79749	25.5195	26.79017	25.82905	25.83716	26.4311	+	+	+	2.034051427	3.4979496	3.832590881	4.328727245	D3ZZQ0
Kinase related	Akap12	A kinase (PRKA) anchor protein 12	23.04364	22.19138	22.72081	24.04542	26.5423	25.71782	25.99187	25.47095	26.74111	26.03412	26.69325	27.00485	+	+	+	3.189359071	2.930425644	3.750151427	3.618025303	2;Q5QD51-3;E9PU09
Kinase related	Akap5	A kinase (PRKA) anchor protein 5	22.74132	22.15919	22.78479	23.78912	27.41877	27.63872	27.48031	26.84458	27.245	27.69604	28.2695	27.76939	+	+	+	4.645103854	4.476989746	4.743958003	4.876376629	F1LPP6;P24587
Kinase related	Akap7	A kinase (PRKA) anchor protein 7	21.92544	22.49915	22.72627	21.48588	26.21466	25.39375	26.20553	25.88157	26.00146	25.60043	26.20957	26.38693	+	+	+	4.486526061	3.764693737	4.665416417	3.890413761	Q6JP77;Q6JP77-2
Kinase related	Akap9	A kinase (PRKA) anchor protein (yotiao)	22.62881	23.02971	22.49994	22.00935	30.28008	29.10632	30.25991	30.13895	23.85158	26.64375	24.74031	24.3887	+	+	+	6.126787089	7.40435791	1.984919739	2.364131451	F1LPB4
Kinase related	Camk2b	calcium/calmodulin-dependent protein	22.70345	22.61308	27.29684	21.06069	29.58368	30.11284	29.83204	29.8044	29.71249	29.93227	29.80604	29.95366	+	+	+	2.499669491	6.414726257	2.510591	6.432600498	G3V9G3;P08413
Kinase related	Cnksr2	connector enhancer of kinase suppress	22.64712	22.45493	20.71127	22.46264	24.76132	25.54834	25.73774	25.66139	26.87479	25.65731	24.16354	22.33361	+	+	+	3.241270418	3.358207226	1.325649454	2.688323021	Q9Z1T4;Q9Z1T4-2
Kinase related	Csnk2b	lymphocyte antigen 6 complex, locus G	21.19475	24.17277	21.69821	21.24516	25.6279	26.2611	26.90644	26.49703	26.39728	22.40484	27.75085	27.34545	+	+	+	2.865209705	4.245392323	1.482081946	3.896882057	P67874
Kinase related	Gak	cyclin G associated kinase	22.38228	21.8271	23.61842	21.14307	25.23623	24.11598	25.5559	25.30247	27.05434	26.2151	25.27249	25.77515	+	+	+	2.422708984	2.809922695	2.99266984	3.836550236	F1LMD9;P97874
Kinase related	Pea15	phosphoprotein enriched in astrocytes	22.227	21.90868	22.88887	22.38926	28.63333	28.20313	28.52958	29.19345	28.31056	28.85308	29.12576	29.94196	+	+	+	6.19866783	6.286417961	5.561810839	6.704385281	Q5U318
Lysosome/Peroxisome	Acaa1a	acetyl-Coenzyme A acyltransferase 1A	22.46078	21.97722	22.17833	23.53017	27.18556	24.87098	26.95693	26.79551	26.77178	25.98548	27.62471	27.23233	+	+	+	3.076741812	3.915620804	3.93626652	4.366951466	B84;P21775-2
Lysosome/Peroxisome	Acot8	acyl-CoA thioesterase 8	22.40746	22.13415	22.82048	22.78896	25.44047	25.88061	25.21738	25.17417	25.04627	25.77138	25.4637	25.35222	+	+	+	4.804800878	2.890398502	4.879348738	2.870631218	F7F557;Q8VHK0
Lysosome/Peroxisome	Acsl3	acyl-CoA synthetase long-chain family m	22.98687	23.8359	22.32987	23.19434	26.72936	27.38747	26.63339	26.89435	27.54075	27.33356	27.81794	27.46614	+	+	+	4.438222422	3.824399948	5.013714288	4.452852726	Q63151;Q63151-2
Lysosome/Peroxisome	Acsl6	acyl-CoA synthetase long-chain family m	23.02431	21.90789	22.96309	23.28184	28.32672	28.5995	28.90759	27.82566	28.70154	28.53712	29.20469	28.89218	+	+	+	5.218737202	5.620584011	5.722965128	6.039600849	P33124-2;P33124
Lysosome/Peroxisome	Ctsd	cathepsin D	23.09881	26.83581	23.28148	22.14772	27.3554	27.54038	26.39883	27.17188	27.19592	27.00603	27.26553	26.82505	+	+	+	1.671777773	3.275666237	1.690501271	3.232175827	P24268;Q6P6T6
Lysosome/Peroxisome	Isoc1	isochorismatase domain containing 1	22.71152	21.73975	23.10635	21.73113	25.14161	26.19387	25.87359	26.33753	25.71382	26.17348	26.31043	26.29423	+	+	+	3.729718515	3.564462662	4.269062054	3.800804615	F2Z3T7;Q6I7R3
Lysosome/Peroxisome	Ppt1	palmitoyl-protein thioesterase 1	21.72529	21.57656	23.50991	21.2935	25.38972	26.98185	26.31055	26.48388	25.09893	27.38952	25.68225	26.43941	+	+	+	3.399125696	4.26518631	2.957283376	4.126213551	P45479
Melanosome	Ahcy	adenosylhomocysteinase	21.65353	21.68059	21.17676	21.66699	26.9927	25.74494	26.26288	25.99034	27.26535	26.13438	26.74701	24.94784	+	+	+	5.39986848	4.703248501	4.033703507	4.729180813	P10760
Melanosome	Ganab	glucosidase, alpha; neutral AB	22.69747	22.32132	24.50333	22.40392	29.31073	28.32586	29.46094	29.15228	29.22359	28.57636	28.86482	29.27819	+	+	+	4.38568785	6.080943584	4.504286825	6.004230499	D3ZAN3
Melanosome	Sypl1	synaptophysin-like 1	21.70077	21.81445	21.8324	22.71671	25.20165	25.53825	25.90254	25.69107	25.74755	25.3644	25.91194	25.90258	+	+	+	4.863320539	3.567296028	5.057571944	3.715537071	Q66H18
Membrane protein	Efr3b	EFR3 homolog B (S. cerevisiae)	21.6764	21.92039	22.81398	22.18801	27.37509	22.85648	27.58685	27.51239	27.15054	25.00552	27.25467	27.77304	+	+	+	1.907478832	4.183009148	3.389085794	4.646247864	F1LTW9;F1M5F9
Membrane protein	Tmem189	ubiquitin-conjugating enzyme E2 varian	23.97528	22.65416	22.57456	22.29317	29.01055	27.25665	29.41062	28.36215	28.78404	27.53853	29.20893	29.30051	+	+	+	4.076127907	5.635694981	4.37198333	5.833704948	D3ZFY8;F1M227
Metabolic	Acyp1	acylphosphatase 1, erythrocyte (comm	22.46893	21.77702	22.11994	21.95011	26.31843	26.24833	26.74227	26.3394	27.03946	26.86077	27.19272	27.3657	+	+	+	6.398603943	4.333106041	6.820912202	5.035661697	D4A6X4
Metabolic	Adh5	alcohol dehydrogenase 5 (class III), chi p	21.09304	21.54867	23.36368	22.94467	26.64058	26.71712	27.72981	27.68552	27.44411	27.35607	27.85608	27.82893	+	+	+	3.688860467	4.955744267	4.143320843	5.383784771	P12711
Metabolic	Agl	amylo-1, 6-glucosidase, 4-alpha-glucan	20.12546	21.83526	22.17439	23.6593	27.00174	23.15874	26.91285	26.50343	26.80144	22.4266	27.45772	27.26544	+	+	+	1.823609104	3.945587158	1.55725792	4.039194107	D4AEH9
Metabolic	Akr1a1	aldo-keto reductase family 1, member A	21.27307	21.46252	23.66909	23.01259	28.12741	28.30861	28.45998	28.58075	27.9975	28.62833	28.78104	29.013	+	+	+	4.266032556	6.014870644	4.235869667	6.250647545	P51635
Metabolic	Akr1b1	aldo-keto reductase family 1, member B	22.78024	22.65795	22.37493	21.36876	28.63433	28.27004	26.83894	27.87467	27.98701	28.12805	28.38796	28.35834	+	+	+	4.509202487	5.609026432	5.680958832	5.919872761	P07943

Supplementary Table 2. List of proteins significantly enriched in both immunoisolated FEZ1 and Kinesin-1 containing vesicles by quantitative mass spectrometry

Group	Gene Symbol	Gene Name	log LFQ intensity IP1 Control	log LFQ intensity IP2 Control	log LFQ intensity IP3 Control	log LFQ intensity IP4 Control	log LFQ intensity IP1 Kinesin-1	log LFQ intensity IP2 Kinesin-1	log LFQ intensity IP3 Kinesin-1	log LFQ intensity IP4 Kinesin-1	log LFQ intensity IP1 FEZ1	log LFQ intensity IP2 FEZ1	log LFQ intensity IP3 FEZ1	log LFQ intensity IP4 FEZ1	t-test Significant Kinesin-1 IP	t-test Significant FEZ1 IP	t-test Significant Kinesin-1 and FEZ1 IP	-Log t-test p value Kinesin-1 IP	t-test Difference Kinesin-1 IP	-Log t-test p value FEZ1 IP	t-test Difference FEZ1 IP	Majority UniProt protein IDs
Metabolic	Aldoa	aldolase A, fructose-bisphosphate	22.58997	27.43094	29.88056	29.44804	33.15872	32.01754	32.96141	33.59034	33.11514	32.48008	33.1411	33.05879	+	+	+	1.777193988	5.594625473	1.809355272	5.611400604	P05065;Q6AY07
Metabolic	Alg2	asparagine-linked glycosylation 2, alpha	23.08611	23.3472	24.79531	23.25913	28.07602	28.82387	28.53249	28.67	28.41022	28.69445	27.58534	28.18641	+	+	+	4.584833285	4.90365696	4.237101689	4.597167969	G3V6U3
Metabolic	Asrgl1	asparaginase like 1	23.139	22.50794	23.48348	23.0288	28.28551	27.81592	28.23567	27.90035	28.90458	28.21521	28.66017	28.41333	+	+	+	6.175361035	5.019559383	6.229081127	5.508518696	Q8VI04
Metabolic	Blvra	biliverdin reductase A	20.9806	22.86722	21.71645	21.58498	26.64897	26.21054	26.2331	25.96215	26.89897	26.02644	26.65075	26.48431	+	+	+	4.401803437	4.476376534	4.445478177	4.727804661	Q6AZ33
Metabolic	Bpnt1	3'(2'), 5'-bisphosphate nucleotidase 1	23.09325	22.508	21.89635	22.95363	26.32322	25.53677	26.4261	25.63101	26.62965	25.55025	26.34499	26.23834	+	+	+	4.110926442	3.366462231	4.266573156	3.577997684	Q9Z1N4
Metabolic	Ca2	carbonic anhydrase II	22.88697	23.20621	21.87079	22.69801	28.49167	29.17757	29.21517	28.94061	28.40145	29.18931	29.41897	29.00126	+	+	+	5.871150909	6.290761471	5.675711891	6.337253571	P27139
Metabolic	Cbr1	carbonyl reductase 1	22.89741	21.46688	22.63153	20.81132	27.91633	27.36828	28.11098	26.64169	28.16433	27.50036	28.34908	26.56995	+	+	+	4.085063797	5.557534695	3.969888653	5.694144726	P47727;M0R3X6
Metabolic	Ddost	dolichyl-diphosphooligosaccharide-prot	23.22021	25.33446	21.65445	21.93792	27.52253	28.21688	27.67654	28.14851	27.70068	27.82748	27.2238	27.18679	+	+	+	2.889008408	4.854353428	2.69921112	4.447925568	Q641Y0;F1LM69
Metabolic	Ddt	D-dopachrome tautomerase	23.05851	22.60138	22.36494	22.25637	30.30672	30.02934	31.08144	30.63694	30.97076	30.3349	30.94266	31.08303	+	+	+	6.823732045	7.943309784	7.341944276	8.262537003	P80254
Metabolic	Eno1	enolase 1, (alpha)	21.93513	26.4441	29.76992	22.8452	31.07546	31.22314	32.38564	32.51841	31.16884	31.19384	33.17485	32.50829	+	+	+	1.930478056	6.552075863	1.961017899	6.762866974	M0R5J4;P04764;M0RAU4
Metabolic	Eno2	enolase 2 (gamma, neuronal)	22.56777	25.22758	28.33074	27.8749	31.37322	30.29483	31.4815	31.98237	31.39202	31.51719	32.60587	31.77576	+	+	+	2.062188939	5.282728195	2.283025614	5.822463512	P07323
Metabolic	Enoph1	enolase-phosphatase 1	21.97787	21.49121	22.39347	24.35375	26.63727	26.8867	27.00753	26.89041	26.03244	26.33994	27.26033	26.40001	+	+	+	3.305620491	4.301400661	2.942590929	3.954104424	Q5PPH0
Metabolic	Enpp6	ectonucleotide pyrophosphatase/phosp	21.4872	22.79959	21.68321	22.6113	26.18535	24.38456	25.39257	24.96601	25.91306	22.97735	24.90665	24.65157	+	+	+	3.075013305	3.086797714	1.926176865	2.466831684	B0BND0;D3ZRI1
Metabolic	Fabp5	fatty acid binding protein 5-like 2; fatty	22.94205	23.7946	22.32717	23.16564	29.76822	29.85604	29.90426	30.13712	30.06227	29.60489	30.49457	30.5681	+	+	+	6.228559898	6.859044552	5.855684692	7.125094891	P55053
Metabolic	Fabp7	fatty acid binding protein 7, brain	23.88443	20.67345	22.09352	22.19299	30.18931	29.45262	29.38268	29.04515	29.5675	29.60525	29.57557	29.20923	+	+	+	4.344441772	7.306341171	4.467044628	7.278288841	P55051
Metabolic	Fam213a	family with sequence similarity 213, me	22.63559	23.24722	22.12614	21.96431	28.27866	28.31047	28.05041	28.32311	28.44668	28.00201	27.63062	28.3256	+	+	+	5.916289934	5.747344494	5.483874055	5.607910633	Q6AXX6
Metabolic	Fam213b	family with sequence similarity 213, me	22.37183	23.47705	23.18563	22.65211	26.13694	25.40873	25.26774	25.30574	26.7929	25.75921	24.7225	25.38926	+	+	+	3.710737803	2.608132839	2.815576377	2.744312763	D3ZVR7
Metabolic	Folh1	folate hydrolase (prostate-specific mem	22.82527	23.36748	22.69309	22.10832	27.72761	27.45951	27.40738	27.48408	27.16595	27.76281	27.69757	26.73363	+	+	+	5.693827214	4.771107197	4.886044574	4.591451645	G3V7S0;P70627
Metabolic	Gapdh	glyceraldehyde-3-phosphate dehydroge	36.89227	38.36096	38.10505	37.98354	34.33538	35.84581	35.15021	35.71339	34.46508	35.58153	34.3628	34.33191	+	+	+	2.799816417	-2.57425785	3.417312945	-3.15012455	0R660
Metabolic	Gda	guanine deaminase	22.48787	21.96992	22.02844	23.52561	29.16935	28.56674	29.78362	29.57634	29.57863	28.66607	30.02009	29.58312	+	+	+	5.271692149	6.771053791	5.284292645	6.959020615	Q9JKB7;Q9WTT6
Metabolic	Gde1	glycerophosphodiester phosphodiester	21.37992	21.97508	21.72858	22.50907	25.46438	22.91937	25.06837	25.26882	25.19456	24.71225	22.62751	25.06044	+	+	+	2.320774388	2.78207159	2.087905507	2.500524521	Q9JL55;M0R5Q9
Metabolic	Gdpd1	glycerophosphodiester phosphodiester	22.96782	23.71316	22.67142	21.97187	24.98425	26.6539	25.90557	26.08227	25.15186	26.47122	25.90985	26.13751	+	+	+	3.072233304	3.075428486	3.291198461	3.086544037	Q0VGK4
Metabolic	Ggt7	gamma-glutamyltransferase 7	22.37354	21.82538	22.06059	23.22626	26.93134	26.34147	26.50817	26.46543	27.01999	26.22521	26.15368	26.49645	+	+	+	4.81931106	4.190158844	4.535991902	4.102389336	Q99MZ4
Metabolic	Glo1	glyoxalase I	21.41687	21.05062	22.64245	23.5729	28.86547	28.05103	29.00102	28.99286	28.48128	27.92751	28.85703	29.01236	+	+	+	4.370589744	6.556881428	4.289502248	6.398832321	Q6P7Q4
Metabolic	Glrx	glutaredoxin (thioltransferase)	23.38111	20.44988	22.62177	23.91101	27.35691	26.78144	27.32208	27.35237	27.64705	27.77411	27.4649	28.87883	+	+	+	2.999900736	4.612257957	3.193807925	5.350277424	Q9ESH6;Q6AY00
Metabolic	Glul	glutamate-ammonia ligase (glutamine s	26.58735	28.47866	29.72455	28.66458	31.57123	31.37653	31.88234	32.28997	31.94489	31.52981	31.80336	32.15864	+	+	+	2.61310473	3.416233063	2.716656405	3.495388985	P09606
Metabolic	Got1	glutamic-oxaloacetic transaminase 1, so	23.85777	21.68298	26.21353	28.1775	31.14698	31.28328	31.89883	32.16075	31.43991	31.49629	31.70607	31.80139	+	+	+	2.450430432	6.63951683	2.473685796	6.627972126	P13221
Metabolic	Gsta3	glutathione S-transferase alpha 3	22.04442	22.08053	23.0517	22.75585	28.11297	27.14754	26.79786	26.8839	28.14433	27.94324	27.35279	26.77442	+	+	+	4.717385925	4.752443314	4.843418477	5.070573807	P04904;M0RDI1
Metabolic	Gstm1	glutathione S-transferase mu 1	22.18081	22.14016	21.72449	22.94663	28.90656	28.38327	28.52249	28.48504	28.32294	29.08208	28.32122	28.60604	+	+	+	6.314823246	6.326321602	6.037241424	6.335050106	P04905;G3V983
Metabolic	Gstm3	glutathione S-transferase mu 3 (brain)	22.65591	21.44028	22.52356	21.27981	30.18127	30.31203	29.5987	29.76226	29.85268	30.37493	30.18837	30.5037	+	+	+	6.019852849	7.988675117	6.176584227	8.255029202	P08009
Metabolic	Gstm5	glutathione S-transferase mu 5	22.33088	21.89805	22.7784	23.63822	27.72592	26.97005	27.7377	27.70154	27.81855	27.64904	28.13124	27.91752	+	+	+	4.626850497	4.872414112	4.997495166	5.217700481	Q9Z1B2
Metabolic	Gsto1	glutathione S-transferase omega 1	22.23863	23.43934	22.86747	21.99582	28.84909	29.35256	29.21153	28.97902	28.59096	29.07982	28.5204	28.97136	+	+	+	5.832672734	6.462735653	5.641366767	6.155320168	Q6AXR6;Q9Z339
Metabolic	Gstp1	glutathione S-transferase pi 1	22.21678	22.41017	22.66046	22.31188	28.44845	28.76907	28.71221	28.70474	28.33807	28.87581	29.39667	28.50892	+	+	+	8.493385595	6.258797169	6.592620882	6.380048752	P04906
Metabolic	Hint1	histidine triad nucleotide binding protei	23.21793	23.38989	21.95626	21.93883	30.26204	30.80908	30.7005	30.98468	30.13712	31.04876	31.12201	31.14862	+	+	+	5.875334935	8.063348293	5.697509537	8.238399982	P62959;D4A269
Metabolic	Hmox2	heme oxygenase (decycling) 2	23.23449	23.47716	23.22259	22.60544	27.77228	28.12593	27.45725	27.14802	27.24291	28.19784	27.17741	26.87631	+	+	+	5.424616845	4.490951061	4.779259241	4.238696098	P23711
Metabolic	Hprt1	hypoxanthine phosphoribosyltransferas	21.85728	22.22239	25.16615	21.54262	27.92626	27.54665	28.15403	27.91108	27.84571	27.7348	28.12199	27.6725	+	+	+	3.070586963	5.187393665	3.062018712	5.14663887	P27605;F1LNY0
Metabolic	Lnpep	leucyl/cystinyl aminopeptidase	21.77288	21.81944	22.57137	21.13421	25.42079	21.9517	26.06447	26.03951	21.34131	26.54069	25.74901	25.0964	+	+	+	1.600117067	3.044645309	1.275300716	2.857379913	P97629;D3ZUR9
Metabolic	Lypla2	lysophospholipase II pseudogene 1; lysc	22.87599	23.18842	22.98811	22.14203	25.24096	26.27254	25.60204	26.14684	22.98048	26.11406	25.47268	26.69617	+	+	+	4.004484987	3.016957283	1.600293419	2.51721096	Q9QYL8
Metabolic	Mblac2	metallo-beta-lactamase domain contain	22.41784	23.35261	23.837	22.05485	26.78718	26.92128	27.05331	26.49222	26.73738	26.91011	27.28806	26.97082	+	+	+	4.00481252	3.897925854	4.113583587	4.061019897	D4A249
Metabolic	Mpst	mercaptopyruvate sulfurtransferase	23.3533	23.04589	23.47154	22.78456	25.43517	25.91495	26.17845	26.01595	25.59807	26.06365	25.99302	26.31667	+	+	+	4.739541426	2.722309589	4.926193378	2.829032421	P97532
Metabolic	Mri1	methylthioribose-1-phosphate isomeras	22.80918	22.11557	21.75031	22.74218	25.15422	25.23528	25.63542	24.81705	24.71015	24.99235	25.34069	26.35963	+	+	+	4.072920182	2.856181145	3.302095772	2.996395111	Q5HZE4
Metabolic	Nit2	nitrilase family, member 2	21.64808	22.74853	23.16247	23.07783	25.89104	26.46709	26.60696	26.31349	25.956	26.35429	26.95914	26.67761	+	+	+	4.134662356	3.660420418	4.067165217	3.827536106	Q497B0
Metabolic	Ola1	Obg-like ATPase 1	22.21658	22.41297	22.77955	23.82534	27.45144	26.10115	26.75187	26.38227	27.0624	26.13853	26.78481	26.60201	+	+	+	3.796499544	3.863073349	4.088784772	3.838326454	A0JPJ7
Metabolic	Pafah1b2	platelet-activating factor acetylhydrolas	22.20236	23.19911	22.00908	23.00383	27.91393	27.75882	28.22366	28.15427	28.31609	28.06948	27.91416	28.21489	+	+	+	5.627152943	5.409074306	5.733792467	5.525062084	O35264
Metabolic	Pfkl	phosphofructokinase, liver	23.86567	23.1819	23.04933	22.30295	28.55907	28.66003	28.52376	29.11399	28.24735	28.21304	28.75309	28.71728	+	+	+	5.442378073	5.614251137	5.306162452	5.3827281	P30835
Metabolic	Pgam1	phosphoglycerate mutase 1 (brain)	22.76635	22.94282	29.40919	28.71594	31.94987	31.96695	31.90591	31.98814	31.94423	31.96582	32.10509	32.0583	+	+	+	1.803086488	5.994144917	1.822404716	6.059785843	P25113
Metabolic	Pgls	6-phosphogluconolactonase	21.15207	21.30292	21.60238	22.02572	26.41727	27.52335	26.24688	26.43598	26.22372	27.50302	26.38346	26.41798	+	+	+	5.200837041	5.13509798	5.182226039	5.111269951	G3V8D5;P85971
Metabolic	Ppa1	pyrophosphatase (inorganic) 1	22.24473	22.13517	22.12997	21.85898	27.72449	27.75704	28.23977	27.68786	27.5136	28.05289	28.4545	27.75583	+	+	+	7.617931627	5.760075569	6.744708391	5.851992607	F7EPH4
Metabolic	Prep	prolyl endopeptidase	21.93295	22.9623	22.70121	23.31858	26.09324	26.00374	26.28694	25.99496	26.87079	26.4379	27.23927	26.72182	+	+	+	4.510283474	3.365958691	4.71294744	4.088686943	O70196
Metabolic	Prps1	phosphoribosyl pyrophosphate syntheta	22.43853	23.97533	26.68472	21.79854	27.71947	27.14686	27.13222	27.68438	28.05248	26.98174	27.57687	27.49876	+	+	+	1.818412371	3.696453094	1.854003936	3.803183079	P60892;M0RBK1
Metabolic	Psat1	chromosome 8 open reading frame 62;	23.08042	22.62658	23.3785	21.78083	29.04939	29.12989	29.28505	28.90165	29.42992	28.71787	29.34511	29.31452	+	+	+	5.70145074	6.374911785	5.556961615	6.485272408	E9PSV5
Metabolic	Ptges3	prostaglandin E synthase 3 (cytosolic)	21.43517	22.81382	22.43868	21.68403	27.54973	27.43627	28.24418	28.38364	27.51156	27.52962	28.17012	28.88381	+	+	+	5.169579104	5.810528755	4.889567645	5.930850983	P83868
Metabolic	Pygb	phosphorylase, glycogen; brain	22.93358	27.15962	30.17878	29.49928	31.35232	31.24955	31.64642	31.61632	31.09723	31.29324	31.5858	31.59308	+	+	+	1.305493816	4.02333498	1.277587917	3.949522495	G3V6Y6;P53534
Metabolic	Pygm	phosphorylase, glycogen, muscle	22.72518	21.20079	21.80501	22.38852	28.81684	28.77216	29.16646	28.91932	29.00924	29.13604	29.2994	29.28337	+	+	+	5.976277218	6.888817787	6.106418971	7.152137756	G3V8V3;P09812
Metabolic	Sec14l2	SEC14-like 2 (S. cerevisiae)	21.96214	20.83472	22.77179	21.69827	26.51488	26.87888	26.63215	25.98635	26.8715	27.26347	26.79514	25.89479	+	+	+	4.386567723	4.686336517	4.217073764	4.88949585	Q5EBD0;Q99MS0
Metabolic	Taldo1	transaldolase 1	22.15469	22.14746	23.40421	21.52336	26.80894	24.38541	25.90261	26.01314	26.79228	22.53653	24.87413	25.95336	+	+	+	2.782407323	3.470095158	1.462773696	2.731645584	Q9EQS0
Metabolic	Thop1	thimet oligopeptidase 1	22.72215	23.02154	22.81265	21.25801	26.90518	24.98229	26.54339	26.80833	26.8597	25.58595	26.09475	26.98945	+	+	+	3.159556144	3.856209755	3.546425604	3.928874016	P24155
Metabolic	Tkt	transketolase	22.68295	22.79809	22.71169	23.13247	26.43184	23.4655	28.51096	25.49094	27.46544	22.16004	28.39787	28.07847	+	+	+	1.611756591	3.143506527	1.338357544	3.694151878	P50137
Metabolic	Tpi1	TPI1 pseudogene; triosephosphate isom	26.08582	27.73628	30.07368	29.87438	32.41455	32.34276	32.90851	33.14614	32.8073	32.7024	33.13202	33.07399	+	+	+	2.343511356	4.260454655	2.482121782	4.486390591	P48500
Metabolic	Txn	thioredoxin	29.01425	20.88909	23.01582	22.3174	29.54657	28.14501	29.88124	29.08934	29.98672	28.93509	29.52826	29.60246	+	+	+	1.578717453	5.35640049	1.709806043	5.703988552	P11232
Metabolic	Txnl1	thioredoxin-like 1	21.44553	22.66037	22.18406	22.96415	27.1419	27.29745	27.65963	27.50574	27.33074	27.62401	27.9962	28.00961	+	+	+	5.177755355	5.087651253	5.207592072	5.426612377	Q920J4

Supplementary Table 2. List of proteins significantly enriched in both immunoisolated FEZ1 and Kinesin-1 containing vesicles by quantitative mass spectrometry

Group	Gene Symbol	Gene Name	log LFQ intensity IP1 Control	log LFQ intensity IP2 Control	log LFQ intensity IP3 Control	log LFQ intensity IP4 Control	log LFQ intensity IP1 Kinesin-1	log LFQ intensity IP2 Kinesin-1	log LFQ intensity IP3 Kinesin-1	log LFQ intensity IP4 Kinesin-1	log LFQ intensity IP1 FEZ1	log LFQ intensity IP2 FEZ1	log LFQ intensity IP3 FEZ1	log LFQ intensity IP4 FEZ1	t-test Significant Kinesin-1 IP	t-test Significant FEZ1 IP	t-test Significant Kinesin-1 and FEZ1 IP	-Log t-test p value Kinesin-1 IP	t-test Difference Kinesin-1 IP	-Log t-test p value FEZ1 IP	t-test Difference FEZ1 IP	Majority UniProt protein IDs
Metabolic	Vkorc1l1	vitamin K epoxide reductase complex, s	21.92144	23.04597	22.64778	22.07575	24.66232	25.11728	25.41509	26.06093	25.57658	25.12353	25.23531	25.58809	+	+	+	3.497947482	2.891171932	4.323568558	2.958143711	Q6TEK3
Mitochondria	Acaca	acetyl-Coenzyme A carboxylase alpha	22.41162	22.07986	21.61032	21.34927	26.35392	26.60936	26.13835	25.52328	26.58506	25.47525	26.46109	26.87444	+	+	+	4.882403286	4.293457985	4.615082107	4.486191273	P11497;P11497-2
Mitochondria	Acat1	acetyl-Coenzyme A acetyltransferase 1	22.39181	21.87589	23.18747	22.74066	27.51623	28.02115	28.50544	27.69112	27.88262	28.53871	28.43066	28.15369	+	+	+	5.304648069	5.384530544	5.746872791	5.702462673	P17764
Mitochondria	Acat2	acetyl-Coenzyme A acetyltransferase 2	22.19671	21.89499	22.94093	22.34883	27.94386	21.65281	27.7934	26.72611	27.77643	26.46661	26.3821	26.56408	+	+	+	1.306358635	3.683679581	4.533597566	4.451941013	Q5XI22;F1LS48;D3ZKB6
Mitochondria	Acly	ATP citrate lyase	22.18823	22.22509	24.52073	23.53612	28.77944	29.08598	29.44829	29.06147	28.657	29.28518	29.57479	29.38146	+	+	+	4.313557947	5.976253033	4.293328608	6.107065678	6638-2
Mitochondria	Aco2	aconitase 2, mitochondrial	22.22708	21.16091	25.85963	22.53068	31.27974	30.99725	31.16015	30.91563	31.27353	31.37689	31.465	31.18636	+	+	+	3.69051209	8.143618107	3.76334066	8.380870819	Q9ER34
Mitochondria	Acot7	acyl-CoA thioesterase 7	22.27063	22.2582	26.19432	25.60414	29.00758	29.05478	29.32071	29.16856	28.63308	28.35334	29.4492	29.38043	+	+	+	2.512442755	5.056086063	2.371415218	4.872189045	Q64559;F8WG67;Q64559-1
Mitochondria	Acyp2	acylphosphatase 2, muscle type	20.33238	21.60008	22.4166	22.67457	26.52099	25.6845	27.61506	26.54231	27.11197	27.27533	27.69339	27.03748	+	+	+	3.484880232	4.834807396	4.261834822	5.523634911	D4A1G1;P35745
Mitochondria	Akr7a2	aldo-keto reductase family 7, member A	22.66726	21.89902	22.10625	22.76547	26.48765	26.50596	25.8663	26.53891	25.68656	25.78162	26.33455	26.30567	+	+	+	5.258048564	3.990203381	4.991968522	3.667599201	Q8CG45
Mitochondria	Aldh1l1	aldehyde dehydrogenase 1 family, mem	22.21662	22.78617	24.34619	22.7526	27.51096	26.73337	27.61828	27.49968	27.49618	26.80107	27.73654	27.40706	+	+	+	3.8629979	4.315175056	3.884229048	4.334813595	P28037;M0R8T2
Mitochondria	Aldoc	aldolase C, fructose-bisphosphate	22.52297	27.29999	27.29815	28.39562	31.82354	31.69008	32.12124	32.64059	32.09993	31.99044	32.29535	32.41064	+	+	+	2.285668582	5.689677715	2.353028246	5.819904327	P09117
Mitochondria	Atp5a1	ATP synthase, H+ transporting, mitocho	23.02828	22.98077	26.92999	21.1743	30.81406	30.79931	30.10375	29.99537	30.97384	30.97289	31.34292	30.7108	+	+	+	2.858323222	6.899786472	3.062279262	7.471776485	P15999;F1LP05
Mitochondria	Atp5b	ATP synthase, H+ transporting, mitocho	24.18677	25.24808	26.12723	24.9644	31.31921	31.40614	31.75235	30.68662	30.53807	31.14017	31.36317	30.95775	+	+	+	4.979723129	6.159460545	4.975827133	5.868170738	G3V6D3;P10719
Mitochondria	Atp5c1	ATP synthase, H+ transporting, mitocho	20.99448	22.70236	20.52835	21.94378	28.73693	28.69478	28.36215	28.67731	28.64516	28.74182	28.70193	28.89954	+	+	+	5.141627978	7.075547695	5.211078676	7.204871655	F7FFJ9;P35435;Q6QI09
Mitochondria	Atp5d	ATP synthase, H+ transporting, mitocho	22.27929	22.81575	21.85656	22.16674	28.13237	27.99026	27.73441	27.48945	28.32281	28.54134	28.04229	28.24481	+	+	+	6.316361448	5.55703783	6.741141857	6.008226871	G3V7Y3;P35434
Mitochondria	Atp5f1	ATP synthase, H+ transporting, mitocho	22.53117	21.9887	23.00982	22.24431	28.62596	27.99884	28.16289	27.84428	28.18778	28.07607	28.15494	28.29439	+	+	+	6.077923061	5.714491367	6.636343844	5.73479557	P19511
Mitochondria	Atp5h	ATP synthase, H+ transporting, mitocho	22.19027	22.38933	22.1759	22.44863	29.07444	28.23868	28.42838	28.26069	29.12448	28.46408	29.29145	28.61263	+	+	+	7.026616324	6.199514389	7.146531738	6.572125435	P31399;D3ZWT8
Mitochondria	Atp5i	ATP synthase, H+ transporting, mitocho	21.37791	22.55153	22.11745	22.10526	28.90047	29.62542	28.90354	29.4003	29.46486	29.72115	28.87991	29.59272	+	+	+	6.418856515	7.169392586	6.474348369	7.376621246	P29419
Mitochondria	Atp5j2	ATP synthase, H+ transporting, mitocho	22.7336	23.72678	22.29962	22.09541	28.02422	28.19643	28.47018	28.64928	28.08603	28.95762	28.14195	29.08332	+	+	+	5.164053093	5.62117672	4.905188931	5.853378773	D3ZAF6
Mitochondria	Atp5l	ATP synthase, H+ transporting, mitocho	24.5946	21.66844	23.80149	22.21824	27.83744	27.81201	26.79774	27.17883	27.16768	27.61133	26.8375	28.0167	+	+	+	3.005639104	4.335810661	3.002538176	4.337607861	Q6PDU7
Mitochondria	Atp5o	ATP synthase, H+ transporting, mitocho	22.13078	22.69608	23.58333	23.99952	29.85992	29.38852	30.04244	29.7608	29.71047	29.60604	30.42812	30.33873	+	+	+	5.253494603	6.660494804	5.195086394	6.918414116	Q06647
Mitochondria	Bcat1	branched chain aminotransferase 1, cyt	22.89563	22.87659	21.77079	24.42823	26.51023	25.72619	26.00729	25.13493	26.56818	25.74537	26.37187	26.24746	+	+	+	2.44381992	2.85185194	2.880001572	3.240412235	P54690
Mitochondria	Bdh1	3-hydroxybutyrate dehydrogenase, type	22.64574	21.79423	23.76135	23.07224	26.92422	26.96356	27.25007	27.4768	27.36077	26.76408	27.49251	27.58492	+	+	+	4.255914152	4.325951576	4.221457372	4.482137203	P29147
Mitochondria	C1qbp	complement component 1, q subcompo	23.94641	22.12121	21.74772	22.16541	26.25176	27.4556	27.61898	27.0874	26.46103	26.93382	27.32612	26.54088	+	+	+	3.677365852	4.608249187	3.73020814	4.320275784	O35796
Mitochondria	C21orf33	chromosome 21 open reading frame 33	21.73275	21.74874	21.38263	23.65655	26.02168	26.3179	26.9997	26.96135	26.47104	26.94492	27.46894	27.40251	+	+	+	3.631548035	4.444990158	3.906068614	4.941684723	P56571
Mitochondria	Chchd3	coiled-coil-helix-coiled-coil-helix domair	21.61172	23.46805	22.49912	22.45867	25.62851	25.04293	25.54451	24.93315	25.73376	25.38021	24.22876	25.36369	+	+	+	3.252184132	2.77788496	2.747166601	2.667215347	D3ZUX5
Mitochondria	Cisd1	CDGSH iron sulfur domain 1	23.21803	21.59528	21.72566	23.82471	28.90481	27.81415	28.55856	28.39497	29.24665	28.37894	28.76094	29.08015	+	+	+	4.17714638	5.827198982	4.415433919	6.27574873	B0K020
Mitochondria	Ckb	creatine kinase, brain	29.0252	30.06484	30.47586	31.04616	33.15108	32.9656	33.71405	34.20208	32.78474	33.37723	33.61182	33.71947	+	+	+	3.225489625	3.355192184	3.299168627	3.220302582	P07335
Mitochondria	Ckmt1b	creatine kinase, mitochondrial 1A; creat	23.14462	21.77001	21.2044	25.53789	29.55353	29.12189	29.76457	30.13186	29.12761	29.62343	29.83351	29.46863	+	+	+	3.309747474	6.728730202	3.291292611	6.599063873	Q5BJT9;P25809
Mitochondria	Clic4	chloride intracellular channel 4	21.93879	21.61328	22.09629	21.66528	27.39999	26.85531	26.6133	26.70978	27.2469	27.14802	26.69922	26.30848	+	+	+	6.482324801	5.066185474	6.059298699	5.022248268	G3V8C4;Q9Z0W7
Mitochondria	Comtd1	catechol-O-methyltransferase domain c	22.87688	21.73427	23.17722	21.76822	24.65163	25.64058	25.32231	24.40639	24.68555	24.91278	25.20613	25.42666	+	+	+	2.841904332	2.616079807	3.218153738	2.668631077	D3ZM21
Mitochondria	Cox1	Cytochrome c oxidase subunit 1	21.75288	26.17807	25.4035	21.8006	29.74355	29.87185	29.70334	28.89024	28.8052	30.38113	29.89701	29.19962	+	+	+	2.542506442	5.768480778	2.495180792	5.786973476	Q8SEZ5;P00406
Mitochondria	Cox2	Cytochrome c oxidase subunit 2	21.55992	22.39965	22.06844	22.28309	26.26639	25.68373	26.73143	26.73879	25.63719	22.04339	26.91969	25.51412	+	+	+	5.035869678	4.277312279	1.495609761	2.950821877	P05503;Q8HIC9;Q8SEZ6
Mitochondria	Cox4i1	cytochrome c oxidase subunit IV isoforn	23.09785	21.18767	23.17298	21.02906	30.75334	29.98875	29.91385	30.26528	30.88569	30.23945	30.56329	30.41669	+	+	+	4.924505705	8.108415604	5.074645478	8.404392242	P10888
Mitochondria	Cox5a	cytochrome c oxidase subunit Va	22.90379	23.26104	22.08591	22.82997	29.96389	29.31521	29.25809	28.95244	29.731	29.36208	29.22766	29.42866	+	+	+	6.028263157	6.602231503	6.549591585	6.667175293	P11240
Mitochondria	Cox5b	cytochrome c oxidase subunit Vb	22.48076	21.96144	23.09564	21.53425	29.4931	28.84209	30.00316	28.8121	29.68757	28.65598	29.03855	28.66363	+	+	+	5.404429377	7.019590855	5.460610271	6.743409634	P12075
Mitochondria	Cox6b1	cytochrome c oxidase subunit Vib polyp	23.43459	22.39156	21.59813	24.08862	27.93438	27.21401	28.26428	28.21859	28.95798	27.31596	28.65116	28.92201	+	+	+	3.79448472	5.029587269	3.774621943	5.583553314	D3ZD09
Mitochondria	Cs	citrate synthase	22.129	22.99108	23.27679	24.10174	29.565	28.40495	29.17217	29.08116	29.61427	29.79922	29.65777	28.57409	+	+	+	4.803391851	5.931167126	4.835225289	6.286682129	G3V936;Q8VHF5
Mitochondria	Cyb5a	cytochrome b5 type A (microsomal)	21.61691	21.4966	22.48031	22.62836	27.93697	25.98076	26.63326	22.02804	27.93005	26.71057	26.85555	27.16327	+	+	+	1.477790964	3.589211941	4.862311696	5.109312534	P00173;D4A1G4;P00173-2
Mitochondria	Cyb5r3	cytochrome b5 reductase 3	23.53237	22.18625	22.82837	22.33345	27.43841	27.59641	27.2787	27.22509	27.04469	27.22922	27.51458	27.67169	+	+	+	5.224788966	4.664540768	5.060413497	4.64493084	P20070-2;P20070;P20070-3
Mitochondria	Cycs	cytochrome c, somatic	23.34307	23.49391	24.24808	21.63407	29.24361	28.20041	28.98381	28.31946	29.47933	28.9755	29.19018	29.19098	+	+	+	3.996194301	5.507041931	4.413799108	6.029216766	D4A5L9;P62898;M0RBT2
Mitochondria	Ddah1	dimethylarginine dimethylaminohydrola	22.3276	23.55194	22.83866	21.93499	28.78852	28.06769	28.28916	27.52036	28.43869	27.97049	27.9969	28.07362	+	+	+	4.813192018	5.503138065	5.243865494	5.456631184	O08557
Mitochondria	Ddah2	dimethylarginine dimethylaminohydrola	23.29975	22.66143	21.22722	22.70776	28.02327	27.7145	28.00072	27.64231	27.64423	27.94514	28.13863	27.83623	+	+	+	4.673597195	5.371157646	4.6877778	5.41701889	Q6MG60
Mitochondria	Dld	dihydrolipoamide dehydrogenase	23.92097	24.04962	22.80157	21.88935	26.20184	26.41653	26.99118	25.09442	26.70147	27.07623	27.76743	26.77543	+	+	+	2.460503457	3.010614395	3.351757904	3.91476059	Q6P6R2
Mitochondria	Fabp3	fatty acid binding protein 3, muscle and	22.55892	21.66856	23.0101	22.98128	29.82578	29.16421	29.60991	29.54051	29.77711	29.28299	29.34714	29.70223	+	+	+	6.047283079	6.980387211	6.084071639	6.972655773	P07483
Mitochondria	Fasn	fatty acid synthase	22.06513	22.75742	21.50127	23.12142	30.91606	31.26668	31.12854	31.29532	31.35689	30.7332	31.50649	31.14108	+	+	+	6.430392094	8.790342331	6.258849722	8.823108196	P12785
Mitochondria	Gars	glycyl-tRNA synthetase	22.63246	22.32798	22.307	23.11946	27.14161	27.45435	26.60498	26.56401	27.18518	26.86219	25.82352	26.95071	+	+	+	5.279154417	4.344511509	4.592384709	4.108675957	G3V7G8;Q5I0G4
Mitochondria	Gbas	glioblastoma amplified sequence	22.77832	21.23619	21.46326	22.07385	25.21423	24.70398	25.38995	22.37246	23.20241	25.69312	25.60487	25.67239	+	+	+	1.75875012	2.532251835	2.377164547	3.155293941	Q5RK08
Mitochondria	Gdap1l1	ganglioside-induced differentiation-assc	22.08697	21.30347	22.67425	22.6592	25.84585	26.53754	26.5366	26.85591	25.65682	26.2536	25.82811	27.07999	+	+	+	4.478871141	4.263000488	3.946019144	4.023658276	B4F774
Mitochondria	Glod4	glyoxalase domain containing 4	23.02513	21.95011	22.24585	21.56747	26.98316	27.01786	26.93472	26.94893	27.16116	27.50885	26.87514	27.33373	+	+	+	5.328310016	4.774028301	5.239578453	5.022576332	Q5I0D1
Mitochondria	Got2	glutamic-oxaloacetic transaminase 2, m	24.40314	23.47424	26.28877	25.862	30.65728	30.02314	30.53816	31.05232	30.304	30.18281	30.8181	30.34223	+	+	+	3.727295512	5.560688496	3.726181158	5.404751778	P00507
Mitochondria	Gpd1	glycerol-3-phosphate dehydrogenase 1	22.66509	23.23545	22.77169	22.4852	27.98994	28.44387	27.9712	28.33189	28.05822	28.49541	28.73276	28.58255	+	+	+	6.766807179	5.394873142	6.700617989	5.677881718	O35077
Mitochondria	Gpx4	glutathione peroxidase 4 (phospholipid	22.61347	22.88198	25.89615	22.93862	26.88402	26.95438	26.77467	26.85887	26.83509	26.86018	26.99281	27.03779	+	+	+	2.263177156	3.285429478	2.300360129	3.348908424	6970-2
Mitochondria	Hadha	hydroxyacyl-Coenzyme A dehydrogenas	22.48696	22.10999	21.34563	23.14224	25.85241	23.25314	25.46448	25.89793	25.17619	24.5242	25.36406	26.05372	+	+	+	2.09107851	2.845785618	3.070401213	3.008341312	Q64428
Mitochondria	Hagh	hydroxyacylglutathione hydrolase	23.00617	21.91275	23.11949	22.59546	27.24	26.13122	26.49167	26.50953	26.60045	25.78821	26.30482	26.26574	+	+	+	4.4683897	3.934635639	4.513780087	3.581336498	F1LQI1;O35952;O35952-2
Mitochondria	Hrsp12	heat-responsive protein 12	22.98159	22.89736	21.13108	22.95019	28.78718	28.1358	28.60417	28.21067	29.22228	28.32436	28.50025	29.02514	+	+	+	4.774519709	5.94439888	4.803425368	6.277950764	P52759
Mitochondria	Hsd17b10	hydroxysteroid (17-beta) dehydrogenas	22.68774	23.49826	22.15969	23.25545	24.46731	25.73854	24.9478	25.23206	25.37382	25.98325	25.3863	25.1553	+	+	+	2.812272856	2.196140766	3.501764191	2.574381351	B0BMW2;O70351
Mitochondria	Hsd17b4	hydroxysteroid (17-beta) dehydrogenas	21.64645	22.95529	23.59817	21.74673	29.07539	28.04062	28.79582	28.2947	28.34456	28.59616	28.65089	28.74858	+	+	+	4.573558521	6.064969063	4.821310529	6.098389149	P97852
Mitochondria	Hspa9	heat shock 70kDa protein 9 (mortalin)	22.7832	20.94583	21.99809	21.90751	27.27186	27.70754	27.8262	27.24309	27.29946	28.33279	28.2597	27.82608	+	+	+	5.052899465	5.603517532	4.993735158	6.020853519	F1M953;P48721
Mitochondria	Hspd1	heat shock 60kDa protein 1 (chaperonin	22.46961	23.41105	22.5711	23.25471	27.1898	26.84458	27.66465	26.96532	27.57162	22.42692	27.96868	27.22011	+	+	+	5.116974823	4.239470959	1.362846787	3.370226383	P63039
Mitochondria	Hspe1	heat shock 10kDa protein 1 (chaperonin	23.18152	20.89633	22.8946	22.46197	30.52477	29.69839	30.05517	29.42934	30.6121	29.8138	29.82479	30.05853	+	+	+	4.984409698	7.568315029	5.122408982	7.718702316	P26772

Supplementary Table 2. List of proteins significantly enriched in both immunoisolated FEZ1 and Kinesin-1 containing vesicles by quantitative mass spectrometry

Group	Gene Symbol	Gene Name	log LFQ intensity IP1 Control	log LFQ intensity IP2 Control	log LFQ intensity IP3 Control	log LFQ intensity IP4 Control	log LFQ intensity IP1 Kinesin-1	log LFQ intensity IP2 Kinesin-1	log LFQ intensity IP3 Kinesin-1	log LFQ intensity IP4 Kinesin-1	log LFQ intensity IP1 FEZ1	log LFQ intensity IP2 FEZ1	log LFQ intensity IP3 FEZ1	log LFQ intensity IP4 FEZ1	t-test Significant Kinesin-1 IP	t-test Significant FEZ1 IP	t-test Significant Kinesin-1 and FEZ1 IP	-Log t-test p value Kinesin-1 IP	t-test Difference Kinesin-1 IP	-Log t-test p value FEZ1 IP	t-test Difference FEZ1 IP	Majority UniProt protein IDs
Mitochondria	Idh1	isocitrate dehydrogenase 1 (NADP+), so	21.86877	22.72552	22.60801	22.45074	28.24109	25.40724	25.91645	25.24282	27.44695	25.80804	26.00129	24.14942	+	+	+	2.72112322	3.78864193	2.568316377	3.438167095	P41562
Mitochondria	Idh3a	isocitrate dehydrogenase 3 (NAD+) alph	23.76502	22.55681	21.97014	23.34543	29.33149	29.57404	29.48506	29.41199	29.28793	29.78165	29.85988	29.66743	+	+	+	5.450733136	6.541297913	5.426538907	6.73987484	F1LNF7;Q99NA5
Mitochondria	Idh3B	isocitrate dehydrogenase 3 (NAD+) beta	23.79357	22.57363	23.62564	22.14545	28.22798	28.11907	27.44293	26.63353	27.78101	28.13448	27.29448	26.53512	+	+	+	3.810548807	4.571305275	3.783334038	4.401698112	Q68FX0
Mitochondria	Idh3g	isocitrate dehydrogenase 3 (NAD+) gam	22.4666	22.22256	23.22235	22.71842	27.16385	26.7415	27.29457	25.63978	27.14763	27.43006	27.32457	26.7685	+	+	+	4.077734716	4.052438736	5.643812388	4.510208607	Q5XIJ3;P41565
Mitochondria	Immt	inner membrane protein, mitochondrial	21.72132	22.21646	22.91028	21.9463	28.24223	27.99814	27.85436	26.73802	28.26696	28.253	28.70464	27.68953	+	+	+	4.909077308	5.509597778	5.751298793	6.029941082	Q3KR86
Mitochondria	Ldha	lactate dehydrogenase A	23.07634	27.88268	30.16459	28.93258	32.06551	31.11768	32.25124	32.2864	32.17694	31.41523	32.3525	32.16042	+	+	+	1.508378217	4.41616106	1.553866168	4.512226582	P04642;M0RBY5
Mitochondria	Ldhb	lactate dehydrogenase B	27.58749	27.56892	29.2511	29.20553	32.36147	30.66899	32.20781	32.1556	32.35269	31.57511	32.4345	32.1944	+	+	+	2.845174368	3.445206642	3.459207086	3.735913277	P42123
Mitochondria	Letm1	leucine zipper-EF-hand containing trans	23.33514	23.01386	23.1444	23.24468	27.24291	27.69192	26.99852	27.40876	27.11038	27.58613	27.98011	27.47653	+	+	+	6.646746306	4.151006222	6.319986006	4.353768349	Q5XIN6
Mitochondria	LOC10036	rCG31129-like	23.66256	22.58369	23.30852	23.85146	27.54995	27.57138	26.99108	27.84452	26.23244	27.38591	27.0239	27.51465	+	+	+	4.787415535	4.137674809	4.026845411	3.687669754	B5DEL8;F7FG98
Mitochondria	LOC10036	NADH dehydrogenase (ubiquinone) 1 be	22.53804	22.25381	22.18168	22.55075	27.8825	28.06343	27.449	27.77687	27.57413	28.74762	28.34554	27.75557	+	+	+	7.343965755	5.411878586	5.992570331	5.724642754	F1LPG5;F1M7T1
Mitochondria	Mdh1	malate dehydrogenase 1, NAD (soluble)	29.39379	29.82772	30.29625	29.32279	32.71559	32.0179	32.55467	32.48815	33.00872	31.38448	32.91969	32.66793	+	+	+	4.265030975	2.733942032	3.142498011	2.785065651	O88989
Mitochondria	Mdh2	malate dehydrogenase 2, NAD (mitocho	21.96236	23.23315	21.31687	23.05491	31.3689	31.51625	31.92047	31.73977	31.75824	31.88453	32.01172	31.97011	+	+	+	5.944172063	9.244523048	6.087487256	9.514324665	P04636
Mitochondria	MPC2	mitochondrial pyruvate carrier 2	22.88044	22.27919	21.77013	22.81427	25.83819	26.49919	25.03271	25.82673	23.7525	26.49537	24.96221	25.94315	+	+	+	3.831989533	3.363197327	2.316697303	2.852300644	P38718;P38718-2
Mitochondria	Mtch2	mitochondrial carrier homolog 2 (C. eleg	23.0262	20.33653	23.32124	22.99478	27.30348	27.33262	27.09869	27.44372	26.68646	27.22536	27.6973	27.66323	+	+	+	3.354488691	4.87493515	3.251038861	4.898397923	B0BN52
Mitochondria	Ndufa10	NADH dehydrogenase (ubiquinone) 1 al	21.61378	23.27771	22.01679	22.02885	27.41724	28.11927	28.29167	28.30038	27.84948	28.62742	28.50047	28.55775	+	+	+	5.063365066	5.797856808	5.298900404	6.14950037	Q561S0
Mitochondria	Ndufa11	NADH dehydrogenase (ubiquinone) 1 al	22.72778	23.92419	22.97728	22.43132	26.45681	26.4845	27.49968	27.34655	26.13581	26.77404	27.83545	27.29789	+	+	+	4.04347493	3.931741238	3.75703024	3.99565649	Q80W89
Mitochondria	Ndufa12	NADH dehydrogenase (ubiquinone) 1 al	22.79914	22.60646	21.5941	21.21714	27.05982	26.52446	26.71568	24.83137	26.95116	26.51431	26.4587	26.9983	+	+	+	3.283057218	4.228619099	4.567310426	4.676403522	F1LXA0
Mitochondria	Ndufa13	NADH dehydrogenase (ubiquinone) 1 al	22.90024	22.40345	23.20324	23.11061	28.00443	28.37898	27.79854	27.603	27.81452	28.4545	28.16825	27.87666	+	+	+	6.080255787	5.041849136	6.280917114	5.174096107	D3ZE15;F1LZC5
Mitochondria	Ndufa4	NADH dehydrogenase (ubiquinone) 1 al	23.03315	22.25059	22.35194	22.44169	29.58077	30.49943	30.35448	30.76738	30.74126	28.36453	29.94252	31.10493	+	+	+	6.584589948	7.781172276	4.665535457	7.518966675	B2RZD6
Mitochondria	Ndufa5	NADH dehydrogenase (ubiquinone) 1 al	21.5413	23.2535	23.27015	23.3292	27.06784	26.86455	27.03454	26.9629	27.47499	27.42606	27.48347	27.19798	+	+	+	4.092363515	4.1339221	4.312096872	4.547088623	Q63362
Mitochondria	Ndufa6	NADH dehydrogenase (ubiquinone) 1 al	22.36708	21.56543	21.63465	21.51284	27.78961	26.55836	26.39833	26.82043	27.14131	26.43476	26.46205	26.87385	+	+	+	5.048606219	5.121682644	5.839414972	4.957992077	D4A3V2
Mitochondria	Ndufa8	NADH dehydrogenase (ubiquinone) 1 al	21.91608	21.72352	23.09325	21.68244	25.21223	22.24204	26.14143	25.54569	26.01514	24.71717	26.82335	26.28433	+	+	+	1.55186139	2.681524277	3.343496811	3.856175423	Q7TP78
Mitochondria	Ndufa9	NADH dehydrogenase (ubiquinone) 1 al	22.21417	21.08693	22.21591	22.91601	27.17503	26.74317	27.24164	25.92121	27.22849	27.14715	27.01744	27.30756	+	+	+	4.138005903	4.662004948	4.93542527	5.066904545	Q5BK63
Mitochondria	Ndufb10	NADH dehydrogenase (ubiquinone) 1 be	21.26328	22.92461	23.4551	21.9727	26.81812	27.14822	27.3919	27.33722	27.53089	27.26749	27.07092	27.10988	+	+	+	4.094578916	4.76994133	4.159711387	4.840871334	D4A0T0
Mitochondria	Ndufb5	NADH dehydrogenase (ubiquinone) 1 be	22.71108	22.17446	21.52147	23.05497	27.28489	26.80907	27.93624	27.1037	27.24943	27.04656	27.74919	27.60774	+	+	+	4.685116577	4.917979717	5.006371702	5.047736645	D4A565
Mitochondria	Ndufb6	NADH dehydrogenase (ubiquinone) 1 be	22.94356	22.71814	23.137	22.4467	26.47522	25.49436	26.32982	25.5809	25.22328	25.78124	26.2551	25.67406	+	+	+	4.427200142	3.158724308	4.540054559	2.922072411	D3ZZ21
Mitochondria	Ndufb7	NADH dehydrogenase (ubiquinone) 1 be	21.70996	23.73297	21.92809	22.93142	27.23717	25.80331	27.61828	26.80993	27.56122	26.18192	27.53734	27.05061	+	+	+	3.38354879	4.291565895	3.668390488	4.507166386	D3ZLT1
Mitochondria	Ndufc2	NADH dehydrogenase (ubiquinone) 1, su	22.9341	22.8324	22.9454	21.72949	27.4128	27.45623	27.4538	27.16663	27.3501	27.54082	27.37667	27.31398	+	+	+	5.378228222	4.762013435	5.422795456	4.785039902	Q5PQZ9
Mitochondria	Ndufs1	NADH dehydrogenase (ubiquinone) Fe-S	21.49462	22.90338	22.41475	21.87177	29.62312	29.50996	29.5796	29.5881	29.20793	29.41943	29.74429	29.69408	+	+	+	6.455348951	7.404067039	6.245842018	7.345303535	Q66HF1
Mitochondria	Ndufs2	NADH dehydrogenase (ubiquinone) Fe-S	22.72301	21.93403	22.18526	22.9327	27.32432	22.20392	25.86264	26.23759	27.44648	26.88518	27.03149	26.31917	+	+	+	1.39625601	2.963366032	5.013022908	4.476831913	Q641Y2
Mitochondria	Ndufs3	NADH dehydrogenase (ubiquinone) Fe-S	21.80411	22.83761	22.33266	23.32182	27.87109	28.21646	28.21006	28.1504	27.86237	28.64389	28.2768	28.33748	+	+	+	5.492966227	5.537952423	5.369705612	5.706080914	D3ZG43
Mitochondria	Ndufs4	NADH dehydrogenase (ubiquinone) Fe-S	21.29108	21.86665	21.76433	22.89571	26.66126	25.89569	27.85798	27.19526	26.74047	26.84026	27.86254	27.78669	+	+	+	4.042514204	4.948106289	4.661417268	5.353048801	Q5XIF3
Mitochondria	Ndufs6	NADH dehydrogenase (ubiquinone) Fe-S	22.312	22.05508	22.50469	22.99749	27.4008	28.11823	28.09869	27.66811	28.4477	28.02739	28.5437	27.81714	+	+	+	6.021163292	5.354141712	6.216274016	5.741669178	D3ZCZ9;G3V8B9
Mitochondria	Ndufs8	NADH dehydrogenase (ubiquinone) Fe-S	21.49215	22.97944	22.64466	23.15007	25.48791	25.67603	26.43493	25.72338	25.86585	25.47187	25.96276	25.09575	+	+	+	3.5813631	3.263983727	3.431030027	3.03248167	B0BNE6
Mitochondria	Ndufv2	NADH dehydrogenase (ubiquinone) flav	22.75981	22.23689	21.42052	22.16686	26.48777	26.36843	26.68833	26.80292	26.72754	26.78905	26.76433	27.06682	+	+	+	5.288509856	4.440841198	5.481806207	4.690916061	P19234
Mitochondria	Nipsnap1	nipsnap homolog 1 (C. elegans)	21.51188	22.10069	22.23787	23.12327	26.92116	26.84038	27.36545	26.83882	27.16385	27.02686	27.16902	27.56667	+	+	+	4.959086252	4.748024464	5.10794298	4.988170147	G3V728
Mitochondria	Nme2	non-metastatic cells 1, protein (NM23A)	22.76127	23.72764	21.96439	23.76652	32.3037	31.10468	32.06137	30.55215	32.06955	30.53102	31.97524	31.73763	+	+	+	5.117798581	8.450521469	5.297014989	8.523407936	P19804
Mitochondria	Nudt2	nudix (nucleoside diphosphate linked m	23.03781	22.99685	22.73162	21.44352	26.06885	24.87643	26.5353	25.68016	26.54965	25.70549	26.16189	26.25059	+	+	+	3.125576779	3.237736702	3.900716923	3.614454746	Q6PEC0
Mitochondria	Ogdh	oxoglutarate (alpha-ketoglutarate) dehy	21.73677	21.14503	21.79937	22.8601	27.53208	26.51967	27.66743	27.35448	27.18386	27.49755	27.79613	27.58484	+	+	+	4.740327176	5.383096218	5.234206657	5.63027668	Q5XI78
Mitochondria	Ogt	O-linked N-acetylglucosamine (GlcNAc)	23.10665	21.84666	27.12869	23.5908	27.18357	27.8106	28.25165	28.14326	26.93888	27.68358	27.83714	28.26419	+	+	+	1.836935415	3.929071426	1.747183773	3.762745857	G3V6F4;P56558
Mitochondria	Oxr1	oxidation resistance 1	21.55172	24.2267	22.14816	23.014	27.6392	28.02179	28.23768	28.17102	28.20046	28.40011	28.5099	28.25701	+	+	+	3.940100629	5.28227663	4.132307674	5.606724739	Q4V8B0
Mitochondria	Park7	Parkinson disease (autosomal recessive,	21.53484	26.7196	24.21804	27.85347	30.28824	29.74448	29.77117	30.04986	30.32335	30.11595	30.14844	29.83825	+	+	+	1.870739759	4.881949902	1.927226957	5.025007725	O88767
Mitochondria	Pdha1	pyruvate dehydrogenase (lipoamide) alp	21.51112	22.13674	21.87783	22.24897	27.68572	27.03507	28.0835	27.55443	27.00967	28.04463	27.08881	27.75856	+	+	+	6.095268996	5.64601469	5.770345696	5.531755924	D4A5G8;P26284;F7FKI5
Mitochondria	Pdhb	pyruvate dehydrogenase (lipoamide) be	22.13108	22.59154	21.09372	22.12144	28.99289	28.54374	28.61933	28.65506	28.85955	28.62139	29.14421	28.37956	+	+	+	6.025726841	6.718308926	5.860889808	6.766735077	P49432
Mitochondria	Phb	prohibitin	21.9348	22.52727	22.03916	22.02696	28.72952	28.31596	28.86777	28.40446	28.39223	28.64134	28.96218	28.46665	+	+	+	7.399090155	6.44738102	7.453810574	6.483552933	P67779
Mitochondria	Phb2	prohibitin 2	22.21622	22.29507	22.91859	22.94707	28.42842	28.60474	28.39002	28.34287	28.23918	28.52477	28.43806	28.46338	+	+	+	6.918021923	5.84727335	6.889843545	5.822109222	Q5XIH7
Mitochondria	Phyhipl	phytanoyl-CoA 2-hydroxylase interactin	21.97075	22.01033	22.97777	23.58945	26.9651	26.71816	26.86915	26.81641	27.40576	25.83219	26.2678	27.63851	+	+	+	4.379761182	4.205130577	3.391955386	4.148991108	Q6AYN4;Q6AYN4-2
Mitochondria	Por	P450 (cytochrome) oxidoreductase	23.09003	21.84644	23.72283	23.45262	28.72533	29.07798	27.91165	28.02364	29.06376	28.78169	28.30743	27.98283	+	+	+	4.429938918	5.406672478	4.581223123	5.505949974	P00388
Mitochondria	Ppid	peptidylprolyl isomerase D	21.68742	22.23534	22.97718	21.59679	27.19864	26.58219	27.68191	27.85394	27.16452	27.13457	27.32834	27.52761	+	+	+	4.734243974	5.204987049	5.365522192	5.164577961	Q6DGG0;M0RB67
Mitochondria	Prdx1	peroxiredoxin 1	25.60113	22.66442	26.17514	27.57333	30.51343	29.98536	30.18683	30.08487	30.77148	29.39793	30.3333	30.06997	+	+	+	2.392173693	4.689114094	2.305849129	4.639662743	Q63716
Mitochondria	Prdx2	peroxiredoxin 2	27.23736	28.54742	27.44024	28.30791	31.26925	30.53631	31.29285	30.69495	31.30867	30.13357	31.47237	30.6453	+	+	+	3.741152903	3.065108299	3.287593053	3.006747723	P35704
Mitochondria	Prdx3	peroxiredoxin 3	21.14808	22.3373	23.10653	21.6891	27.46793	27.36336	27.60675	26.80772	27.4415	27.19009	27.43587	26.99032	+	+	+	4.577258466	5.241189003	4.673498638	5.194194794	G3V7I0;Q9Z0V6
Mitochondria	Prdx5	peroxiredoxin 5	21.611	22.54061	27.76218	27.10809	30.36818	29.37252	30.36713	29.71537	30.20385	29.38796	30.36213	29.74703	+	+	+	1.775843192	5.200329304	1.769752616	5.169772148	Q9R063;Q9R063-2;D3ZEN5
Mitochondria	Prdx6	peroxiredoxin 6	22.44677	22.55879	27.35439	27.72709	30.11756	30.46051	30.48884	30.79846	30.26908	30.59246	30.49924	30.79328	+	+	+	2.007059748	5.444582462	2.03569236	5.516752243	O35244
Mitochondria	Prosc	proline synthetase co-transcribed homo	22.49914	22.83989	23.08589	23.68504	25.14876	25.49094	25.55862	25.09007	24.41261	25.2235	25.88395	25.65518	+	+	+	3.778731983	2.294607639	2.834525007	2.266322136	D3ZCA0
Mitochondria	Qdpr	quinoid dihydropteridine reductase	23.97157	24.40398	22.31266	21.99383	27.42262	26.9536	27.44514	27.59911	26.18646	27.783	27.62045	27.24536	+	+	+	3.312121528	4.184607983	2.936438877	4.038310051	P11348
Mitochondria	Sccpdh	saccharopine dehydrogenase (putative)	23.41608	21.77561	22.24398	22.8474	26.85994	27.39428	27.16116	27.17074	27.1866	27.15074	26.98305	26.57204	+	+	+	4.744114353	4.575761795	4.578608384	4.402341843	Q6AY30
Mitochondria	Scp2	sterol carrier protein 2	21.81393	22.04544	22.11273	22.80438	29.88026	29.1347	29.18125	28.86629	29.13607	29.14875	29.27813	29.15634	+	+	+	6.385192615	7.071503639	7.238267019	6.985700607	P11915;F1LQ55;P11915-2
Mitochondria	Sdhb	succinate dehydrogenase complex, subu	21.90523	22.48757	21.41571	21.75395	27.1047	26.71856	26.54896	26.77367	26.53475	26.90564	26.23533	26.81787	+	+	+	5.917251794	4.895853519	5.65246973	4.732782841	P21913
Mitochondria	Sfxn1	sideroflexin 1	23.15713	21.25732	22.16372	21.30392	26.23127	27.15374	27.19131	26.73376	26.421	27.09245	27.08497	26.96961	+	+	+	4.164821172	4.856995106	4.326917265	4.921482086	Q63965
Mitochondria	Sfxn3	sideroflexin 3	22.10454	22.66632	22.92339	22.56011	27.7921	27.9629	28.23457	27.96091	27.58671	27.84194	28.1155	28.15035	+	+	+	6.859527296	5.424028397	6.551692803	5.360033512	Q9JHY2;G3V804

Supplementary Table 2. List of proteins significantly enriched in both immunoisolated FEZ1 and Kinesin-1 containing vesicles by quantitative mass spectrometry

Group	Gene Symbol	Gene Name	log LFQ intensity IP1 Control	log LFQ intensity IP2 Control	log LFQ intensity IP3 Control	log LFQ intensity IP4 Control	log LFQ intensity IP1 Kinesin-1	log LFQ intensity IP2 Kinesin-1	log LFQ intensity IP3 Kinesin-1	log LFQ intensity IP4 Kinesin-1	log LFQ intensity IP1 FEZ1	log LFQ intensity IP2 FEZ1	log LFQ intensity IP3 FEZ1	log LFQ intensity IP4 FEZ1	t-test Significant Kinesin-1 IP	t-test Significant FEZ1 IP	t-test Significant Kinesin-1 and FEZ1 IP	-Log t-test p value Kinesin-1 IP	t-test Difference Kinesin-1 IP	-Log t-test p value FEZ1 IP	t-test Difference FEZ1 IP	Majority UniProt protein IDs
Mitochondria	Slc25a11	solute carrier family 25 (mitochondrial c	22.13023	22.92552	22.90075	22.11849	27.77831	27.68933	28.0704	27.52694	26.98044	27.88832	28.34287	28.32002	+	+	+	6.070367297	5.247497082	5.026928831	5.364162922	G3V6H5;P97700
Mitochondria	Snd1	staphylococcal nuclease and tudor dom	22.81871	23.58495	22.05499	23.15535	25.40315	25.15383	25.17828	25.49158	25.28651	25.14262	25.30859	26.21862	+	+	+	3.438476257	2.403213024	3.150823365	2.585587978	Q66X93;D4A8Y5
Mitochondria	Sod1	superoxide dismutase 1, soluble	21.99918	22.58685	23.21034	23.0428	31.13913	30.33756	31.42036	30.21454	31.82862	30.06252	29.66307	30.10687	+	+	+	6.004953876	8.068105698	5.071682304	7.705478668	P07632
Mitochondria	Sod2	superoxide dismutase 2, mitochondrial	22.93734	23.9853	22.35497	22.80671	28.27875	26.89804	28.04422	27.99976	28.23453	27.78849	28.43893	28.32243	+	+	+	4.320264054	4.784109592	5.059383407	5.175011635	P07895
Mitochondria	Sucla2	succinate-CoA ligase, ADP-forming, beta	21.21451	22.75214	22.75254	26.37815	28.03742	27.35279	28.20341	27.32053	28.44727	27.40048	27.89423	27.49823	+	+	+	2.136348153	4.454201698	2.169020274	4.53571558	F1LM47
Mitochondria	Tomm70a	translocase of outer mitochondrial mem	22.33063	21.89025	22.22205	24.06421	26.66669	26.33296	27.05951	25.38235	26.54569	26.75748	27.62756	26.5414	+	+	+	3.077992284	3.733591557	3.593382718	4.2412467	Q75Q39
Mitochondria	Uqcrb	similar to ubiquinol-cytochrome c reduc	22.68569	21.5282	23.8772	23.38954	28.83455	28.27222	28.35321	28.35372	28.56304	28.74198	28.66696	28.39738	+	+	+	4.386524694	5.583264351	4.498217084	5.722177505	B2RYS2
Mitochondria	Uqcrc1	ubiquinol-cytochrome c reductase core	22.21903	22.89072	21.9828	23.1667	28.01537	27.58563	27.91108	27.61098	27.58542	25.62606	28.38574	28.67337	+	+	+	5.651434862	5.215952873	3.288312293	5.002836227	Q68FY0
Mitochondria	Uqcrc2	ubiquinol-cytochrome c reductase core	22.1515	22.37547	22.07816	21.15846	29.36876	29.44607	29.62892	29.19315	29.39748	29.67667	29.74327	29.65932	+	+	+	6.707563288	7.468326092	6.818165174	7.67828846	P32551
Mitochondria	Uqcrfs1	ubiquinol-cytochrome c reductase, Riesl	22.93019	22.08842	23.16522	23.01248	27.4186	22.47456	27.50074	26.09384	27.55815	26.74227	26.64003	22.22013	+	+	+	1.365346314	3.072857857	1.290243924	2.991068363	P20788
Mitochondria	Vdac2	voltage-dependent anion channel 2	26.2009	27.76034	28.76196	23.01426	30.0966	30.2247	30.64186	30.37233	30.39202	30.45699	30.75223	30.71555	+	+	+	1.670683453	3.899507999	1.780466439	4.144833565	P81155
Mitochondria	Vdac3	voltage-dependent anion channel 3	22.99249	23.50657	22.16272	22.60271	29.6655	29.63855	29.79849	30.15086	29.18182	29.7774	30.01465	29.66	+	+	+	6.312550271	6.997230053	6.046212548	6.842348576	Q9R1Z0-2
Myelin associated	Mag	myelin associated glycoprotein	23.39559	21.75778	21.7562	21.72633	28.22885	24.91031	27.82475	27.88571	28.09028	24.84637	28.17722	27.4174	+	+	+	2.927521269	5.053427219	2.873006745	4.973838806	G3V9B3;P07722-2
Myelin associated	Mbp	myelin basic protein	21.89342	29.37598	21.50915	22.4435	33.36763	32.32948	31.75923	31.03702	33.38831	32.54779	32.30449	32.24167	+	+	+	2.297236388	8.317827702	2.46707629	8.815052509	P02688;P02688-2
Myelin associated	Mog	myelin oligodendrocyte glycoprotein	22.80291	23.46428	22.11611	21.86693	28.70203	27.6569	29.20851	28.17322	29.08993	28.16964	29.2651	28.85317	+	+	+	4.682019646	5.872608185	5.174146124	6.281903744	Q63345;Q6MFX9
Myelin associated	Omg	oligodendrocyte myelin glycoprotein	22.75241	22.98739	22.48608	21.82959	29.31683	27.96163	28.90219	28.54477	29.02385	28.13536	28.85923	29.29356	+	+	+	5.453480992	6.167487144	5.715157045	6.314130306	F7EYB9
Myelin associated	Plp1	proteolipid protein 1	27.49534	28.51796	29.7877	29.28633	32.62379	31.47508	32.38782	32.24476	33.09558	32.30343	32.85047	32.3912	+	+	+	3.059687648	3.411035061	3.468460593	3.88834095	P60203
Nuclear-Cytosol Transport	Ipo5	importin 5	22.47047	21.44403	23.80724	23.01186	26.39787	27.93202	27.19517	27.07163	26.9283	26.93629	26.99259	26.98934	+	+	+	3.569998385	4.465769291	3.872424863	4.27822876	D4A781;M0RB74
Nuclear-Cytosol Transport	Ipo7	importin 7	23.16048	21.25636	23.06116	24.15699	26.29243	27.13819	26.70872	26.231	26.72598	25.82053	26.87091	27.17331	+	+	+	2.923429453	3.68383646	2.851071522	3.738936901	D4AE96
Nuclear-Cytosol Transport	Ipo9	importin 9	23.40635	22.90222	23.87893	21.51044	25.10133	26.90495	25.25003	25.44812	25.80336	24.98498	26.01502	26.7252	+	+	+	2.232256317	2.7516222	2.493634646	2.95765543	D4A857
Nuclear-Cytosol Transport	Kpnb1	karyopherin (importin) beta 1	22.98147	21.9011	26.3286	23.62917	28.42838	27.96598	28.10679	28.41679	28.30552	27.57946	28.62801	28.99345	+	+	+	2.504166653	4.519402027	2.485148385	4.666524887	F2Z3Q8;P52296
Nuclear-Cytosol Transport	NUTF2	nuclear transport factor 2	22.80129	22.59649	22.78062	22.91535	27.33986	26.07696	27.34291	26.59263	27.24136	26.42023	27.48738	27.63519	+	+	+	4.867151252	4.064654827	5.398240949	4.422607422	P61972
Nuclear-Cytosol Transport	Tnpo2	transportin 2	22.7804	22.54622	22.68608	23.0779	25.49741	25.86971	25.40051	25.47039	24.64537	25.09772	24.68089	25.67282	+	+	+	5.7337564	2.786854267	3.841874012	2.251550198	D3ZER6
Nuclear-Cytosol Transport	Xpo1	exportin 1 (CRM1 homolog, yeast)	22.74076	21.99662	21.07854	22.97369	25.36256	28.36774	26.6983	26.95903	26.88379	27.13369	26.37039	27.51503	+	+	+	3.092002191	4.649502277	4.175188658	4.778321266	Q80U96
Nucleic Acid Metabolism	Ahcyl1	adenosylhomocysteinase-like 1	22.36585	22.79778	22.3258	22.97701	28.7939	27.31941	29.04755	28.65918	27.50998	28.25341	29.59203	29.78603	+	+	+	5.069501504	5.838401794	4.442294424	6.16875267	D4A5X8
Nucleic Acid Metabolism	Cmpk1	cytidine monophosphate (UMP-CMP) ki	22.94073	22.15901	22.18125	22.92122	28.52917	27.39836	28.13055	27.77467	27.70695	27.4475	28.24581	27.95505	+	+	+	5.507203421	5.407638073	5.862872443	5.288275242	Q4KM73
Nucleic Acid Metabolism	Crip2	cysteine-rich protein 2	22.72209	22.07096	22.00389	21.2362	27.17636	28.30783	27.88658	27.4275	27.4434	28.16969	27.98544	27.91627	+	+	+	5.161670436	5.691282272	5.607952321	5.870414257	P36201
Nucleic Acid Metabolism	Ddb1	damage-specific DNA binding protein 1,	21.23345	21.65841	23.66074	22.24431	26.6284	25.86825	26.512	23.4047	27.17731	26.14032	26.40513	26.1875	+	+	+	1.997457482	3.404111862	3.491541879	4.278338909	G3V8T4;Q9ESW0
Nucleic Acid Metabolism	Itpa	inosine triphosphatase (nucleoside triph	21.14447	23.50743	23.81374	22.05359	26.38471	26.14104	27.44632	27.53749	26.40831	26.47826	27.46987	27.80888	+	+	+	2.971107235	4.247585297	3.067281872	4.411525726	D3ZW55
Nucleic Acid Metabolism	Nme1	non-metastatic cells 1, protein (NM23A)	21.56306	23.08928	23.70174	22.23605	28.92813	26.98696	27.93089	27.51269	25.85766	27.70359	27.77367	29.07561	+	+	+	3.784833318	5.192133427	3.055196625	4.955098152	Q05982
Nucleic Acid Metabolism	Pabpc1	poly(A) binding protein, cytoplasmic pse	23.06404	22.97033	26.07989	23.50227	27.18782	28.14521	28.5927	28.68562	28.22191	28.33224	28.14448	27.93988	+	+	+	2.713583634	4.248705864	2.922200763	4.255493164	Q9EPH8
Nucleic Acid Metabolism	Pnp	nucleoside phosphorylase	22.5836	23.12315	22.6195	22.95605	28.4545	28.75805	28.32753	28.74375	28.46844	28.99863	28.60406	28.78144	+	+	+	7.362011209	5.750382423	7.351160254	5.892566681	P85973;D3ZXK9
Phosphatase	Acp1	acid phosphatase 1, soluble	23.19572	20.00192	23.31195	21.85423	27.258	27.38435	28.48232	27.97645	27.83617	26.89712	28.52776	28.03905	+	+	+	3.346811303	5.684323788	3.305127273	5.734070778	P41498
Phosphatase	Dusp3	dual specificity phosphatase 3	23.11716	20.95454	26.81348	27.1546	27.95959	28.97883	28.37613	28.7498	27.82293	28.31562	28.27462	28.49998	+	+	+	1.418360899	4.006139755	1.316395657	3.718341351	G3V9L3
Phosphatase	Lhpp	phospholysine phosphohistidine inorgai	21.79099	22.95409	22.8157	22.61957	26.01965	25.52373	25.80287	25.52397	24.99662	25.95943	26.42337	25.23396	+	+	+	4.486627998	3.172463417	3.509637247	3.108258724	Q5I0D5
Phosphatase	Lppr4	plasticity related gene 1	23.60363	23.21795	22.87764	21.31694	28.07994	26.52634	27.19282	26.19263	28.44455	26.3806	27.80403	26.81115	+	+	+	3.203964675	4.243890762	3.274506452	4.606040001	G3V864;Q7TMB7
Phosphatase	Pdxp	pyridoxal (pyridoxine, vitamin B6) phos	21.94781	21.33543	23.18098	23.08903	28.09808	27.63679	28.19235	28.08593	27.91205	27.8031	28.42158	28.25444	+	+	+	4.701721591	5.614973545	4.71282114	5.709480286	Q8VD52;M0R416;B2GV79
Phosphatase	Ppap2b	phosphatidic acid phosphatase type 2B	23.62568	22.53077	22.17184	22.77787	28.18239	29.01725	28.55486	28.99232	28.57604	28.99445	28.55731	28.66455	+	+	+	5.434255723	5.91016674	5.751111347	5.921548843	P97544;Q6IMX4
Phosphatase	Ppm1e	protein phosphatase 1E (PP2C domain c	22.07491	22.34352	22.57599	21.86442	26.83858	25.83726	26.77191	27.25142	27.16461	26.39143	27.63027	27.68839	+	+	+	4.942396899	4.460077763	5.227835391	5.003963947	Q80Z30
Phosphatase	Ppp1ca	protein phosphatase 1, catalytic subunit	20.58312	21.41034	26.44623	29.2658	29.70185	29.10772	29.69927	29.84542	29.94684	29.49245	30.18896	30.01425	+	+	+	1.324807777	5.162193775	1.416372573	5.484255791	P62138
Phosphatase	Ppp1r1b	protein phosphatase 1, regulatory (inhib	22.78657	21.90435	27.61828	22.86173	27.07837	27.44719	26.8087	26.99776	27.55091	26.38588	28.09637	27.67357	+	+	+	1.349912323	3.29027319	1.450623215	3.633951664	Q6J4I0
Phosphatase	Ppp1r7	protein phosphatase 1, regulatory (inhib	24.03614	22.25133	21.38964	22.01499	27.21123	26.59392	26.44419	24.53789	26.95936	26.61119	26.40331	25.58052	+	+	+	2.460518285	3.773784161	3.094135221	3.965573788	Q5HZV9
Phosphatase	Ppp1r9b	protein phosphatase 1, regulatory (inhib	21.63273	22.83788	22.8685	24.92112	28.69149	26.68258	27.9881	27.42838	29.22387	28.57463	28.81293	28.57856	+	+	+	2.923200038	4.632582188	3.750739513	5.732441425	O35274
Phosphatase	Ppp2cb	protein phosphatase 2 (formerly 2A), ca	21.50271	22.29251	28.2035	26.74983	28.69714	28.88826	28.95421	28.58628	29.2377	28.48474	29.24658	29.14141	+	+	+	1.323353847	4.09433651	1.403394521	4.340471745	P62716
Phosphatase	Ppp2r1a	protein phosphatase 2 (formerly 2A), re	21.91633	22.39537	22.74314	22.43382	28.6761	29.42069	28.36181	28.75716	28.39595	27.86697	28.91553	28.81336	+	+	+	6.346054419	6.431776047	6.105567996	6.125785351	Q5XI34
Phosphatase	Ppp2r4	protein phosphatase 2A activator, regul	22.02416	23.75298	22.91407	22.14016	27.03391	26.60442	27.97416	27.89908	27.47707	26.37289	27.84266	28.28339	+	+	+	3.963659634	4.670051575	3.800016095	4.786162376	B2RYQ2
Phosphatase	Ppp3ca	protein phosphatase 3 (formerly 2B), ca	23.36632	28.08887	28.84808	26.77417	30.81886	30.92181	30.73981	30.44579	30.64779	30.56711	31.00899	30.76912	+	+	+	1.761144943	3.962211132	1.769310372	3.978890896	P63329;P63329-2
Phosphatase	Ppp3cb	protein phosphatase 3 (formerly 2B), ca	20.79899	22.62247	22.42856	23.69449	27.55017	27.79458	27.25314	27.29026	27.37393	27.51435	27.17588	27.7254	+	+	+	3.787512235	5.085906982	3.783939877	5.061260223	P20651;F1LMH3
Phosphatase	Ppp3r1	protein phosphatase 3 (formerly 2B), re	22.84584	22.38347	22.21692	27.57715	29.2571	29.90699	29.94531	30.2793	28.73357	29.62167	30.3713	30.4072	+	+	+	2.474175426	6.091328621	2.386060199	6.027588367	P63100-2;P63100
Phosphatase	Ptp4a1	protein tyrosine phosphatase type IVA,	21.19977	21.6959	22.1692	22.31533	25.20396	25.7508	25.40347	25.47874	24.66626	25.33173	25.36179	26.33221	+	+	+	4.906422742	3.61419487	3.810004009	3.57794714	Q78EG7
Phosphatase	Ptplad1	protein tyrosine phosphatase-like A dor	21.61723	23.62512	21.76215	23.79768	27.01372	27.76085	27.30452	27.69797	27.65629	28.04354	27.30678	26.68726	+	+	+	3.618850882	4.743719101	3.452738232	4.722922325	D4ABI7
Phosphatase	Ptpn23	protein tyrosine phosphatase, non-rece	23.1475	22.01199	23.1758	23.11433	25.90866	27.26732	26.97454	26.48844	26.39187	25.89878	25.98079	27.11376	+	+	+	4.041533256	3.797338486	3.916084838	3.483897209	F1M951;O88902
Phosphatase	Ptprd	protein tyrosine phosphatase, receptor	21.65739	23.83592	23.44102	24.16119	27.89747	27.07908	27.48047	26.03729	27.56311	26.79897	27.34215	26.91468	+	+	+	2.863508612	3.849696159	3.240590663	3.880846977	F1M678;F1LM72;F1M2Y2
Phosphatase	Ptprs	protein tyrosine phosphatase, receptor	22.97458	23.90438	23.44886	22.49423	28.82999	28.07694	28.76598	28.37335	28.58857	27.97213	28.72585	28.47181	+	+	+	5.274797595	5.306047916	5.28412843	5.234075546	2;M0R711;M0RA99
Phosphatase	Ptprz1	protein tyrosine phosphatase, receptor-	21.41167	22.46171	21.58583	22.68609	29.62103	28.43575	30.29132	29.45666	29.79538	28.72585	30.53157	31.05355	+	+	+	5.244378976	7.414862633	4.975391613	7.990265846	2;F1LMY3
Protein Metabolism	Aars	alanyl-tRNA synthetase	22.89485	21.63045	22.48937	22.89234	26.92683	24.19731	25.91853	24.87839	27.12021	23.37837	27.09013	27.76528	+	+	+	2.384673282	3.003510475	1.998663604	3.86174345	P50475
Protein Metabolism	Adam22	ADAM metallopeptidase domain 22	21.26802	23.39019	22.56894	22.74611	28.68999	28.17703	28.90624	29.00019	28.99459	28.96022	28.90512	29.08307	+	+	+	4.870338491	6.200044155	5.178277352	6.492432594	M0R8N9;M0R8F5;M0RCU1;
Protein Metabolism	Adam23	ADAM metallopeptidase domain 23	21.88735	21.36219	21.37144	23.32654	26.3615	23.20412	27.53319	27.55326	26.42835	27.6336	27.73512	27.93916	+	+	+	2.002998858	4.176139355	4.102587837	5.447178841	D3ZT36
Protein Metabolism	Capns1	calpain, small subunit 1	31.41629	30.87621	30.52038	30.08322	26.54228	28.7851	27.15788	27.74522	27.37044	28.49507	27.00335	27.28639	+	+	+	2.904425031	-3.16640139	3.491775055	-3.185209274	Q64537;M0RD20
Protein Metabolism	Cst3	cystatin C	22.24048	23.24844	22.64659	22.3023	27.55135	27.6698	27.28233	21.52313	27.6567	28.22343	27.64368	27.4247	+	+	+	1.180109287	3.397199631	5.7028154	5.127676964	P14841
Protein Metabolism	Dpp10	dipeptidyl-peptidase 10	22.5259	22.22827	23.58256	21.60389	29.04846	28.40742	28.79442	28.24767	28.73547	28.47553	29.10485	28.86997	+	+	+	5.007863737	6.139339924	5.180904331	6.311302185	Q6Q629;F1LPW1

Supplementary Table 2. List of proteins significantly enriched in both immunoisolated FEZ1 and Kinesin-1 containing vesicles by quantitative mass spectrometry

Group	Gene Symbol	Gene Name	log LFQ intensity IP1 Control	log LFQ intensity IP2 Control	log LFQ intensity IP3 Control	log LFQ intensity IP4 Control	log LFQ intensity IP1 Kinesin-1	log LFQ intensity IP2 Kinesin-1	log LFQ intensity IP3 Kinesin-1	log LFQ intensity IP4 Kinesin-1	log LFQ intensity IP1 FEZ1	log LFQ intensity IP2 FEZ1	log LFQ intensity IP3 FEZ1	log LFQ intensity IP4 FEZ1	t-test Significant Kinesin-1 IP	t-test Significant FEZ1 IP	t-test Significant Kinesin-1 and FEZ1 IP	-Log t-test p value Kinesin-1 IP	t-test Difference Kinesin-1 IP	-Log t-test p value FEZ1 IP	t-test Difference FEZ1 IP	Majority UniProt protein IDs
Protein Metabolism	Dpp3	dipeptidyl-peptidase 3	22.64968	21.42633	21.6323	21.99308	27.51209	26.6637	28.23859	28.41675	27.06075	27.05817	28.09144	28.20369	+	+	+	4.696615663	5.782436848	5.031192761	5.678164959	O55096
Protein Metabolism	Dtd1	histidyl-tRNA synthetase 2, mitochondri	22.02496	22.77266	21.16934	22.2806	25.76931	26.06036	25.16948	25.70578	26.23708	25.95748	24.58101	25.21494	+	+	+	4.090819771	3.614338875	3.31813436	3.43573761	B0K014;D4A9K3
Protein Metabolism	Eef1b2	eukaryotic translation elongation factor	23.33772	22.57238	22.73351	22.87687	26.4742	26.90633	26.41203	26.9015	25.49732	26.04009	26.60357	26.37312	+	+	+	5.706692085	3.793393135	4.509567175	3.248404026	B5DEN5
Protein Metabolism	Eef1d	eukaryotic translation elongation factor	22.16043	21.39815	23.56323	23.59952	26.2737	26.15813	26.74008	26.36954	26.61583	26.84769	26.72481	26.57414	+	+	+	3.251676377	3.705031395	3.484881001	4.01028347	Q68FR9-2;Q68FR9
Protein Metabolism	Eif5a	eukaryotic translation initiation factor 5	21.74888	22.30086	23.1267	23.78319	28.23201	27.1487	28.19244	28.11436	27.97749	27.56986	27.84769	28.19366	+	+	+	4.239155768	5.181972027	4.484183701	5.157268047	Q3T1J1;G3V7J7
Protein Metabolism	Lxn	latexin	21.49765	23.2883	21.70667	23.35965	27.78063	28.31077	28.10109	28.39395	27.75404	28.29575	28.27315	28.21974	+	+	+	4.47052531	5.683546543	4.476214715	5.672606945	Q64361
Protein Metabolism	Npepps	hypothetical protein FLJ11822; aminope	21.66512	21.97161	22.58112	23.19996	30.1246	29.7164	30.17141	29.94098	30.11483	29.96444	30.22963	30.31743	+	+	+	6.17711702	7.633893013	6.28393209	7.802130699	F1M9V7
Protein Metabolism	Ppia	similar to TRIMCyp; peptidylprolyl isom	26.94748	22.58399	30.09974	30.04609	33.61248	32.44655	33.84903	33.00848	33.25547	32.68489	33.24587	32.54093	+	+	+	1.745622459	5.809810162	1.671761186	5.512463093	P10111
Protein Metabolism	Rpl22	ribosomal protein L22 pseudogene 11; r	22.73832	23.11255	28.02411	23.3704	26.37113	28.03328	27.5093	27.70603	26.50677	27.51616	27.1865	27.48892	+	+	+	1.265940382	3.093587875	1.190744099	2.863242149	F7FLF2;P47198
Protein Metabolism	Rpl30	ribosomal protein L30	23.06979	22.74478	23.05549	27.76636	26.78044	27.69252	27.21308	27.62115	27.40576	26.84912	27.07511	27.47011	+	+	+	1.385026611	3.167693138	1.335746745	3.04091835	P62890
Protein Metabolism	Rpl31	ribosomal protein L31 pseudogene 49; r	22.79417	22.84879	21.95559	22.66933	22.35829	26.9997	26.20249	27.16192	22.21726	26.08008	25.98577	26.87537	+	+	+	1.458632117	3.113630295	1.367151355	2.722646713	ABZ9;D3ZZ60;D3ZU04;D4AC
Protein Metabolism	Rpl38	ribosomal protein L38	22.40356	22.35084	23.09879	21.70809	27.36403	28.97678	26.7986	27.90684	27.96168	26.4271	25.79352	26.56886	+	+	+	4.205316247	5.371243	3.687713822	4.297472477	ZW57
Protein Metabolism	Rps13	ribosomal protein S13 pseudogene 8; ril	21.80529	21.77198	22.79336	22.74901	25.25609	26.56883	25.77334	26.98978	25.85702	26.01935	25.88208	26.83533	+	+	+	3.698175602	3.867100239	4.371807378	3.868536472	0R6B8
Protein Metabolism	Rps15a	ribosomal protein S15a pseudogene 17;	21.92759	22.91949	27.88472	22.88749	27.60308	28.1455	27.36678	28.36761	27.89423	27.85501	27.23361	27.58949	+	+	+	1.564745637	3.965918064	1.482722366	3.738265514	S61;D3ZQT6
Protein Metabolism	Rps16	ribosomal protein S16 pseudogene 1; ril	22.90066	24.89684	22.71282	22.02395	27.07398	26.96906	27.22922	27.03926	27.81641	26.60229	26.6971	27.17455	+	+	+	3.151271104	3.944310665	2.946402856	3.939021111	P62250;M0RCH1
Protein Metabolism	Rps18	ribosomal protein S18 pseudogene 12; r	22.05285	21.98337	25.90428	22.66047	27.06435	26.47364	26.48034	26.65485	25.70248	26.10506	25.78234	26.49535	+	+	+	2.016467422	3.518053055	1.635889155	2.871062756	3ZII2;D3ZAU6
Protein Metabolism	Rps19	ribosomal protein S19 pseudogene 3; ril	22.14504	23.04451	27.43197	23.19117	27.10029	26.74124	27.42606	27.19563	27.64623	27.1039	26.68338	27.26919	+	+	+	1.423088115	3.162632942	1.44151896	3.222498894	LYF3;D3ZGN8;F1M395
Protein Metabolism	Rps27a	ribosomal protein S27a pseudogene 12;	23.07955	30.44904	22.52164	21.98437	32.70782	32.52465	32.6994	32.64689	32.85857	32.72513	32.78302	34.4621	+	+	+	2.187917796	8.136041641	2.279568806	8.6985569	G51;G3V9Z2;P62986
Protein Metabolism	Tars	threonyl-tRNA synthetase	23.79862	22.9739	22.39714	22.76786	26.1795	25.94985	26.13055	25.82338	26.57128	26.1172	26.44906	26.00232	+	+	+	4.204414618	3.036437988	4.271281275	3.30058527	Q5XHY5
Protein Metabolism	Tpp2	tripeptidyl peptidase II	22.66828	22.03465	21.41051	21.58435	27.38722	23.7416	25.78214	26.69962	27.41981	21.71176	27.47119	27.63069	+	+	+	2.49397284	3.978198528	1.506257235	4.133916855	Q64560
PSD	Dlg1	discs, large homolog 1 (Drosophila)	23.69722	22.00066	22.55049	22.84813	28.01175	27.64327	28.52073	28.70988	27.8776	27.93134	28.58474	28.7758	+	+	+	4.832569931	5.447283745	4.914659914	5.518247128	F1LNM0;Q62696
PSD	Dlg2	discs, large homolog 2 (Drosophila)	23.58284	22.20093	23.39127	23.12528	28.7314	27.7649	29.24173	28.88142	29.07383	28.23389	29.1308	29.14652	+	+	+	4.83424869	5.579784393	5.325978481	5.821178913	4;Q63622-7;Q63622-
PSD	Dlg3	discs, large homolog 3 (Drosophila)	22.3412	22.16951	22.04787	21.8136	28.1716	27.1062	28.31972	28.05683	28.16005	27.32817	27.96906	28.41788	+	+	+	5.950188608	5.820538044	6.332600257	5.875745773	Q62936;Q62936-2
PSD	Dlg4	discs, large homolog 4 (Drosophila)	22.91287	22.87671	23.21668	21.36459	30.42922	30.08182	30.53018	30.30084	30.38597	30.30617	30.68043	30.25372	+	+	+	5.739168104	7.74280405	5.764575225	7.813862801	P31016
PSD	Gphn	gephyrin	20.85885	22.68453	23.29967	22.43596	28.31894	27.66939	27.97279	28.03926	28.79086	27.886	28.44059	28.11506	+	+	+	4.378878676	5.680345058	4.422488527	5.988376141	3;Q03555;Q03555-4;Q03555-
PSD	Lrrc7	leucine rich repeat containing 7	23.27188	23.17783	22.81277	24.07106	27.54385	25.54943	27.39713	26.04623	27.64616	27.41418	28.38718	27.18527	+	+	+	2.971648728	3.300775051	4.614796216	4.32481432	4;F1LS33;F1M4K6;P70587-
PSD	Shank1	SH3 and multiple ankyrin repeat domair	22.18939	22.06152	21.79777	22.84534	27.73821	27.78432	28.10595	28.04526	28.23995	27.46287	28.30226	28.04911	+	+	+	6.4235937	5.694930077	5.957668151	5.790042877	4;Q9WV48-3;Q9WV48-
PSD	Shank2	SH3 and multiple ankyrin repeat domair	22.61671	22.85933	22.89363	22.10476	27.30539	23.41448	26.91011	25.5171	28.03643	26.51202	26.91707	26.61541	+	+	+	1.90769283	3.168165684	4.512786436	4.401625633	7;M0RDX8;M0R8T7;Q9QX74
PSD	Shank3	SH3 and multiple ankyrin repeat domair	23.47883	21.3054	22.37117	22.92776	27.71201	26.72871	28.10559	27.18603	28.22913	28.04573	27.96185	27.89799	+	+	+	3.945484694	4.912294865	4.637840544	5.512880802	Q9JLU4;Q9JLU4-2;Q9JLU4-3
Receptor associated	Dpp6	dipeptidyl-peptidase 6	24.048	23.18605	21.36225	22.53596	30.40213	29.76618	30.4182	30.1919	30.04257	29.73883	30.23558	30.59886	+	+	+	4.82533898	7.411536694	4.777428222	7.370892525	P46101-2;F1LMR7;P46101
Receptor associated	Frrs1l	ferric-chelate reductase 1-like	22.03076	22.53783	22.47049	22.43262	27.64547	27.40186	26.55834	26.9283	27.13663	27.55128	26.86183	27.03349	+	+	+	4.148437918	3.841525078	4.023873608	3.855578423	D3ZE85
Receptor associated	Gabarapl2	GABA(A) receptor-associated protein-lik	22.43014	22.92048	22.50783	22.83931	27.26589	27.44782	27.52305	27.18054	27.62457	26.78307	27.68291	27.76932	+	+	+	5.688432774	4.765565872	6.639831815	4.777880192	P60522;F1M1J6
Receptor associated	Gpd1l	glycerol-3-phosphate dehydrogenase 1-	21.30857	21.75387	23.94121	22.53039	27.20648	27.85097	28.31635	28.30921	28.23781	28.09944	29.07857	28.30461	+	+	+	7.240306618	4.679886341	5.791789396	4.790528297	D3ZAP9
Receptor associated	Opcml	opioid binding protein/cell adhesion mc	21.86122	22.90514	22.10218	22.21232	25.63533	25.78207	25.37981	25.17261	25.54734	25.9115	25.28683	25.32601	+	+	+	3.90459267	5.537242889	4.183337536	6.046595573	P32736;P32736-2;F1M2I5
Receptor associated	Prrt1	proline-rich transmembrane protein 1	22.59256	22.46683	23.22462	22.73817	28.92603	27.13036	26.40656	27.1034	28.95127	27.09848	27.07429	28.25296	+	+	+	4.757967272	3.222239494	4.737476947	3.247706413	Q6MG82
Receptor associated	Reep5	receptor accessory protein 5	28.22499	29.73088	29.92181	29.65911	31.34652	31.76679	31.8732	31.84315	31.55243	31.94454	31.82688	31.82084	+	+	+	3.760421217	4.636039257	4.334986162	5.088702679	B2RZ37
Receptor associated	Vdac1	voltage-dependent anion channel 1; sim	22.89959	21.90074	22.67273	21.96321	28.4213	29.17124	29.03467	29.20172	28.63551	29.64387	29.27553	29.21255	+	+	+	2.891262483	2.3232131	3.022833184	2.401972294	Q9Z2L0;D3Z9A9
Scaffolding	Ank1	ankyrin 1, erythrocytic	21.78049	25.71557	26.34518	21.25887	29.78085	28.7107	29.10784	29.34058	29.57824	29.28279	29.076	29.44053	+	+	+	4.296754717	4.766386986	3.91129129	4.696614742	D3Z9Z0
Scaffolding	Ank2	ankyrin 2, neuronal	22.78079	22.13284	21.793	22.52472	29.32577	28.86334	29.90297	29.38433	28.77596	29.05974	30.2589	29.94126	+	+	+	4.398680139	9.097883224	4.541617521	9.514266014	F1LM42;F1M5N3
Scaffolding	Ank2	ankyrin 3, node of Ranvier (ankyrin G)	21.40442	25.67992	25.82182	21.49043	30.77415	29.64506	30.349	30.20046	30.64513	29.82173	30.77313	30.75119	+	+	+	6.910021343	6.957888603	6.888730158	7.254119873	F1M9N9
Scaffolding	Ank3	ankyrin repeat and sterile alpha motif d	21.84521	22.3942	21.48018	22.97464	28.05222	22.51323	27.35018	26.7166	28.07561	27.51345	27.7458	28.13188	+	+	+	6.044428147	4.516335964	5.926863376	4.74802351	F1LNM3;F1LPH6
Scaffolding	Anks1b	annexin A3	22.77913	23.28992	26.21091	21.90339	28.21581	28.61017	29.35182	28.40665	28.61031	27.50028	29.88537	29.3055	+	+	+	4.079852088	3.976111889	2.252137336	3.147535324	3;D3ZY29;P0C6S7-2
Scaffolding	Epb4.1l2	erythrocyte membrane protein band 4.:	22.48624	23.02569	21.69448	22.31406	26.10364	27.29754	27.28604	27.89879	26.22227	27.26562	26.52618	28.29285	+	+	+	4.109291523	5.097229004	4.284972711	5.416779518	D3ZM69;D3ZDT1
Scaffolding	Epb41l1	erythrocyte membrane protein band 4.:	22.54637	22.12712	22.6624	25.72312	32.8735	31.90792	32.46777	32.20137	33.08797	32.38839	32.95845	32.68127	+	+	+	6.920690165	7.071296692	6.740876283	7.006712437	2;D4A9L5;Q9WTP0;D4ACZ4
Scaffolding	Epb41l3	erythrocyte membrane protein band 4.:	21.92987	22.42402	22.79582	23.02584	29.53803	29.36618	29.51657	29.58633	29.88609	29.62631	30.01492	29.66472	+	+	+	6.415935287	7.352870941	6.80071185	7.788641453	Q9JMB3;A3E0T0
Scaffolding	Fermt2	fermitin family homolog 2 (Drosophila)	22.75573	22.78536	23.68449	23.11906	27.57355	27.47522	27.6675	27.69372	27.52559	28.00935	27.8036	27.9982	+	+	+	3.99099589	3.55382967	3.049670347	3.404898167	Q5XI19
Scaffolding	Sept11	septin 11	22.73362	21.09167	21.96731	21.56844	26.41467	26.00731	25.4036	25.4399	25.3927	23.03431	25.58064	25.94353	+	+	+	2.729425448	5.100275517	2.591974707	5.279526711	B3GNI6-2;B3GNI6;B3GNI6-3
Scaffolding	Sept2	septin 2	23.39856	22.5398	24.69748	24.14103	29.38517	28.15793	29.00764	28.61506	29.48026	28.37414	29.38035	29.20925	+	+	+	6.150820524	6.598163605	6.11115021	6.83279562	Q91Y81
Scaffolding	Sept3	septin 3	21.36039	23.04906	22.89383	22.5372	26.20946	26.63478	26.22514	26.1372	25.81963	26.50823	26.32375	26.61119	+	+	+	2.196734801	5.459961414	2.258745191	5.569360733	2;D3ZPP8;Q9WU34-3
Scaffolding	Sept6	septin 6	22.51561	22.19498	22.89322	22.26786	30.03276	29.14892	29.35972	29.61546	29.9531	28.95227	29.6083	29.38484	+	+	+	6.378980456	7.061265945	5.63672625	7.201128483	B5DFG5
Scaffolding	Sept7	septin 7	22.94846	22.07049	22.52317	21.70745	30.12349	29.57093	29.5551	29.41153	30.36672	29.95116	30.05982	30.02644	+	+	+	2.718278721	6.643019199	2.803657025	6.898644447	F1LMC7;D4A0F5;Q9WVC0
Scaffolding	Sept9	septin 9	22.18862	22.08846	22.77869	23.76966	26.23849	26.43067	26.075	26.2966	26.7265	25.89386	25.0578	26.76686	+	+	+	1.672780334	3.984500408	5.410900853	5.693127632	3;Q9QZR6-4
Scaffolding	Sptan1	spectrin, alpha, non-erythrocytic 1 (alph	28.94815	31.01019	31.23968	31.971	35.04783	34.52776	35.16896	35.00135	34.8467	34.88385	34.96954	35.20325	+	+	+	3.109271263	4.144221783	3.166804147	4.183581829	P16086;E9PSZ3
Scaffolding	Sptb	spectrin, beta, erythrocytic	22.79827	22.1434	21.14485	21.90029	24.43267	26.85543	25.18801	25.2656	22.4344	25.8375	25.40798	26.15019	+	+	+	2.863283025	3.438727856	1.739793012	2.960817337	Q6XDA0
Scaffolding	Sptbn1	spectrin, beta, non-erythrocytic 1	22.75027	28.76126	29.2754	29.94531	34.28002	34.04751	34.26347	34.14034	34.06888	34.04066	34.24296	34.41122	+	+	+	2.102094604	6.499773979	2.102980821	6.507873535	G3V6S0
Scaffolding	Sptbn2	spectrin, beta, non-erythrocytic 2	23.32355	29.61449	29.2891	30.02762	31.79126	33.26403	32.71108	32.91214	32.50247	33.71426	32.92176	33.26136	+	+	+	1.532927957	4.60593462	1.693177389	5.036272049	F1MA36;Q9QWN8
Signaling	Abhd12	abhydrolase domain containing 12	20.97361	22.47096	21.87063	21.76278	26.60116	26.99118	26.50883	25.65335	27.09315	26.58334	26.43563	25.58543	+	+	+	4.517176938	4.669138432	4.382894826	4.654895782	Q6AYT7
Signaling	Abhd6	abhydrolase domain containing 6	22.84059	22.0576	21.71294	22.07221	25.97044	26.58806	26.39149	25.65905	26.17988	26.24958	26.24273	25.5854	+	+	+	4.80883178	3.981429577	5.000493782	3.89356184	Q5XI64
Signaling	Adcy5	adenylate cyclase 5	22.32356	23.71407	22.47691	22.89526	27.56449	22.09709	27.09467	26.66546	26.98685	26.37799	27.83074	27.71895	+	+	+	1.215751741	3.002975941	4.108749348	4.376181602	Q04400;G3V9G1
Signaling	Agpat1	1-acylglycerol-3-phosphate O-acyltransf	21.66084	23.41631	22.79801	24.15143	25.298	26.573	26.04009	26.25068	26.00118	25.48962	26.3299	26.37753	+	+	+	2.662179016	3.033797264	2.771728181	3.042914391	Q6MG85
Signaling	Agpat3	1-acylglycerol-3-phosphate O-acyltransf	21.66701	22.70695	22.01847	21.98824	26.12297	26.77756	27.04833	26.82287	26.88052	25.94773	26.49367	26.99636	+	+	+	5.347782801	4.597765923	5.066912153	4.484405994	G3V648

Supplementary Table 2. List of proteins significantly enriched in both immunoisolated FEZ1 and Kinesin-1 containing vesicles by quantitative mass spectrometry

Group	Gene Symbol	Gene Name	log LFQ intensity IP1 Control	log LFQ intensity IP2 Control	log LFQ intensity IP3 Control	log LFQ intensity IP4 Control	log LFQ intensity IP1 Kinesin-1	log LFQ intensity IP2 Kinesin-1	log LFQ intensity IP3 Kinesin-1	log LFQ intensity IP4 Kinesin-1	log LFQ intensity IP1 FEZ1	log LFQ intensity IP2 FEZ1	log LFQ intensity IP3 FEZ1	log LFQ intensity IP4 FEZ1	t-test Significant Kinesin-1 IP	t-test Significant FEZ1 IP	t-test Significant Kinesin-1 and FEZ1 IP	-Log t-test p value Kinesin-1 IP	t-test Difference Kinesin-1 IP	-Log t-test p value FEZ1 IP	t-test Difference FEZ1 IP	Majority UniProt protein IDs
Signaling	Anxa3	annexin A4	22.4219	22.56724	22.58748	23.34464	26.23963	24.73671	25.47339	25.47757	25.81849	25.37882	25.71479	25.82131	+	+	+	3.511952153	2.751510143	4.831812574	2.953035831	F1M0L7;P14669
Signaling	Anxa4	annexin A5	22.49834	23.01268	21.39674	21.95179	26.75964	26.8825	27.02369	26.9954	27.42742	27.93731	27.41296	27.44403	+	+	+	4.951815017	4.70042038	5.153575108	5.340543747	Q5U362;P55260
Signaling	Anxa5	annexin A6	21.95394	21.70636	21.08475	21.79934	28.48508	28.43639	28.90595	28.89646	28.85329	28.77502	28.53393	28.78285	+	+	+	7.100906389	7.044872284	7.437903685	7.100174427	P14668;Q66HH8
Signaling	Anxa6	adaptor-related protein complex 1, mu	22.63508	22.13601	21.12742	19.83815	29.13761	27.94687	29.36374	29.00705	29.84232	28.51627	29.88167	29.78183	+	+	+	4.410299695	7.42964983	4.590852792	8.071356773	P48037
Signaling	Appl1	adaptor protein, phosphotyrosine intera	22.69776	21.43704	22.1118	22.5579	26.3786	28.72936	27.70721	27.15383	27.71208	27.89718	26.94369	28.11103	+	+	+	4.054070125	5.291127205	5.144026822	5.464869499	D3ZWA8
Signaling	Bola2	bolA homolog 2 (E. coli); bolA homolog	22.63394	22.20405	21.88246	23.41419	25.67379	25.61922	26.95038	27.11178	25.90901	25.42589	27.05414	27.87853	+	+	+	3.47635922	3.805131435	3.106086827	4.033232212	D3ZVA6;D4A9P7
Signaling	Calb1	calbindin 1, 28kDa	23.08206	21.7207	23.09924	21.02841	29.43494	29.66579	28.7255	29.47468	28.18858	29.4818	29.10744	29.5753	+	+	+	4.851440242	7.092622757	4.550170511	6.855677605	P07171
Signaling	Calb2	calbindin 2	24.31811	21.50191	23.08392	22.57517	28.86535	28.43996	28.46716	28.99324	27.97662	27.97667	28.81409	29.4241	+	+	+	4.157916858	5.821644783	3.785525637	5.678091049	P47728
Signaling	Calm1	calmodulin 3 (phosphorylase kinase, del	22.69933	22.04354	22.07043	21.63913	31.2589	29.37379	26.752	21.71724	29.6489	29.04474	28.52835	27.91963	+	+	+	1.32028812	5.162371159	5.352434529	6.672296047	P62161;D4ABV5;Q5U206
Signaling	Ccny	cyclin Y	22.14034	21.57562	21.60636	21.3615	26.15364	26.87467	26.7585	26.60851	26.47299	27.07398	26.51351	26.50379	+	+	+	6.182480766	4.927875996	6.309185597	4.970113754	F1MA89
Signaling	Cd200	CD200 molecule	22.15325	23.64065	22.6682	24.38628	28.87593	27.83129	27.64141	27.49602	28.25827	27.35279	27.69942	27.58391	+	+	+	3.713728114	4.749069214	3.823152988	4.511505604	A0A5D0
Signaling	Cd47	CD47 molecule	22.4364	21.48535	23.19343	23.42686	29.31997	28.36394	28.86115	28.49167	29.4977	28.96719	28.89157	29.2865	+	+	+	4.807130042	6.123677254	5.115185933	6.525232315	P97829-2;P97829
Signaling	Cd59	CD59 molecule, complement regulatory	24.18129	22.54316	23.17972	22.26721	28.57719	27.77134	28.40844	28.33095	29.23635	27.73783	28.67094	28.87815	+	+	+	4.560062587	5.229134083	4.360376443	5.587973595	P27274
Signaling	Cops7a	COP9 constitutive photomorphogenic h	22.179	21.8796	21.44938	22.45524	25.43189	26.94458	26.02274	22.05677	25.38453	24.0954	26.09124	25.79395	+	+	+	1.547964204	3.12319231	3.319291616	3.35047245	F1MAA2;G3V8Z9
Signaling	Crk	v-crk sarcoma virus CT10 oncogene hom	21.54677	22.62158	23.95868	23.0094	25.89655	25.29632	24.559	25.20086	26.28623	24.89911	26.05362	26.32756	+	+	+	2.298625118	2.454072475	2.680713941	3.107520103	Q63768;Q63768-2
Signaling	Dgkb	diacylglycerol kinase, beta 90kDa	22.15193	22.74731	22.78416	24.20282	27.31164	28.0203	27.22748	27.17912	27.84571	27.45364	27.2379	27.22849	+	+	+	4.068895088	4.463079929	4.173839454	4.469881535	F1LP01;P49621
Signaling	Efhd2	EF-hand domain family, member D2	22.54198	22.6084	21.2733	23.93376	29.00766	28.74011	28.51642	28.4536	28.66207	28.62791	29.05613	28.84353	+	+	+	4.456479357	6.090087891	4.529082353	6.208049774	Q4FZY0
Signaling	Eps15l1	epidermal growth factor receptor pathv	22.57241	22.59727	26.94994	26.527	27.38944	27.19592	28.02797	27.91957	27.70655	27.51653	27.70471	27.45678	+	+	+	1.295629631	2.971569061	1.295484657	2.934486389	D3ZJR1
Signaling	Erlin2	ER lipid raft associated 2	23.07922	21.48343	22.06066	22.14454	26.49633	26.89169	26.14905	27.16949	26.75583	26.79501	26.69485	27.11535	+	+	+	4.529262144	4.484678745	4.996715378	4.648298264	B5DEH2
Signaling	Fkbp1a	FK506 binding protein 1A, 12kDa	27.52073	27.75977	28.46754	22.41849	31.45738	29.84002	31.4337	30.77368	31.10662	30.29187	30.28813	30.99024	+	+	+	1.625254332	4.334564686	1.58305321	4.127579689	Q62658
Signaling	Gmfb	glia maturation factor, beta	22.71033	22.1596	23.64241	22.38868	28.86479	29.35739	30.32667	29.50078	28.02986	29.61915	30.23786	29.21912	+	+	+	5.297050743	6.787156105	4.592166217	6.55124712	Q63228;M0RDJ4
Signaling	Gpi	glucose phosphate isomerase	22.61206	23.65297	22.66273	22.90541	30.37596	30.15714	30.74895	30.58361	29.96761	30.4171	30.8273	30.52953	+	+	+	6.822223287	7.508123398	6.568266107	7.477089405	Q6P6V0
Signaling	Gpm6a	glycoprotein M6A	28.52903	27.86183	29.27504	28.24377	32.96803	30.85386	32.00467	31.99833	33.2259	31.20961	31.69876	31.52655	+	+	+	3.241762247	3.478804588	3.156004273	3.437786579	Q812E9;Q812E9-2
Signaling	Grb2	growth factor receptor-bound protein 2	22.85702	22.49618	27.30322	21.844	28.059	26.58434	27.58835	27.14608	28.29763	27.4086	27.49297	27.73202	+	+	+	1.562714313	3.719338417	1.763483486	4.107699394	P62994;P62994-2
Signaling	Hpcal4	hippocalcin like 4	27.17179	22.16383	26.67061	21.58712	29.43526	28.79984	28.91698	28.54944	29.13707	28.71869	29.04077	29.27482	+	+	+	1.656281791	4.527041435	1.708654718	4.644499779	P35332
Signaling	Impa1	inositol(myo)-1(or 4)-monophosphatase	22.98219	22.41786	21.95269	23.59615	28.28872	27.55516	28.34405	28.07541	28.17636	27.70233	28.63149	28.31816	+	+	+	4.967054051	5.328617096	4.993874121	5.469867229	F1M978;P97697
Signaling	Inpp5a	inositol polyphosphate-5-phosphatase,	22.69446	22.22436	23.79908	21.42589	27.22876	25.07659	25.91969	26.55988	26.78531	25.49528	26.09283	25.2557	+	+	+	2.785950731	3.660282612	2.859738902	3.371333122	D3ZZX1
Signaling	Itpr1	inositol 1,4,5-triphosphate receptor, typ	22.89049	22.64359	22.25125	22.828	32.24289	32.65346	32.68564	33.23635	31.73457	32.67833	32.74941	33.52473	+	+	+	7.805069639	10.05125618	6.614689602	10.01843262	P29994-4
Signaling	Lamtor1	late endosomal/lysosomal adaptor, MAI	23.90734	23.74511	22.43349	21.73199	26.38383	26.54336	26.72182	25.94806	25.33518	26.51146	26.39359	26.31564	+	+	+	3.114805076	3.444785595	2.777122306	3.184480667	Q6P791
Signaling	Lamtor3	late endosomal/lysosomal adaptor, MAI	22.78106	22.63648	22.33042	23.81195	27.62331	27.0102	28.17769	27.46123	27.44987	22.72801	27.97684	27.78176	+	+	+	4.616944281	4.678132057	1.489563543	3.594139099	Q5U204
Signaling	Lingo1	leucine rich repeat and Ig domain conta	22.04758	21.24263	23.59367	22.23068	28.12987	26.75353	27.5947	27.43825	27.88605	27.02485	27.70313	27.83032	+	+	+	4.035098572	5.200448513	4.265847261	5.332448959	G3V881
Signaling	Lrrc8a	leucine rich repeat containing 8 family,	22.72265	23.70857	23.33574	22.39295	27.19882	24.74196	26.66221	26.35355	26.90816	25.10877	26.68552	27.18999	+	+	+	2.728504889	3.199157715	3.095656325	3.433132648	Q4V8I7
Signaling	Mif	macrophage migration inhibitory factor	23.94816	22.276	22.40742	21.60743	29.91271	28.52914	30.4071	30.09698	30.57876	29.60385	30.37181	31.47281	+	+	+	4.499275299	7.176729679	4.832119927	7.947058201	P30904;D3ZE63;D4A3P7
Signaling	Ncald	neurocalcin delta	27.4235	23.5046	23.32316	22.60139	28.90913	28.61136	29.08228	28.63934	28.97465	28.4985	28.72965	28.45403	+	+	+	2.247129664	4.597366333	2.180771446	4.451044083	Q5PQN0
Signaling	Ncs1	frequenin homolog (Drosophila)	23.36296	22.99542	22.96758	21.73018	28.17136	27.31709	27.6309	27.41377	28.09124	27.53564	27.92025	27.59356	+	+	+	4.702290062	4.869241714	4.934878343	5.021137238	P62168
Signaling	Ndrg2	NDRG family member 2	22.89039	27.20788	26.94648	21.12027	29.99375	30.18719	30.66297	31.42162	29.33637	30.22791	30.68937	31.1111	+	+	+	2.101331057	6.025127411	2.010046692	5.799936295	Q8VBU2-2
Signaling	Ndrg3	NDRG family member 3	23.36382	22.55351	21.09004	24.54715	27.83996	27.71181	27.88664	27.00721	27.64389	27.28489	28.06332	27.57015	+	+	+	3.113389777	4.722774982	3.160665333	4.751932621	Q6AYR2
Signaling	Ndrg4	NDRG family member 4	20.89392	23.55668	23.15907	21.41173	27.34198	26.46694	26.31392	25.47945	27.19254	25.93362	26.08902	26.2936	+	+	+	2.818703798	4.145224094	2.945030296	4.121843815	5;D3ZUT8;Q9Z2L9-3;Q9Z2L9-
Signaling	Nomo1	NODAL modulator 3; NODAL modulator	22.52177	22.16402	22.35601	22.19962	27.1575	26.54741	26.06223	26.13093	27.09235	25.97323	22.51627	26.42835	+	+	+	5.379446529	4.164161682	1.683525308	3.192193031	D3ZSA9
Signaling	Nudt3	nudix (nucleoside diphosphate linked m	21.96446	21.89217	22.4784	21.92513	26.95394	27.57109	27.40941	27.35195	26.61049	27.29088	27.28771	27.67707	+	+	+	6.821467124	5.256556511	5.955204908	5.151497841	Q566C7
Signaling	Numbl	numb homolog (Drosophila)-like	22.63453	22.00427	21.24561	22.30802	27.53312	27.15258	26.62087	26.40795	28.02406	27.17303	27.09989	26.94022	+	+	+	4.788659464	4.88051939	5.028940941	5.261191368	A1L1I3
Signaling	Palm	paralemmin	21.00758	23.77356	22.64439	21.93618	27.40625	26.25214	27.21354	26.51474	27.70774	26.00277	28.12406	27.20592	+	+	+	3.367357305	4.506244183	3.244228497	4.919697762	Q920Q0
Signaling	Pde10a	phosphodiesterase 10A	22.87409	23.3293	22.78141	23.08502	28.3997	28.15687	28.34143	28.31315	29.05997	27.88221	28.9173	28.2359	+	+	+	7.7819904	5.285333633	5.732467416	5.506389618	3;F1LX13;Q9QYJ6-2;Q9QYJ6-
Signaling	Pde2a	phosphodiesterase 2A, cGMP-stimulate	23.87849	22.09074	22.95422	22.20943	29.90009	29.70245	30.26528	29.69314	29.93635	29.97035	30.54305	30.17153	+	+	+	5.485520184	7.107021332	5.569060519	7.372102261	F8WFW5;Q01062
Signaling	Pde6d	phosphodiesterase 6D, cGMP-specific, r	22.27022	21.45241	22.3191	22.34351	24.8347	26.06562	26.09523	24.9933	25.11969	22.68636	25.74535	25.74819	+	+	+	3.833115188	3.400904655	1.941401326	2.728588104	D3ZRD3
Signaling	Pebp1	phosphatidylethanolamine binding prot	26.07278	26.47393	27.98022	29.05974	31.24241	31.13504	31.87826	32.20975	31.16429	31.28234	32.14404	31.9582	+	+	+	2.912252535	4.219696045	2.937811145	4.240547657	P31044
Signaling	Pef1	penta-EF-hand domain containing 1	22.46819	22.01511	22.97862	21.73479	25.04556	25.33603	25.54946	24.80651	25.56776	25.07463	25.94487	25.66631	+	+	+	4.00325651	2.885212898	4.231516424	3.264216423	Q641Z8
Signaling	Pi4ka	phosphatidylinositol 4-kinase, catalytic,	22.71854	22.59568	21.57627	22.9819	29.65106	28.43647	29.98834	29.98169	29.53304	29.2335	29.49192	30.27309	+	+	+	5.203647131	7.046289444	5.83878636	7.164787292	F1LRW9
Signaling	Pin1	peptidylprolyl cis/trans isomerase, NIM/	22.58149	21.41384	23.21483	22.83577	28.43209	29.14324	28.48512	28.81339	27.73731	29.07664	28.72511	28.71339	+	+	+	5.208212595	6.206977367	4.798965035	6.051628113	B0BNL2
Signaling	Pip5k1c	phosphatidylinositol-4-phosphate 5-kin	23.91573	22.0791	23.87069	22.66937	29.04051	28.51488	28.49476	28.37671	28.72667	28.68291	28.95358	28.70761	+	+	+	4.573265612	5.472991467	4.748563829	5.633966446	F1M8H6;Q5I6B8
Signaling	Pitpna	phosphatidylinositol transfer protein, m	22.25357	23.15527	23.06614	22.51322	29.1575	28.88381	29.52964	29.61959	28.94682	29.40562	29.87581	29.93719	+	+	+	6.433707787	6.550586224	6.170679604	6.794310093	P16446
Signaling	Plcb1	phospholipase C, delta 1	23.16936	22.32473	22.85427	23.8435	29.58282	29.40107	29.74437	29.6298	29.91228	29.70754	30.11855	29.85121	+	+	+	6.008352366	6.541550636	6.100476528	6.849429131	P10687;F1M084
Signaling	Plcd1	phospholipase C, delta 1	23.78336	21.64942	22.03391	23.08041	25.73707	25.88332	26.8776	25.82893	26.7024	26.11726	26.93326	26.00699	+	+	+	3.090554227	3.444953442	3.403068032	3.803201199	G3V9D1
Signaling	Plxna2	plexin A2	22.22885	20.96295	22.99552	23.02207	27.29237	23.52984	26.97399	26.64499	27.16317	24.58228	25.78077	27.09497	+	+	+	2.060603828	3.807952404	2.582680547	3.852949619	D3ZWP6
Signaling	Plxna4a	plexin A4	22.92419	22.71818	23.97546	21.83212	29.83375	27.85412	29.73583	29.61326	29.8642	28.5556	29.4691	29.41496	+	+	+	4.219234056	6.396752357	4.784124138	6.463473797	D3ZES7
Signaling	Plxnb1	plexin B1	21.70324	23.67387	22.10203	22.30755	26.70147	24.58308	26.72195	26.31025	26.33564	25.52418	27.14957	26.23261	+	+	+	2.808100328	3.63251543	3.415965077	3.863824844	D3ZDX5
Signaling	Plxnc1	plexin C1	22.03669	22.17401	21.10474	22.14344	26.10013	21.76688	25.97088	24.85761	26.85412	24.11108	26.0898	25.58858	+	+	+	1.449356326	2.809154987	3.017638132	3.796175957	D4A7M0
Signaling	Prnp	prion protein	23.15357	22.35357	24.51696	22.51644	28.21373	26.44582	27.67007	27.01372	27.51126	26.63381	27.89712	27.23544	+	+	+	3.279185816	4.200697422	3.531658009	4.184272766	P13852
Signaling	S100b	S100 calcium binding protein B	22.79606	22.09556	21.01964	22.52174	22.06943	28.62621	28.46883	27.8878	21.8959	28.68532	28.58355	30.58585	+	+	+	1.547838401	4.654814243	1.473247484	5.329404354	P04631
Signaling	Sema7a	semaphorin 7A, GPI membrane anchor	23.32717	21.88065	23.61404	23.21471	27.0403	23.21408	26.2624	26.2875	26.43694	25.66696	26.21241	26.43184	+	+	+	1.556919051	2.69192934	3.524730848	3.177896023	D3ZQP6
Signaling	Sh3bgrl	SH3 domain binding glutamic acid-rich p	22.72977	21.09198	23.11346	22.80478	26.49062	26.23612	26.15975	26.6618	26.42739	26.13208	26.78157	26.61625	+	+	+	3.813153267	3.952069759	3.842483524	4.054321289	B5DFD8

Supplementary Table 2. List of proteins significantly enriched in both immunoisolated FEZ1 and Kinesin-1 containing vesicles by quantitative mass spectrometry

Group	Gene Symbol	Gene Name	log LFQ intensity IP1 Control	log LFQ intensity IP2 Control	log LFQ intensity IP3 Control	log LFQ intensity IP4 Control	log LFQ intensity IP1 Kinesin-1	log LFQ intensity IP2 Kinesin-1	log LFQ intensity IP3 Kinesin-1	log LFQ intensity IP4 Kinesin-1	log LFQ intensity IP1 FEZ1	log LFQ intensity IP2 FEZ1	log LFQ intensity IP3 FEZ1	log LFQ intensity IP4 FEZ1	t-test Significant Kinesin-1 IP	t-test Significant FEZ1 IP	t-test Significant Kinesin-1 and FEZ1 IP	-Log t-test p value Kinesin-1 IP	t-test Difference Kinesin-1 IP	-Log t-test p value FEZ1 IP	t-test Difference FEZ1 IP	Majority UniProt protein IDs
Signaling	Sri	sorcin	24.36861	24.68942	21.71844	23.14207	27.61639	26.90953	27.63326	27.35607	27.84104	27.20751	27.18584	27.73059	+	+	+	2.861035809	3.899175644	2.922681467	4.011608124	B0BNJ1
Signaling	Wdr47	WD repeat domain 47	22.58591	21.7643	27.28304	22.86796	27.40625	26.99723	27.24	27.27453	27.8626	26.98327	26.69152	27.43046	+	+	+	1.561159156	3.604198933	1.536469586	3.616660118	G3V9M3
Signaling	Ywhab	tyrosine 3-monooxygenase/tryptophan	27.77568	22.19239	22.93835	21.73384	30.98929	29.14919	30.38936	31.25378	31.4774	29.33865	30.4828	30.99665	+	+	+	2.438946004	6.785337925	2.482720172	6.913806915	P35213;P35213-2
Signaling	Ywhag	tyrosine 3-monooxygenase/tryptophan	29.76984	22.01902	28.96157	29.25232	30.84797	31.85849	31.93523	32.1464	31.97613	31.43017	31.97186	31.93315	+	+	+	1.189562736	4.196334362	1.244951842	4.327138901	P61983
Signaling	Ywhah	tyrosine 3-monooxygenase/tryptophan	29.62029	28.19878	29.09023	27.96119	31.5478	30.75262	31.35652	31.55604	32.04944	30.45278	31.19437	31.53918	+	+	+	3.017581434	2.585625648	2.637865298	2.591319084	P68511
Signaling	Ywhaz	tyrosine 3-monooxygenase/tryptophan	30.6294	29.9327	31.52794	30.64616	33.02193	33.0904	33.24232	33.25856	33.28816	32.95834	33.52484	33.17715	+	+	+	3.518031814	2.46925354	3.488695026	2.553071022	P63102
Synaptic Vesicle	Slc17a7	solute carrier family 17 (sodium-depenc	21.19142	22.41783	22.74751	21.76064	27.88192	27.95959	28.33513	28.67111	28.44051	28.55918	29.17655	28.95679	+	+	+	5.388200261	6.182586193	5.646681521	6.753905296	Q62634-2;Q62634
Synaptic Vesicle	Slc30a3	solute carrier family 30 (zinc transporter	22.57893	22.66903	23.64013	22.05569	27.50484	27.6147	27.42998	27.5235	27.36277	27.96769	28.34651	27.98826	+	+	+	5.150321843	4.78231287	4.960086348	5.180364609	Q6QIX3;Q6QIX3-2
Synaptic Vesicle	Sv2a	synaptic vesicle glycoprotein 2A	22.04635	23.12807	21.88472	25.52785	31.14795	30.42412	30.61675	30.30454	31.36979	30.36922	30.84797	30.66983	+	+	+	3.891048056	7.476599216	3.937274908	7.667458057	Q02563
Synaptic Vesicle	Sv2b	synaptic vesicle glycoprotein 2B; hypoth	23.52012	22.02815	23.67856	21.39716	30.12866	29.38819	30.86386	29.81762	30.2544	29.81998	30.24139	30.87438	+	+	+	4.592690612	7.393583298	4.837333606	7.641536713	Q63564;G3V7H7
Synaptic Vesicle	Syn1	synapsin I	22.63715	23.50286	21.86139	21.44431	26.65608	21.81416	27.3268	28.68906	26.74791	22.4912	29.49537	30.18813	+	+	+	1.275748347	3.76009655	1.448576135	4.869226456	P09951-2
Synaptic Vesicle	Syngr1	synaptogyrin 1	26.71004	21.79553	27.96703	23.13617	28.43849	26.76661	29.48481	29.52133	28.39636	29.72819	29.5496	29.68082	+	+	+	1.209211723	3.650619507	1.607302376	4.436551094	Q62876
Synaptic Vesicle	Syngr3	synaptogyrin 3	22.58972	21.23782	23.50508	22.52494	29.31903	28.53579	28.94391	28.38336	29.89083	29.35846	29.00059	28.27204	+	+	+	4.770041805	6.331132412	4.598398633	6.666092873	D4ABK1
Synaptic Vesicle	Syp	synaptophysin	27.5773	28.9194	29.65596	29.2252	31.67405	31.81505	31.64959	31.5483	31.18601	31.95102	31.27581	31.48697	+	+	+	3.111286378	2.827280521	2.811611053	2.630486012	P07825
Synaptic Vesicle	Syt1	synaptotagmin I	23.73525	22.74212	21.84689	24.97863	30.66238	30.80609	31.2974	30.23466	29.82493	30.8629	32.5898	32.69627	+	+	+	4.357511946	7.424414158	3.818950189	8.167757034	P21707
Synaptic Vesicle	Syt2	synaptotagmin II	22.99574	22.19459	23.35619	21.18389	22.63201	26.91593	27.25043	27.1417	22.9798	26.5466	28.44865	29.89649	+	+	+	1.57119309	3.552418232	1.555263125	4.535284519	G3V6M3;P29101
Synaptic Vesicle	Ttyh1	tweety homolog 1 (Drosophila)	21.40836	22.57292	22.97044	21.81705	28.47433	26.95116	27.76661	27.79953	27.53289	27.75888	28.72926	28.06795	+	+	+	4.644854286	5.555711746	4.945979793	5.830051422	P0C5X8
Synaptic Vesicle	Vamp2	vesicle-associated membrane protein 2	22.78446	28.78057	23.53771	23.10825	30.67993	31.24474	30.22986	29.95629	30.77902	31.43117	30.53176	29.93143	+	+	+	2.213150087	5.974957466	2.251932807	6.115595341	P63045
Synaptic Vesicle	Vat1	vesicle amine transport protein 1 homo	22.66166	22.95614	23.02418	22.50116	26.67559	23.59701	24.9574	25.81573	26.57687	23.21559	27.27026	26.56754	+	+	+	1.999755212	2.47564888	1.834003711	3.121780872	Q3MIE4
Trafficking	Chmp4b	chromatin modifying protein 4B	22.63347	22.94132	23.74276	22.13829	25.57285	26.43893	26.67882	27.00838	26.23079	26.96598	26.79811	27.26177	+	+	+	3.636260765	3.560787678	4.204902128	3.950201511	D4A9Z8
Trafficking	Chmp6	chromatin modifying protein 6	23.45577	21.58249	24.36846	22.20068	25.62737	25.59571	25.66397	25.55807	24.37551	25.69216	25.6967	25.28274	+	+	+	2.307767711	2.709428787	1.827956799	2.359927654	D3ZDR2
Trafficking	Cpne6	copine VI (neuronal)	22.04491	22.44523	22.50408	23.22995	26.29019	22.3692	26.46178	26.06052	26.21252	25.98096	27.14501	26.96763	+	+	+	1.456731761	2.73938036	4.405636871	4.020489693	D4ACG7
Trafficking	Cyfip2	cytoplasmic FMR1 interacting protein 2	22.26279	22.25336	23.01918	21.85596	28.57748	28.48696	29.1244	28.87449	28.53875	28.63038	29.25129	29.37136	+	+	+	6.31578556	6.41801405	6.052731296	6.600122452	D3ZX82
Trafficking	Exoc5	exocyst complex component 5	21.99008	22.55145	23.03999	22.74391	25.23476	25.82665	24.88958	24.96838	25.73345	25.94568	25.33081	26.32179	+	+	+	3.877962974	2.648484707	4.413361089	3.251573563	P97878
Trafficking	Exoc8	exocyst complex component 8	21.71909	22.62969	22.05359	21.75277	25.50209	21.96787	25.87799	26.37398	26.2642	23.0795	25.27615	26.49951	+	+	+	1.517736127	2.891699314	2.153484469	3.241054058	O54924
Trafficking	Fkbp2	FK506 binding protein 2, 13kDa	23.67922	22.72686	21.3371	22.7955	28.09068	29.12008	28.68278	29.31058	28.3244	29.36643	28.93238	28.90475	+	+	+	4.501664432	6.166359425	4.652279288	6.247319221	D3ZZR9
Trafficking	Flot1	flotillin 1	24.48169	20.97473	21.72081	23.57585	26.49867	26.70701	27.7465	27.8262	27.50218	22.57144	28.47456	27.73046	+	+	+	2.661177411	4.506328583	1.312780331	3.881390572	Q9Z1E1;Q9Z1E1-2
Trafficking	Htt	huntingtin	22.61226	23.07629	23.37586	22.50641	28.08269	29.69657	28.74021	25.3291	27.28489	29.00908	29.15448	29.05468	+	+	+	2.729852581	5.069437504	4.619142593	5.733078957	G3V9P7;P51111
Trafficking	Iqsec2	IQ motif and Sec7 domain 2	22.63586	22.02833	22.91118	20.58027	24.95389	25.04752	25.15391	24.16599	25.92585	25.7173	26.50714	25.44266	+	+	+	2.57824771	2.791419506	3.307650629	3.859329224	D4A4I4;D3ZXR8;D3ZV71
Trafficking	LOC68659	similar to IQ motif and Sec7 domain 1	23.01005	21.93703	22.38871	23.31095	26.44171	26.5809	25.67791	25.48392	28.32856	26.95527	27.30191	26.44054	+	+	+	3.755585527	3.384419441	4.008381722	4.594879627	M0R5C6;M0R4G7;M0RBD0
Trafficking	Psd3	pleckstrin and Sec7 domain containing 3	23.3331	22.54421	21.68779	26.99712	27.9624	26.31197	27.17836	26.3786	28.63111	26.94	27.16634	26.70293	+	+	+	1.445724459	3.317278862	1.610992121	3.719538212	D4A9C3;D3ZUW0
Trafficking	Scamp3	secretory carrier membrane protein 3	22.94946	22.87677	22.0945	22.48341	26.32589	26.62101	26.38517	26.74804	26.3837	27.06774	25.67533	26.23224	+	+	+	5.685337029	3.918994427	4.41577932	3.738718987	E9PTW1
Trafficking	Scamp5	secretory carrier membrane protein 5	22.2578	26.00299	22.76708	22.10773	26.57644	27.49564	28.22862	27.29325	28.67162	28.82122	28.24645	28.22518	+	+	+	2.248258298	4.11458683	2.860574872	5.20721674	F1M882
Trafficking	Sec24c	SEC24 family, member C (S. cerevisiae)	22.58771	23.07653	20.72333	22.26424	24.83292	26.46597	24.47054	23.56883	26.03588	25.72219	25.52286	26.03729	+	+	+	1.828435193	2.671613693	3.374332245	3.666602135	B5DEG8
Trafficking	SNAP25	synaptosomal-associated protein, 25kDa	27.0071	28.51032	29.48243	29.33909	32.49693	31.68921	31.91167	32.44801	32.08052	32.07565	32.463	32.435	+	+	+	2.977908685	3.551719666	3.151581749	3.678806782	P60881;F8WG75
Trafficking	Snap29	synaptosomal-associated protein, 29kDa	20.59954	20.28448	21.40951	21.90004	25.51851	25.86233	25.83101	25.88553	25.78432	26.21108	26.16676	26.27087	+	+	+	4.784735222	4.725954056	4.916447429	5.059862614	Q9JI56;Q9Z2P6
Trafficking	Snca	synuclein, alpha (non A4 component of	28.3699	28.29925	26.51355	28.92437	30.48328	30.32828	30.74807	30.407	31.30313	30.3741	31.41604	31.63634	+	+	+	2.450702978	2.464889526	2.747475376	3.15563345	P37377;P37377-2
Trafficking	Sncb	synuclein, beta	29.25228	23.02544	22.95288	22.31767	29.8106	28.63256	30.00785	29.81078	30.63712	29.16849	29.81472	30.3226	+	+	+	1.686629869	5.178379536	1.823267619	5.598660469	Q63754
Trafficking	Snx12	sorting nexin 12	22.12169	22.42111	23.92925	23.25595	26.90369	28.01079	26.86231	27.36052	26.60922	26.8527	25.67131	27.7585	+	+	+	3.948714346	4.352326393	3.159817701	3.790933132	D4A719
Trafficking	Stx12	syntaxin 12	29.81932	23.40923	23.24612	21.62751	28.34083	29.01025	27.29798	27.80999	27.87062	28.39219	28.10804	27.91228	+	+	+	1.000311537	3.589214802	1.006911456	3.545234203	G3V7P1
Trafficking	Stx1a	syntaxin 1A (brain)	20.4704	27.98615	28.13222	27.68459	31.3835	30.74399	31.25429	30.98033	31.24553	30.6327	31.37943	31.35621	+	+	+	1.437436058	5.022187233	1.454494196	5.085128307	P32851
Trafficking	Stx1b	syntaxin 1B	27.68043	29.97378	29.73433	29.48216	32.39599	31.89555	32.45758	32.81276	32.0782	31.98434	32.73235	32.8384	+	+	+	2.905182066	3.172794342	2.872892369	3.190648556	P61265
Trafficking	Stx6	syntaxin 6	22.01974	22.26684	21.79095	22.87074	27.46326	27.54193	27.74695	27.55545	27.30687	27.33509	27.43754	27.21039	+	+	+	6.266489937	5.339830875	6.173297584	5.085404873	Q63635
Trafficking	Stxbp1	syntaxin binding protein 1	24.36191	22.54022	22.39004	20.61331	31.36635	32.43862	32.85239	32.16156	31.95161	32.23829	33.17976	33.65128	+	+	+	4.640862055	9.728360176	4.674016084	10.27886438	P61765
Trafficking	Stxbp5	syntaxin binding protein 5 (tomosyn)	23.0406	22.33542	21.17322	22.11968	28.38747	28.72822	28.8918	28.29557	28.90483	28.71116	29.00849	28.403	+	+	+	5.361412086	6.408535957	5.448183262	6.589639664	2;Q9WU70-3
Trafficking	Stxbp5l	syntaxin binding protein 5-like	22.30471	21.74421	23.18892	22.55181	26.61667	26.81078	26.52667	24.9664	26.27565	26.8903	27.99361	27.38327	+	+	+	3.46326188	3.782716274	4.220763269	4.688295364	D3ZFH7;M0RD32
Trafficking	Tf	transferrin	22.36119	21.69787	23.08555	21.72259	28.61534	28.1866	28.23316	28.35452	28.40572	28.3753	28.70289	28.42702	+	+	+	5.716050144	6.130602837	5.808908777	6.260935307	Q7TMC7
Trafficking	Tfrc	transferrin receptor (p90, CD71)	21.80526	21.71025	23.01004	22.53521	27.82887	25.47612	26.12957	25.0112	26.97158	25.36803	24.3656	25.23418	+	+	+	2.849929685	3.84624958	2.675415758	3.219657898	G3V679;Q99376
Trafficking	Trappc6b	trafficking protein particle complex 6B	23.80172	23.44978	22.52097	23.48715	25.80164	25.85664	26.23153	26.10167	25.66998	25.77533	26.21945	26.15217	+	+	+	4.00958757	2.682967663	3.858901136	2.63932991	D3ZES2
Trafficking	Vamp4	vesicle-associated membrane protein 4	21.8501	22.61585	22.43945	22.51295	26.35318	27.21391	26.00697	22.90223	26.57349	26.51608	26.24627	25.9329	+	+	+	1.846462048	3.264486313	5.655768892	3.962596416	D4A560
Trafficking	Vamp7	vesicle-associated membrane protein 7	23.00791	23.67469	21.98933	22.23398	26.32567	26.47758	27.02411	26.75863	26.25784	26.59676	25.9759	26.8208	+	+	+	4.105847841	3.920022488	3.880791693	3.686347485	Q9JHW5
Trafficking	Vapa	VAMP (vesicle-associated membrane pr	23.26682	23.52642	27.31984	22.59507	29.76055	29.98712	29.28505	29.02999	29.49488	29.94182	29.03795	28.75649	+	+	+	2.570032613	5.338637829	2.467391395	5.130745411	Q9Z270
Trafficking	Vapb	VAMP (vesicle-associated membrane pr	22.9937	23.12439	21.69226	22.06471	29.04734	29.8388	28.44719	28.19667	28.88207	29.07475	28.04437	28.56754	+	+	+	4.82997279	6.413733006	5.228380212	6.17341423	Q9Z269
Trafficking	Vps26b	vacuolar protein sorting 26 homolog B (	22.14813	26.24305	22.00944	22.2395	27.69525	26.11126	27.50839	27.50726	27.22647	26.89897	27.12396	26.89989	+	+	+	1.997876511	4.045509338	2.025348026	3.877290726	B1WBS4
Trafficking	Vps29	vacuolar protein sorting 29 homolog (S.	22.50695	21.38213	22.03089	22.55101	27.48416	27.76553	28.33905	28.14044	27.95621	28.15214	27.54104	28.40925	+	+	+	5.650550455	5.814551353	5.720976608	5.896915913	B2RZ78;B2RZ78-2
Trafficking	Vps35	hypothetical protein LOC100133770; va	20.3999	27.73738	27.27888	26.3394	29.60355	28.98272	30.01079	29.47686	29.53577	29.27206	30.18707	29.66326	+	+	+	1.258460484	4.079592705	1.310657216	4.225653172	G3V8A5
Transporter	Abca2	ATP-binding cassette, sub-family A (ABC	22.07724	22.86326	21.80538	23.88622	26.08032	24.91506	26.97356	25.88367	25.26974	24.3771	26.00241	25.6064	+	+	+	2.717121611	3.305130482	2.418731488	2.655889034	G3V7X4;Q9ESR9
Transporter	Atp6ap1	ATPase, H+ transporting, lysosomal acce	22.12463	22.02384	21.69403	21.54941	27.25737	25.76572	26.17436	25.75822	26.27213	25.42195	25.81975	25.94456	+	+	+	4.609186237	4.39094162	5.737910751	4.016620636	Q6IRF8;O54715
Transporter	Atp6v0a1	ATPase, H+ transporting, lysosomal V0 s	26.37385	28.27932	29.21065	27.41078	32.31498	31.94918	31.78121	31.85263	31.9162	31.98193	32.02848	32.01358	+	+	+	3.281693098	4.155847073	3.3297186	4.166398048	Q2I6B2;P25286;P25286-2
Transporter	Atp6v0c	ATPase, H+ transporting, lysosomal 16kI	23.33657	22.96757	22.67203	22.10328	31.27448	32.10036	28.89006	28.55563	29.43716	30.83138	29.34997	30.09735	+	+	+	3.734859598	7.435270309	5.514750782	7.15910244	P63081
Transporter	Atp6v0d1	ATPase, H+ transporting, lysosomal 38kI	26.22055	27.94866	29.12704	28.80079	30.92769	30.96664	31.46718	31.6513	31.35327	31.2282	31.39182	31.75633	+	+	+	2.514895752	3.228943348	2.679347871	3.408142567	Q5M7T6

Supplementary Table 2. List of proteins significantly enriched in both immunoisolated FEZ1 and Kinesin-1 containing vesicles by quantitative mass spectrometry

Group	Gene Symbol	Gene Name	log LFQ intensity IP1 Control	log LFQ intensity IP2 Control	log LFQ intensity IP3 Control	log LFQ intensity IP4 Control	log LFQ intensity IP1 Kinesin-1	log LFQ intensity IP2 Kinesin-1	log LFQ intensity IP3 Kinesin-1	log LFQ intensity IP4 Kinesin-1	log LFQ intensity IP1 FEZ1	log LFQ intensity IP2 FEZ1	log LFQ intensity IP3 FEZ1	log LFQ intensity IP4 FEZ1	t-test Significant Kinesin-1 IP	t-test Significant FEZ1 IP	t-test Significant Kinesin-1 and FEZ1 IP	-Log t-test p value Kinesin-1 IP	t-test Difference Kinesin-1 IP	-Log t-test p value FEZ1 IP	t-test Difference FEZ1 IP	Majority UniProt protein IDs
Transporter	Atp6v1a	ATPase, H+ transporting, lysosomal 70kI	21.98631	22.32776	22.2794	21.84817	29.3682	30.58898	30.94824	29.85055	29.85742	30.38299	32.04228	31.85159	+	+	+	6.187339142	8.078579903	5.449193684	8.923158169	D4A133;M0RBP6
Transporter	Atp6v1b2	ATPase, H+ transporting, lysosomal 56/!	21.87265	22.06553	29.95421	29.51198	30.3078	30.22458	30.42992	30.57028	30.43857	30.37886	31.45978	31.21697	+	+	+	1.045636571	4.532051086	1.167612154	5.022450924	P62815
Transporter	Atp6v1c1	ATPase, H+ transporting, lysosomal 42kI	21.84299	27.59783	28.32475	27.55961	30.11347	29.34731	29.30578	30.17224	29.58377	29.37385	29.54342	29.36801	+	+	+	1.173412218	3.403405666	1.082484828	3.135967731	Q5FVI6
Transporter	Atp6v1d	ATPase, H+ transporting, lysosomal 34kI	23.45404	23.74881	27.53326	22.81168	29.17624	29.87929	30.03787	29.4795	28.99502	29.64346	30.30802	30.09673	+	+	+	2.543385836	5.256280422	2.549379077	5.373859406	Q6P503
Transporter	Atp6v1f	ATPase, H+ transporting, lysosomal 14kI	22.3375	21.97578	22.55142	21.32869	29.85524	28.78222	30.13589	29.2269	29.92266	28.32444	30.04127	29.39147	+	+	+	5.771798344	7.451717377	5.347710767	7.371614933	P50408
Transporter	Atp6v1g2	ATPase, H+ transporting, lysosomal 13kI	22.66436	22.83337	23.35031	21.63894	28.82496	29.52324	29.35321	29.75269	28.67587	29.82074	28.93778	30.29516	+	+	+	5.495965145	6.741778851	4.908866194	6.810638428	Q8R2H0
Transporter	Atp6v1h	ATPase, H+ transporting, lysosomal 50/!	21.86677	26.50519	26.63395	22.78229	29.05256	28.52036	29.00185	28.98041	28.91065	28.08796	29.0777	29.24848	+	+	+	1.925916306	4.441745281	1.871913629	4.384149075	E9PTI1
Transporter	Slc12a2	solute carrier family 12 (sodium/potassi	23.04634	22.59754	22.81815	22.09804	27.0151	22.36971	27.05869	27.28701	27.40949	26.02268	26.8992	27.4299	+	+	+	1.465920232	3.292612076	4.500441268	4.300300598	E9PTX9
Transporter	Slc12a5	solute carrier family 12 (potassium-chlo	22.671	26.39306	25.16116	24.16867	31.45503	31.11092	31.56565	31.26344	31.53013	31.55627	31.58508	31.66793	+	+	+	3.839673917	6.750287533	3.941571057	6.986382008	2;F1LNP4;D3ZGI9
Transporter	Slc15a2	solute carrier family 15 (H+/peptide trar	22.11611	22.04746	22.78459	22.63948	25.64661	23.47514	25.26184	25.08982	25.74253	23.65613	25.17478	25.36893	+	+	+	2.529683482	2.471443653	2.710855486	2.588681221	Q63424
Transporter	Slc1a2	solute carrier family 1 (glial high affinity	22.45534	22.23086	25.15742	22.59367	32.37503	32.1624	32.01976	31.94977	32.18562	32.52818	32.96272	32.80453	+	+	+	4.895916622	9.017416954	4.97852006	9.510940552	P31596;P31596-2
Transporter	Slc1a3	solute carrier family 1 (glial high affinity	22.66743	22.58372	23.51454	21.8693	31.05866	30.4343	31.74855	31.71428	30.98142	30.10011	30.34011	30.73699	+	+	+	5.82085758	8.580199718	6.018130471	7.880908966	G3V846;P24942;P24942-2
Transporter	Slc25a22	solute carrier family 25 (mitochondrial c	22.83856	23.59867	22.03679	22.15436	28.02986	26.97356	27.72006	27.2238	27.51443	27.36961	27.97552	27.91428	+	+	+	4.515124365	4.829724312	4.879359416	5.036364555	Q5FVG4
Transporter	Slc25a3	solute carrier family 25 (mitochondrial c	22.35043	22.2675	27.03118	23.40743	29.81595	29.64916	30.0321	29.91471	29.87357	29.76213	30.32217	30.10462	+	+	+	2.788996233	6.088842869	2.839768279	6.251486778	G3V741;P16036
Transporter	Slc25a4	solute carrier family 25 (mitochondrial c	21.65817	22.57318	21.20129	23.36839	29.96086	29.43139	30.09874	30.16567	29.63234	30.07265	30.31527	30.23774	+	+	+	5.275681647	7.713906765	5.346590898	7.864245892	Q05962;Q6P9Y4
Transporter	Slc25a5	solute carrier family 25 (mitochondrial c	22.49442	22.90842	28.02126	27.68479	31.54917	31.03872	31.24496	31.17182	30.75693	31.32539	31.77996	31.21228	+	+	+	2.145531953	5.973950386	2.1370605	5.991418362	Q09073;D3ZE29
Transporter	Slc2a1	solute carrier family 2 (facilitated glucos	21.47279	23.07771	22.34604	22.65396	26.73428	25.92212	26.47164	26.52755	26.29845	26.30656	25.67347	26.44982	+	+	+	4.37096675	4.026269913	4.224713632	3.794446945	P11167
Transporter	Slc2a3	solute carrier family 2 (facilitated glucos	20.75624	22.54226	22.03317	22.43878	28.61295	22.38665	28.14131	27.82189	28.0414	23.0499	27.71633	28.3449	+	+	+	1.710114094	4.798089981	1.983743494	4.84552002	Q07647
Transporter	Slc3a2	solute carrier family 3 (activators of dib	22.78082	20.5784	23.44459	23.02847	31.33191	31.19701	31.1471	31.30438	31.18341	31.37482	31.60638	31.60523	+	+	+	5.021197875	8.787031651	5.051684638	8.984390259	Q794F9
Transporter	Slc4a10	solute carrier family 4, sodium bicarbon	22.41408	22.02013	22.72889	23.29438	27.78407	27.5984	28.22085	28.49156	27.88786	28.1162	28.37223	28.85988	+	+	+	5.428166911	5.409348011	5.535487287	5.694671154	4;Q80ZA5;Q80ZA5-
Transporter	Slc4a4	solute carrier family 4, sodium bicarbon	21.86882	22.68953	23.62134	21.49975	29.77776	29.83008	30.03774	30.40019	30.4178	30.15967	30.45071	30.94238	+	+	+	5.292477212	7.604082584	5.414933124	8.085278511	Q9JI66-3;Q9JI66;Q9JI66-2
Transporter	Slc7a11	solute carrier family 7, (cationic amino a	21.79716	23.41923	23.18543	22.12353	26.79736	25.7417	25.24779	26.11825	26.41107	26.54361	26.15864	26.02667	+	+	+	3.205772943	3.344937801	3.935562856	3.653660297	D4ADU2
Transporter	Slc7a5	solute carrier family 7 (cationic amino a	22.4269	22.82084	22.64595	22.63738	26.88786	26.24732	26.90793	26.21106	26.94369	26.76155	27.20508	26.93225	+	+	+	5.637778355	3.905774117	7.476858705	4.327873707	Q63016
Transporter	Slc8a1	solute carrier family 8 (sodium/calcium	22.6422	22.19745	23.70933	23.04673	28.70652	27.69039	28.43185	28.79844	28.43285	27.89047	28.82423	28.83219	+	+	+	4.992067244	5.507874489	5.142403266	5.596007824	4;Q01728-
Transporter	Slc8a2	solute carrier family 8 (sodium/calcium	22.25458	21.48706	23.05846	22.0253	30.83673	29.51339	30.32163	30.53742	30.54112	30.38977	30.54803	30.32914	+	+	+	5.825143816	8.095941067	6.560073415	8.245661259	P48768;F1M9A2
Transporter	Slc9a1	solute carrier family 9 (sodium/hydroge	22.39593	22.45131	23.22498	22.06103	26.05654	25.29614	25.67328	24.84685	26.29232	25.79409	25.87835	23.30757	+	+	+	3.756082315	2.934889317	2.075639309	2.784769058	P26431
Transporter	Slc9a3r1	solute carrier family 9 (sodium/hydroge	22.46207	22.40554	23.93025	22.08859	27.89481	23.05637	27.37617	26.98359	27.80821	26.50077	27.60414	28.11867	+	+	+	1.650601355	3.606125355	3.934623127	4.786335468	Q9JJ19
Tumor related	Bri3bp	BRI3 binding protein	22.46655	22.80536	22.73019	21.92235	26.43632	26.7888	26.25846	27.12416	26.2469	26.73092	26.12054	26.91969	+	+	+	5.264319912	4.17082119	5.18050219	4.023396492	Q5U3Z5
Tumor related	Bsg	basigin (Ok blood group)	22.96683	22.61711	22.74363	22.78043	29.22766	27.67405	28.7301	28.57467	28.78734	28.37691	28.77593	27.95482	+	+	+	5.635796903	5.774620056	6.775917974	5.696753025	P26453;P26453-2
Tumor related	Tpd52l2	tumor protein D52-like 2	21.60175	22.16211	21.98811	22.74172	25.56119	26.26664	25.18597	25.4964	25.85576	25.51069	25.01265	25.9802	+	+	+	4.393428085	3.504129887	4.427909707	3.466404915	Q6PCT3
Ubiquitin	Cacybp	similar to calcyclin binding protein; calcy	21.27588	20.88074	22.0792	22.16945	25.93193	26.91593	26.35505	26.42097	26.9345	26.57365	26.29941	26.4241	+	+	+	4.875235251	4.804651737	5.170215044	4.956596851	Q6AYK6
Ubiquitin	Cand1	cullin-associated and neddylation-dissoc	23.15205	28.18068	24.86155	25.78162	29.67497	31.08664	30.21651	30.25192	30.25135	30.45081	29.99159	30.85259	+	+	+	2.351616756	4.81353569	2.43226562	4.892612457	P97536
Ubiquitin	Cul1	cullin 1	21.8429	21.15693	21.48503	22.24241	26.24282	26.93618	25.19618	24.67017	27.11307	25.18143	26.47418	25.63262	+	+	+	3.463293245	4.079524517	3.981652918	4.418511391	B1WBY1
Ubiquitin	Cul3	cullin 3	21.7261	22.98757	23.16443	22.96176	26.8242	27.87367	26.49573	27.26651	27.07449	26.57469	27.14083	27.61217	+	+	+	4.209306077	4.405065536	4.511555295	4.390581608	B5DF89
Ubiquitin	Cul5	cullin 5	21.20781	22.04421	22.84241	22.41469	26.09211	27.2305	25.14767	25.90504	26.54494	26.04935	25.96792	26.50785	+	+	+	3.43091352	3.966553211	4.460798597	4.140235424	Q9JJ31
Ubiquitin	Fbxo2	F-box protein 2	23.27902	23.24006	22.71985	22.1676	26.62255	26.67976	27.49755	27.75334	27.63305	27.2006	27.5773	27.83159	+	+	+	4.488021134	4.28666687	5.43675239	4.709002972	G3V774
Ubiquitin	Fbxo41	F-box protein 41	22.08292	22.93386	23.24847	23.02018	27.18509	26.38133	26.86278	26.79971	27.70313	26.0984	27.52612	27.38739	+	+	+	4.917748881	3.985867977	4.18000544	4.357400417	D3ZT20
Ubiquitin	Huwe1	HECT, UBA and WWE domain containin	22.30177	23.03667	23.15429	22.80911	27.87256	29.07516	28.30583	27.73596	28.45148	28.09341	28.17688	28.75462	+	+	+	5.298471907	5.421912193	6.356728636	5.543635368	F1MAP9
Ubiquitin	Mycbp2	MYC binding protein 2	22.02562	21.25539	21.40835	23.18583	26.07849	23.6704	26.10737	23.9778	26.71109	23.69008	24.18684	24.33176	+	+	+	2.037674585	2.989719868	1.855836123	2.761149406	D4A2D3
Ubiquitin	Nedd4l	neural precursor cell expressed, develo	20.81092	21.77307	22.21641	23.09427	25.77347	25.9619	26.21645	26.14476	26.31859	26.23929	25.84513	26.63865	+	+	+	3.794419089	4.050478458	3.848593794	4.286746025	F1LRN8
Ubiquitin	Nedd8	neural precursor cell expressed, develo	21.68285	21.945	22.57952	23.10336	27.82098	27.51013	27.6656	28.58244	27.75481	25.26614	27.34875	29.806	+	+	+	5.076080322	5.567102432	2.738394934	5.216235638	Q71UE8
Ubiquitin	Otub1	OTU domain, ubiquitin aldehyde bindin	21.63859	23.92377	23.45615	21.70884	29.27013	28.82548	29.40511	29.49697	29.00948	28.45395	29.47725	29.73365	+	+	+	4.42700976	6.567584515	4.218128437	6.486744404	B2RYG6
Ubiquitin	Pithd1	phosphatidylinositol transfer protein, al	22.76863	22.21708	23.20368	21.90461	26.3782	25.9465	25.82355	25.66226	26.32577	25.54383	25.63719	25.64845	+	+	+	4.356437434	3.42912817	4.134174206	3.265309334	D4ABS5
Ubiquitin	Plaa	phospholipase C, beta 1 (phosphoinositi	20.29703	22.83618	23.45104	22.32795	25.17066	22.95994	26.26712	25.91868	25.85336	22.54223	26.30264	25.52226	+	+	+	1.52080023	2.851052284	1.383219414	2.827073574	P54319
Ubiquitin	Psma1	proteasome (prosome, macropain) subu	21.98689	21.60003	22.5976	22.86916	27.90334	26.75532	26.94626	25.93584	27.88128	26.53903	26.86726	26.18442	+	+	+	4.06229604	4.621770382	4.211597457	4.604579449	P18420
Ubiquitin	Psma2	proteasome (prosome, macropain) subu	22.82262	22.86767	22.50887	23.5341	26.99097	26.86915	22.63923	26.79538	26.63768	26.01521	26.62756	26.61765	+	+	+	1.429536208	2.890369415	4.969165226	3.541211605	P17220
Ubiquitin	Psma3	proteasome (prosome, macropain) subu	22.82077	22.02541	21.81426	23.63514	27.03685	26.26703	26.24989	26.70859	27.33441	26.40437	26.15159	26.65444	+	+	+	3.909303258	3.99169445	3.794489293	4.062306881	P18422;Q6IE67
Ubiquitin	Psma4	proteasome (prosome, macropain) subu	21.85225	22.19129	24.02914	23.19119	26.8527	26.7714	26.97432	26.97301	26.47499	25.59784	27.62527	27.25521	+	+	+	3.75329492	4.076890469	2.966441803	3.92235899	P21670
Ubiquitin	Psma5	proteasome (prosome, macropain) subu	21.28245	23.38835	22.38846	23.05859	27.65581	27.19197	27.53853	27.2049	27.49717	27.09003	26.93247	27.04646	+	+	+	4.275012875	4.868338585	4.134152457	4.612066269	Q6P9V6;P34064
Ubiquitin	Psma6	proteasome (prosome, macropain) subu	24.27829	21.34355	23.25967	23.02328	27.65867	27.33586	27.17655	27.75602	27.88995	27.6807	27.04375	27.60908	+	+	+	3.448477979	4.505578518	3.444388582	4.57967186	P60901
Ubiquitin	Psma7	proteasome (prosome, macropain) subu	21.07067	23.10001	27.69452	26.11231	27.97711	28.41969	28.22991	28.0063	28.29601	28.36982	27.97684	28.32294	+	+	+	1.307794016	3.663873672	1.341530191	3.747024536	F1LSQ6;P48004;P48004-2
Ubiquitin	Psmb1	proteasome (prosome, macropain) subu	21.5879	23.05919	22.47289	22.16464	27.24771	26.21239	26.96598	27.19826	26.91957	25.63118	26.75735	26.93055	+	+	+	4.646611763	4.584932804	4.158128669	4.238508224	Q6PDW4;P18421
Ubiquitin	Psmb2	proteasome (prosome, macropain) subu	21.83609	22.24076	22.7766	24.22854	27.6813	26.46298	27.61246	27.10779	27.57073	27.42534	27.71168	26.94559	+	+	+	3.53228068	4.445636272	3.827384518	4.642839432	P40307
Ubiquitin	Psmb3	proteasome (prosome, macropain) subu	22.39977	21.99626	23.23584	22.40029	26.92785	26.52767	26.66316	26.58248	26.56429	26.70359	26.98348	26.93146	+	+	+	5.280710342	4.16724968	5.323676962	4.287665844	F1LNN1;P40112
Ubiquitin	Psmb5	proteasome (prosome, macropain) subu	22.35395	23.27373	22.23928	22.94314	27.04677	25.68814	26.96785	26.63796	26.82711	25.92857	27.49167	27.2249	+	+	+	4.18479583	3.882653236	4.21442598	4.165537834	G3V7Q6;P28075
Ubiquitin	Psmb6	proteasome (prosome, macropain) subu	21.5142	21.32596	22.35411	22.36839	27.03381	25.93618	26.47065	26.39916	26.80132	26.10173	26.48381	26.54693	+	+	+	4.870014042	4.56928587	5.224413691	4.592782974	P28073
Ubiquitin	Psmd1	proteasome (prosome, macropain) 26S	22.2783	23.28972	25.30921	22.75813	26.34011	25.51542	27.13946	27.13252	27.2179	24.46517	26.40446	27.63851	+	+	+	2.173416817	3.123033524	1.685940683	3.022666931	G3V8B6;O88761
Ubiquitin	Psmd8	proteasome (prosome, macropain) 26S	22.8933	21.9824	23.35	21.96526	25.99833	26.43955	25.87418	25.86674	25.94427	22.44254	26.30438	25.85112	+	+	+	4.099340027	3.496959686	1.436059363	2.58784008	F1LMQ3
Ubiquitin	Tceb1	similar to elongin C; transcription elong	22.75702	22.25941	22.96991	22.53539	27.04552	26.63755	26.10597	25.72091	27.43555	26.45682	26.55498	26.76901	+	+	+	4.563380738	3.747055531	5.354787814	4.173659801	P83941
Ubiquitin	Tceb2	transcription elongation factor B (SIII), p	21.96134	23.38981	21.36712	27.60201	27.41546	28.45352	28.25038	28.56453	28.30343	28.20513	28.17669	28.33087	+	+	+	1.736096096	4.590902805	1.797160972	4.673959732	P62870;F1M937
Ubiquitin	Uba1	ubiquitin-like modifier activating enzym	22.63279	22.05589	23.52382	23.37416	29.36188	30.11434	30.5937	30.57552	30.00089	30.2867	30.83545	30.65395	+	+	+	5.461787452	7.264693737	5.915594872	7.547582626	Q5U300

Supplementary Table 2. List of proteins significantly enriched in both immunoisolated FEZ1 and Kinesin-1 containing vesicles by quantitative mass spectrometry

Group	Gene Symbol	Gene Name	log LFQ intensity IP1 Control	log LFQ intensity IP2 Control	log LFQ intensity IP3 Control	log LFQ intensity IP4 Control	log LFQ intensity IP1 Kinesin-1	log LFQ intensity IP2 Kinesin-1	log LFQ intensity IP3 Kinesin-1	log LFQ intensity IP4 Kinesin-1	log LFQ intensity IP1 FEZ1	log LFQ intensity IP2 FEZ1	log LFQ intensity IP3 FEZ1	log LFQ intensity IP4 FEZ1	t-test Significant Kinesin-1 IP	t-test Significant FEZ1 IP	t-test Significant Kinesin-1 and FEZ1 IP	-Log t-test p value Kinesin-1 IP	t-test Difference Kinesin-1 IP	-Log t-test p value FEZ1 IP	t-test Difference FEZ1 IP	Majority UniProt protein IDs
Ubiquitin	Ube2d3	ubiquitin-conjugating enzyme E2D 3 (UE	21.99563	21.73522	22.85483	22.58742	27.35246	27.44703	28.32693	28.41985	27.36436	27.27959	28.71096	28.30713	+	+	+	5.19030811	5.593291283	4.865381997	5.622233868	M5C9
Ubiquitin	Ube2k	ubiquitin-conjugating enzyme E2K (UBC	20.63055	21.72192	23.07122	22.88588	25.13375	22.49939	25.79094	26.56259	25.68314	24.09974	26.07971	26.82687	+	+	+	1.496896793	2.9192729	2.363398008	3.594971657	D3ZXS8
Ubiquitin	Ube2l3	ubiquitin-conjugating enzyme E2L 3	22.10658	22.47865	22.71381	22.60481	28.32049	28.29802	28.81302	28.8887	28.31605	28.70157	27.98392	29.04903	+	+	+	7.01703699	6.104095459	6.316130384	6.036680698	B2RZA9
Ubiquitin	Ube2m	ubiquitin-conjugating enzyme E2M (UB(	22.59818	23.23791	26.29901	23.87385	26.28905	27.42077	27.20471	26.71725	27.29527	26.55722	26.87115	27.39354	+	+	+	1.854150892	2.90571022	1.965218134	3.02705574	D3ZNQ6
Ubiquitin	Ube2o	ubiquitin-conjugating enzyme E2O	22.50934	22.44712	22.19803	23.22215	26.903	28.19305	26.97301	25.02931	26.79402	27.48777	27.9764	27.93635	+	+	+	3.041695217	4.180429935	5.095690024	4.954473495	F1M403
Ubiquitin	Ubr4	ubiquitin protein ligase E3 component n	24.44275	23.26228	22.60435	22.48239	28.98082	30.16949	29.47079	29.61999	29.17954	29.1322	29.48235	29.40527	+	+	+	4.783930769	6.362330437	4.962306883	6.101899624	F1LQI5;Q2TL32
Ubiquitin	Uchl1	ubiquitin carboxyl-terminal esterase L1	24.19668	27.96074	28.68114	28.46069	31.79071	30.89584	32.00212	31.38915	31.84693	31.06901	32.04999	31.96647	+	+	+	2.087275548	4.194644928	2.192704173	4.408286095	Q00981
Ubiquitin	Uchl3	ubiquitin carboxyl-terminal esterase L3	22.05359	22.87337	22.44744	22.47461	26.21662	25.00881	25.52184	25.49072	25.88215	25.59676	25.61341	25.72617	+	+	+	4.319060884	3.097248077	5.726696627	3.242372036	D4ABI6;Q91Y78
Ubiquitin	Usp5	ubiquitin specific peptidase 5 (isopeptid	21.7122	21.40034	23.2892	23.33451	28.95923	28.84506	29.19784	29.43119	28.81366	28.97525	29.52535	29.40584	+	+	+	4.827091164	6.674266338	4.801243266	6.74596405	D3ZVQ0
Ubiquitin	Usp9x	ubiquitin specific peptidase 9, X-linked	21.92607	22.66946	22.67098	22.00368	29.48629	28.88658	28.72608	28.70971	29.47179	28.15943	29.19873	28.99307	+	+	+	6.487998496	6.634618282	5.86500782	6.638208389	D3ZC84
Unknown	Bcas1	breast carcinoma amplified sequence 1	21.32088	23.01624	21.97311	21.3767	26.8484	22.72939	25.71012	24.3141	26.82141	25.47206	26.28272	26.15864	+	+	+	1.653551618	2.978769779	3.936934328	4.261974335	3;Q3ZB98-4;Q3ZB98-
Unknown	C2cd2l	C2CD2-like	21.96616	23.67527	22.92112	22.15277	27.6238	27.258	27.54363	27.43165	28.22729	27.48308	27.63429	27.64072	+	+	+	4.690224098	4.785439968	4.680313561	5.067518234	Q5U2P5
Unknown	Fam49a	family with sequence similarity 49, mem	22.51218	23.98272	27.69863	23.62342	26.98229	28.01148	27.60562	27.94542	27.22692	27.08152	27.88413	28.1638	+	+	+	1.487128062	3.181966305	1.456625001	3.134857655	B0BN65
Unknown	Fam49b	family with sequence similarity 49, mem	22.47109	22.67261	25.36403	21.50186	28.91806	29.49284	29.22628	29.48978	28.66756	29.27084	29.50506	29.68492	+	+	+	3.532317913	6.279344082	3.481867785	6.279700279	B2GUZ9
Unknown	LOC10036	hypothetical protein LOC100362814	21.84403	22.40185	22.56418	21.55451	26.41857	25.43269	25.68405	25.70604	25.75124	25.12451	25.2959	25.75562	+	+	+	4.632711384	3.719194412	4.664991963	3.390673161	F1LXC7
Unknown	LOC10036	Temporarily Assigned Gene name family	21.41799	25.65892	25.56512	21.85818	26.65349	27.55245	25.5533	26.40903	27.38755	26.98142	26.48061	26.9718	+	+	+	1.265563899	2.917015553	1.538807975	3.330293179	D4A0A1
Unknown	LOC10036	mKIAA0868 protein-like	22.78886	21.98939	20.57653	21.71315	26.25622	22.52484	25.75185	25.88159	26.67425	25.67707	26.53813	26.78918	+	+	+	1.841839735	3.336643696	3.949939364	4.652675152	F1LTJ9
Unknown	LOC36141	similar to Synaptic vesicle membrane pr	21.2981	21.97023	22.82752	22.30718	26.5469	22.74076	25.91552	26.9969	25.8341	22.29213	27.53163	26.79823	+	+	+	1.83915314	3.449263573	1.573487836	3.513264179	D3ZE32;M0R3N4
Unknown	LOC50128	similar to lymphocyte antigen 6 comple:	23.06396	22.8889	23.26042	22.96273	27.25674	26.87033	25.40012	27.44782	27.306	22.68105	26.32939	26.69445	+	+	+	3.649308144	3.699748993	1.383838622	2.708720207	M0R3V4
Unknown	LOC68146	similar to biliverdin reductase B (flavin r	22.30873	22.05054	23.5239	23.31944	26.90885	26.86962	27.92014	27.83563	27.21984	26.87736	27.40965	27.6616	+	+	+	4.208879724	4.582909584	4.522794415	4.491461754	B5DF65
Unknown	LOC68199	similar to AHA1, activator of heat shock	21.74574	28.33786	22.76898	22.26973	26.92502	29.34107	27.91673	28.52216	27.2006	28.16092	27.28233	28.29491	+	+	+	1.46075746	4.395666122	1.352699668	3.954115391	B0BN63
Unknown	LOC68570	similar to neuron navigator 1	22.1335	22.27649	21.7416	24.10692	25.3179	24.46139	24.94985	24.97364	28.39473	25.18643	24.37	25.08821	+	+	+	2.27094469	2.361067295	1.66001649	3.195212364	F1LYK3;F1M031
Unknown	Ly6h	lymphocyte antigen 6 complex, locus H	21.43796	23.36316	22.98347	22.01054	28.36557	26.0055	26.71803	26.21555	28.33313	26.32428	27.07714	27.53237	+	+	+	3.133445301	4.377379417	3.687571042	4.867949963	F1LNW6
Unknown	Mlc1	megalencephalic leukoencephalopathy	23.40137	21.66808	22.26252	23.30729	26.62826	27.27764	27.27959	27.20983	26.73596	26.72403	27.28762	26.88902	+	+	+	4.214186494	4.439015865	4.15358763	4.249345303	D4ABB2
Unknown	Myadm	myeloid-associated differentiation mark	21.60758	22.3274	24.22338	22.29227	26.68204	26.55292	27.022	27.02855	26.46813	25.79233	27.01744	26.78057	+	+	+	3.48002754	4.208716393	3.119339961	3.901958466	Q05BA4;Q6VBQ5
Unknown	Phyhip	phytanoyl-CoA 2-hydroxylase interacting	23.80696	23.19012	22.41637	25.51593	27.37964	28.04354	27.44506	27.50089	27.14676	27.61077	27.7625	27.88704	+	+	+	2.901905743	3.859940529	2.900187543	3.869423866	Q568Z9
Unknown	RGD13048	similar to RIKEN cDNA 6430548M08	22.29438	22.22499	22.66102	22.50989	25.63223	25.19596	26.0451	24.37252	26.2146	22.69349	26.66248	26.70345	+	+	+	3.62338587	2.888881683	1.754333245	3.145936966	D4A3C2
Unknown	RGD13062	similar to KIAA1549 protein	20.9222	22.4921	20.78752	22.41543	27.26249	25.80565	26.77957	25.06011	27.68552	26.73867	27.44782	26.56435	+	+	+	3.292385063	4.572640896	4.279923778	5.45477581	D3Z9D0
Unknown	RGD13072	similar to RIKEN cDNA 2310035C23	23.456	22.10017	27.57701	23.2235	27.04677	27.77662	27.31812	27.68197	27.57528	26.17708	27.51171	27.93399	+	+	+	1.494334672	3.366700172	1.360225935	3.210347176	D3ZJ01
Unknown	RGD13099	similar to mKIAA1244 protein	23.57252	21.56543	22.71085	23.22732	25.01487	27.39681	26.27355	26.41695	26.35384	25.91593	26.22453	26.38014	+	+	+	2.754190596	3.506516933	3.582270479	3.449582577	D3ZF86
Unknown	RGD13108	similar to putative protein (5S487)	22.32089	22.39652	22.08198	22.82535	26.74509	25.12349	25.20288	24.4186	28.23361	26.11912	25.93735	26.29243	+	+	+	2.926568566	2.966331482	3.574961302	4.239442348	D3ZBU7;D3ZEA1
Unknown	RGD15598	similar to mKIAA1045 protein	22.25492	24.04777	22.25304	21.46396	28.48573	26.25308	28.58978	28.28092	28.93106	26.27673	28.71571	28.64581	+	+	+	3.351630247	5.397457123	3.301189822	5.63740921	D3ZBS6
Unknown	RGD15608	similar to plexin 1	21.39972	21.64161	22.72413	23.10377	29.99766	28.12829	29.77331	29.52026	29.74021	28.849	29.64607	29.73358	+	+	+	4.717100476	7.137572289	5.362098488	7.274909019	D3Z981
Unknown	Spryd7	SPRY domain containing 7	23.48384	22.28606	21.45985	23.42425	25.92936	26.15339	26.06964	26.59092	25.90226	25.49454	25.73629	25.91563	+	+	+	3.354347686	3.52232933	3.105774583	3.098684788	Q5M7T2
Unknown	Syap1	synapse associated protein 1, SAP47 ho	27.74163	26.35966	27.3132	26.2335	23.91056	21.64842	24.62906	22.6139	25.03938	22.64522	22.70939	21.69829	+	+	+	2.562541677	-3.71151066	2.550326163	-3.888924599	Q6AYB6
Unknown	Them6	thioesterase superfamily member 6	22.77688	22.79097	20.88644	22.29761	25.8328	24.66199	25.894	26.2105	25.75223	26.31541	25.50209	26.06343	+	+	+	3.072902906	3.461848259	3.602109428	3.720315933	Q5XIE1
Unknown	Tmem163	transmembrane protein 163	21.48105	22.09041	22.56487	22.51508	26.11446	27.72416	25.82435	25.95098	26.01729	25.33541	25.78339	26.25845	+	+	+	3.783878922	4.240635395	4.59862674	3.685783386	A9CMA6
Unknown	Tmem33	transmembrane protein 33	22.85328	22.89445	22.07688	22.16923	26.11345	26.7375	25.83145	26.13848	26.43333	26.58792	25.68078	26.86726	+	+	+	4.857006924	3.70675993	4.617943636	3.893862247	Q9Z142
Unknown	Tmx2	thioredoxin-related transmembrane prc	21.55467	22.09303	22.25226	22.66591	26.76547	26.98294	26.24382	26.88658	26.5979	26.36986	26.79885	26.96565	+	+	+	5.451846002	4.578233719	5.612973128	4.541596889	Q5XIK2
Unknown	Tmx4	thioredoxin-related transmembrane prc	22.08765	22.11673	22.2328	23.70559	25.88481	26.75901	26.38265	26.12949	26.24302	26.24153	26.08552	25.4706	+	+	+	3.883050339	3.753297329	3.70342566	3.474473476	G3V912
Unknown	Zzef1	zinc finger, ZZ-type with EF-hand domai	21.5204	22.38955	21.72983	22.98385	24.50767	26.81568	25.3584	25.68504	23.79313	25.09495	25.60688	25.60752	+	+	+	2.979560117	3.435789108	2.735173613	2.869710445	D3ZG78

Supplementary Table 3. List of kinases tested for activity against FEZ1 Serine-58

#	ProQinase	Kinase	In IP-MS?	Activity against S58?
1	ACV-R1	ACVR1	N	N
2	ACV-R1B	ACVR1B	N	N
3	ACV-RL1	ACVRL1	N	N
4	AKT1	AKT1	N	N
5	AKT2	AKT2	N	N
6	AKT3	AKT3	N	N
7	AMPK-alpha1	PRKAA1	N	N
8	ARK5	NUAK1	N	Y
9	ASK1	MAP3K5	N	N
10	Aurora-A	AURKA	N	N
11	Aurora-B	AURKB	N	N
12	Aurora-C	AURKC	N	N
13	B-RAF VE	BRAF	N	N
14	B-RAF wt	BRAF	N	N
15	BRSK1	BRSK1	N	N
16	CAMK1D	CAMK1D	N	N
17	CAMK2A	CAMK2A	Y	N
18	CAMK2B	CAMK2B	N	N
19	CAMK2D	CAMK2D	N	N
20	CAMK4	CAMK4	N	N
21	CAMKK2	CMAKK2	N	N
22	CDC42BPA	CDC42BPA	N	N
23	CDC42BPB	CDC42BPB	Y	N
24	CDK1/CycA	CDK1	N	N
25	CDK1/CycE	CDK1	N	N
26	CDK1CycB1	CDK1	N	N
27	CDK2/CycA	CDK2	N	N
28	CDK2/CycE	CDK2	N	N
29	CDK3/CycE	CDK3	N	N
30	CDK4/CycD1	CDK4	N	N
31	CDK4/CycD3	CDK4	N	N
32	CDK5/p25NCK	CDK5	N	N
33	CDK5/p35NCK	CDK5	N	N
34	CDK6/CycD1	CDK6	N	N
35	CDK7CycH	CDK7	N	N
36	CDK8/CycC	CDK8	N	N
37	CDK9/CycT	CDK9	N	N
38	CHK1	CHEK1	N	N
39	CHK2	CHEK2	N	N
40	CK1-alpha1	CSNK1A1	N	N
41	CK1-delta	CSNK1D	N	N
42	CK1-epsilon	CSNK1E	N	N
43	CK1-gamma1	CSNK1G1	N	N
44	CK1-gamma2	CSNK1G2	N	N
45	CK1-gamma3	CSNK1G3	N	N
46	CK2-alpha1	CSNK2A1	N	N
47	CK2-alpha2	CSNK2A2	N	N
48	CLK1	CLK1	N	N

Supplementary Table 3. List of kinases tested for activity against FEZ1 Serine-58

#	ProQinase	Kinase	In IP-MS?	Activity against S58?
49	CLK2	CLK2	N	N
50	CLK3	CLK3	N	N
51	COT	MAP3K8	N	Y
52	DAPK1	DAPK1	N	N
53	DAPK2 (+CaM)	DAPK2	N	N
54	DAPK3	DAPK3	N	N
55	DCAMKL2	DCLK2	N	N
56	DMPK	DMPK	N	N
57	DYRK1A	DYRK1A	N	N
58	DYRK1B	DYRK1B	N	N
59	DYRK3	DYRK3	N	N
60	EIF2AK2	EIF2AK2	N	N
61	EIF2AK3	EIF2AK3	N	N
62	ERK1	MAPK3	Y	N
63	ERK2	MAPK1	Y	N
64	GRK2	ADRBK1	N	N
65	GRK3	ADRBK2	N	N
66	GRK4	GRK4	N	N
67	GRK5	GRK5	N	N
68	GRK6	GRK6	N	N
69	GRK7	GRK7	N	N
70	GSK3-alpha	GSK3A	N	N
71	GSK3-beta	GSK3B	N	N
72	HIPK1	HIPK1	N	N
73	HIPK3	HIPK3	N	N
74	HRI	EIF2AK1	N	N
75	IKK-alpha	CHUK	N	N
76	IKK-beta	IKBKB	N	Y
77	IKK-epsilon	IKBKE	N	Y
78	IRAK1	IRAK1	N	N
79	IRAK4	IRAK4	N	N
80	JNK1	MAPK8	N	N
81	JNK2	MAPK9	N	N
82	JNK3	MAPK10	Y	N
83	LIMK1	LIMK1	N	N
84	LRRK G2019S	LRRK2	N	N
85	MAP4K2	MAP4K2	N	N
86	MAP4K4	MAP4K4	N	N
87	MAP4K5	MAP4K5	N	N
88	MAPKAPK3	MAPKAPK3	N	N
89	MAPKAPK5	MAPKAPK5	N	N
90	MARK1	MARK1	Y (FEZ1 IP)	Y
91	MARK2	MARK2	Y	Y
92	MARK3	MARK3	Y (FEZ1 IP)	Y
93	MEK1 wt	MAP2K1	N	N
94	MELK	MELK	N	N
95	MINK1	MINK1	Y	N
96	MKK6SDTD	MAP2K6	N	N

Supplementary Table 3. List of kinases tested for activity against FEZ1 Serine-58

#	ProQinase	Kinase	In IP-MS?	Activity against S58?
97	MST1	STK4	N	N
98	MST2	STK3	N	N
99	MST3	STK24	N	N
100	MST4	MST4	N	N
101	mTOR	MTOR	Y	N
102	MYLK2	MYLK2	N	N
103	NEK1	NEK1	N	N
104	NEK11	NEK11	N	N
105	NEK2	NEK2	N	N
106	NEK3	NEK3	N	N
107	NEK4	NEK4	N	N
108	NEK6	NEK6	N	N
109	NEK7	NEK7	N	N
110	NEK9	NEK9	N	N
111	NIK	MAP3K14	N	Y
112	NLK	NLK	N	N
113	p38-alpha	MAPK14	N	N
114	p38-beta	MAPK11	N	N
115	p38-delta	MAPK13	N	N
116	p38-gamma	MAPK12	N	N
117	PAK1	PAK1	N	N
118	PAK2	PAK2	N	N
119	PAK3	PAK3	N	N
120	PAK4	PAK4	N	Y
121	PAK6	PAK6	N	N
122	PAK7	PAK7	N	N
123	PASK	PASK	N	N
124	PBK	PBK	N	N
125	PCTAIRE1	CDK16	N	N
126	PDK1	PDPK1	N	N
127	PHKG1	PHKG1	N	N
128	PHKG2	PHKG2	N	N
129	PIM1	PIM1	N	N
130	PIM2	PIM2	N	N
131	PIM3	PIM3	N	N
132	PKA	PRKACA	Y	N
133	PKC-alpha	PRKCA	Y	N
134	PKC-beta1	PRKCB	Y	N
135	PKC-beta2	PRKCB	Y	N
136	PKC-delta	PRKCD	Y	N
137	PKC-epsilon	PRKCE	Y	N
138	PKC-eta	PRKCH	N	N
139	PKC-gamma	PRKCG	Y	N
140	PKC-iota	PRKCI	N	N
141	PKC-mu	PRKD1	N	N
142	PKC-nu	PRKD3	N	N
143	PKC-theta	PRKCQ	N	N
144	PKC-zeta	PRKCZ	N	N

Supplementary Table 3. List of kinases tested for activity against FEZ1 Serine-58

#	ProQinase	Kinase	In IP-MS?	Activity against S58?
145	PLK1	PLK1	N	N
146	PLK3	PLK3	N	N
147	PRK1	PKN1	N	N
148	PRK2	PKN2	N	N
149	PRKD2	PKD2	N	N
150	PRKG1	PRKG1	N	N
151	PRKG2	PRKG2	N	N
152	PRKX	PRKX	N	N
153	RAF1 DYDY	RAF1	N	N
154	RIPK2	RIPK2	N	N
155	ROCK1	ROCK1	N	N
156	ROCK2	ROCK2	N	N
157	RON	MST1R	N	N
158	RPS6KA1	RPS6KA1	N	N
159	RPS6KA2	RPS6KA2	N	N
160	RPS6KA3	RPS6KA3	N	N
161	RPS6KA4	RPS6KA4	N	N
162	RPS6KA5	RPS6KA5	N	N
163	RPS6KA6	RPS6KA6	N	N
164	S6K	RPS6KB1	N	N
165	S6K-beta	RPS6KB1	N	N
166	SAK	PLK4	N	N
167	SGK1	SGK1	N	N
168	SGK2	SGK2	N	N
169	SGK3	SGK3	N	N
170	SNARK	NUAK2	N	Y
171	SNF1LK2	SNF1LK2/SIK2 (Human)	N	N
172	SNK	PLK2	N	N
173	SRPK1	SRPK1	N	N
174	SRPK2	SRPK2	N	N
175	STK17A	STK17A	N	N
176	STK23	SRPK3	N	N
177	STK33	STK33	N	N
178	TAOK2	TAOK2	N	N
179	TAOK3	TAOK3	N	N
180	TBK1	TBK1	N	Y
181	TGFB-R1	TGFBR1	N	N
182	TGFB-R2	TGFBR2	N	N
183	TSF1	STK16	N	N
184	TSK2	TSSK2	N	N
185	TSSK1	TSSK1B	N	Y
186	TTK	TTK	N	N
187	VRK1	VRK1	N	N
188	WNK2	WNK2	Y (FEZ1 IP)	N
189	WNK3	WNK3	N	N
190	ZAK	ZAK	N	N
